# Dissolution and regeneration of nylons utilizing superbase-acid conjugate ionic liquids

**DOI:** 10.1039/d5ra07715j

**Published:** 2025-12-03

**Authors:** Eva Gazagnaire, Yuliia Bardadym, Sami Hietala, Sami-Pekka Hirvonen, Vladimir Aseyev, Ilkka Kilpeläinen, Aleksandar R. Todorov

**Affiliations:** a Materials Chemistry Division, Department of Chemistry, University of Helsinki 00560 Helsinki Finland aleksandar.todorov@helsinki.fi Ilkka.kilpelianen@helsinki.fi

## Abstract

Synthetic polyamides (nylons) are widely used in our daily life and in technical applications due to the good mechanical properties, as well as good resistance towards chemicals and sunlight. The recycling of nylons is challenging even as pure materials, however their structural diversity further complicates the recycling of mixed nylon waste. The mechanical strength and stiffness of nylons arise from interchain hydrogen bonding of the polymer chains resulting poor solubility in common solvents. The solubility of cellulose is also hindered by a strong hydrogen bonding network, yet it is well known that many superbase ionic liquids (SB-ILs) are efficient for dissolution and regeneration of cellulose. It turns out that many of the known cellulose dissolving SB-ILs can also dissolve nylon polymers, but efficiency increases towards more hydrophobic superbase cations. From the screened SB-ILs, the 5/7-methyl-3,3-dimethyl-1,5,7-triazabicyclo[4.4.0]dec-5-enium acetate ([dm_3_-mTBDH][OAc], a mixture of isomers) showed good performance in a dissolution-regeneration process of nylons. No degradation of the regenerated nylon polymer was observed upon comparison with the virgin ones and the SB-IL was stable through repeated dissolution-regeneration cycles. The solubility of nylons in SB-ILs enable novel tools for characterization of nylon polymers and opens possibilities towards efficient and sustainable recycling of nylons.

## Abstract 2

Nylon is efficiently dissolved and regenerated using superbase ionic liquids, enabling sustainable reuse with preserved material properties.

## Graphical abstract

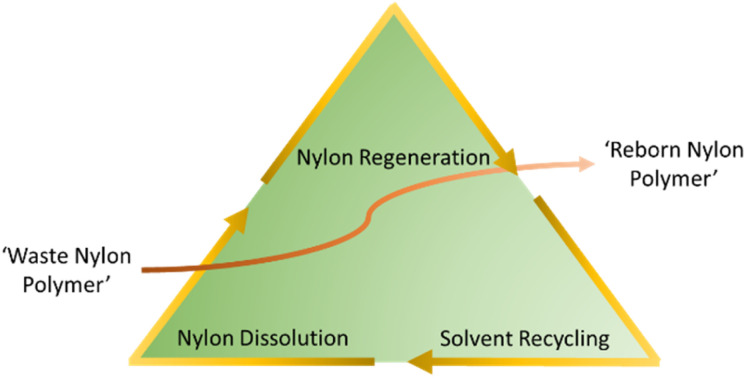

## Introduction

Initially invented by the DuPont company, nylons remain one of the most utilized polymeric materials, due to their excellent physical properties and relatively low manufacturing costs.^1^ The term nylon is a collective name for a class of thermoplastic aliphatic synthetic polymers containing amide groups as the repeating functionalities. Nylons are commercially prepared from diamines and dicarboxylic acids with variable chain lengths (nylon 6.6, nylon 6.12, *etc.*), or from only one monomer *e.g.*, aminocarboxylic acids or lactams (nylon 6, nylon 12, *etc.*).^2^

The application areas of nylons vary from technical applications in bushings or bearings to daily consumables in clothing or stockings, and to home appliances, fishing nets *etc.* The variety of applications arise from the structural diversity of nylon polymers that provides a rather unique platform for tuning their physical properties, *e.g.* tensile elongation, flexural strength, and modulus of elasticity. In addition to the repeating units, the technical properties of nylons are further affected by the degree of polymerization (DP), the molar mass distribution (MMD), and the purity of the polymer.^3^

While the fabrication of nylons is relatively easy and economical, the recycling of these materials has challenges.^4^ The post-industrial waste is often relatively pure and may consist of only one type of nylon material and can thus be efficiently recycled thermomechanically or chemically. In thermomechanical recycling, some deterioration of the technical properties of the polymer may happen,^5^ nevertheless the recycled material can be blended with virgin polymer to keep the end-product properties at the desired level. As such, the chemical recycling process allows for a ‘fresh start’ as a reborn polymer and yet it may suffer from lower overall yield.^6^ However, recycling of nylons from the post-consumer waste is difficult, as its relative amount in the bulk plastic waste is low and due to its structural diversity. Nylon-rich fractions can be isolated in some plastic waste recycling processes, yet as the composition of these fractions varies, these are often not utilized but discarded for combustion or disposed at landfill sites.^7^

The good mechanical and chemical properties of nylons originate from the intermolecular hydrogen bonding between the polymer chains, however the hydrogen bonding also makes the dissolution of nylons challenging. For efficient dissolution of nylon, the interchain hydrogen bonding network needs to be somehow disrupted or inhibited. Only a limited number of solvents are known for dissolution of nylons.^7^ Some highly protic solvents (*e.g.*, 3-methylphenol (m-cresol), 1,1,1,3,3,3-hexafluoro-2-propanol (HFIP), 2,2,2-trifluoroethanol (TFE)) can interact with the amide functionalities of nylon allowing dissolution of nylon *via* disturbing the hydrogen bonding network by competition with the interchain hydrogen bonds of the polymer.^8–13^ Additionally, highly acidic solvent systems, possibly partially protonating the amide functionalities, are known to dissolve nylons. These acidic systems include concentrated mineral or strong organic acids *e.g.*, sulfuric, formic or trifluoro acetic acid (TFA) as such or together with some common solvents (acetone, dichloromethane).^14–17^

Chemical stability and poor solubility are usually beneficial during the intended use of nylons and yet can consequently hinder the efficient recycling of the waste material, as well as restrict means for characterization of the polymer. There exist efficient methods for bulk characterization of physical and chemical properties of nylon materials, however tools to assess detailed polymer characteristics (DP, MMD, polydispersity with GPC or SEC) or structure (like liquid state NMR) of nylon polymers are limited.^16,18–21^

The difficulties in dissolution of nylons resembles the complications of cellulose dissolution. For cellulose, there has been a rapid development of several types of dissolution approaches to enable the utilization of this abundant material for example in manufacturing man-made cellulosic textile fibres. In this connection, several types of ionic liquids have been developed for efficient dissolution of cellulose.^22,23^ In some examples, ionic liquids have also been applied^24–28^ to separate cellulose from cellulose/nylon mixtures by selective dissolution of cellulose,^29^ or to depolymerize nylon.^30–33^

The similarities in the characteristics between cellulose and nylons prompted us to try the dissolution of different types of nylons with SB-ILs, which are not only well-established solvents for cellulose,^22,34–40^ but also environmentally safe and classified as not-harmful.^41^ Herein, we present the dissolution of nylons utilizing SB-ILs.

## Results and discussion

### Dissolution of nylons in SB-ILs

**Chart 1:**
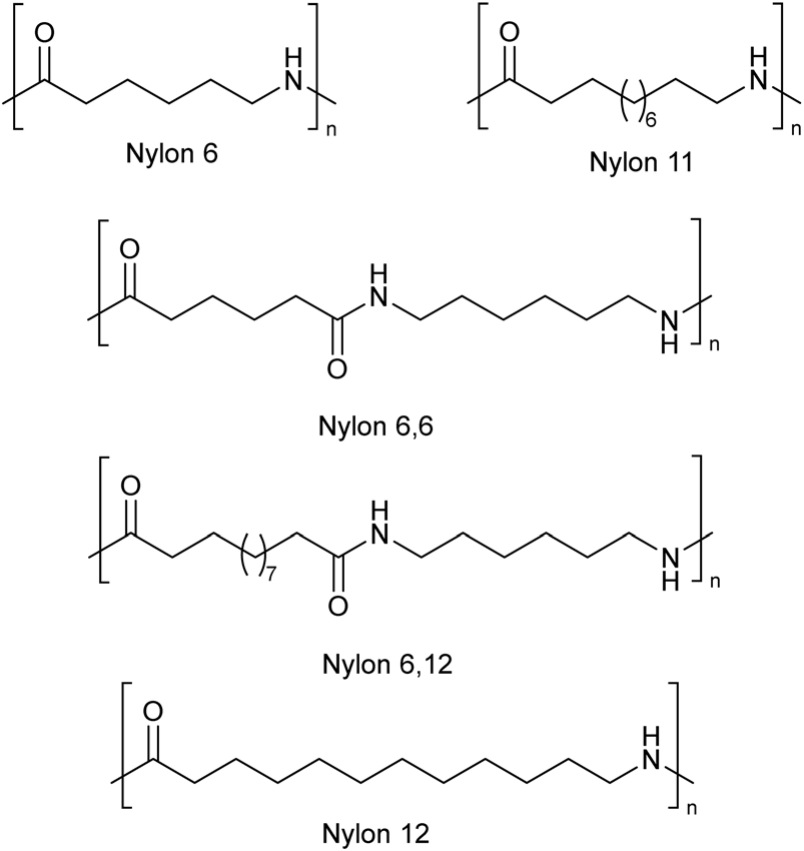
Selected types of nylon polymers 6, 6.6, 6.12, 11 and 12 for dissolution/solubility tests. Nylon 6.6 was obtained as pure material (6.6 P) and as a mixture of nylon 6.6 and proprietary impact modifier (6.6 M).

The structural diversity of nylon polymers used in the textile and plastics industry is large, and commercial materials may also contain modifiers designed to improve technical properties, like impact resistance. For the initial solubility tests, common nylon polymers/materials were selected (Chart 1).^4^ The nylon samples were received as pellets and used as such for the dissolution experiments (details in SI). The dissolution (solubility) of polymers is a complicated phenomenon, as there may still exist aggregation or formation of organized structures in the solution-like state. Nevertheless, in the current study a simplified approach was utilized, where solubility is understood as formation of homogenous liquid state (no residual particles or opaqueness) with liquid flow properties (no gel formation or any indications for separate phases). During the solubility tests optical microscopy was used as a primary tool for verification of the nylon polymers dissolution into the used SB-ILs.

**Chart 2:**
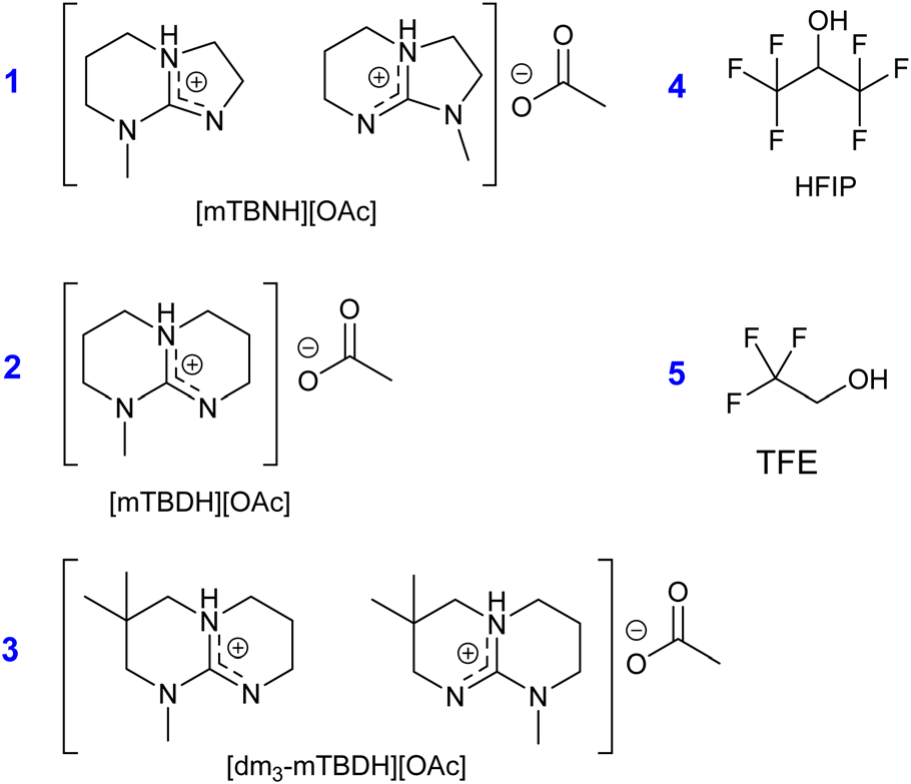
Structural representation of selected nylon solvents, where 1:5/7-methyl-1,5,7-triazabicyclo[4.3.0]non-5-enium acetate [mTBNH][OAc] (mixture of isomers); 2: 7-methyl-1,5,7-triazabicyclo[4.4.0]dec-5-eneium acetate [mTBDH][OAc]; 3:5/7-methyl-3,3-dimethyl-1,5,7-triazabicyclo[4.4.0]dec-5-enium acetate [dm_3_-mTBDH][OAc] (mixture of isomers); 4:1,1,1,3,3,3-hexafluoro-2-propanol (HFIP); 5:2,2,2-trifluoroethanol (TFE).

After initial screening with various SB-ILs (originally prepared for cellulose dissolution/regeneration),^37^ three SB-ILs (Chart 2) were selected for further study. Two of these SB-ILs are efficient solvents for cellulose (1 and 2, Chart 2). However, the third SB-IL (3, Chart 2) is not efficient solvent for cellulose, but dissolves cellulose as a mixture with DMSO.^37^ This behaviour has been attributed to the higher hydrophobicity of 3 as compared to the SB-ILs 1 and 2. The increasing hydrophobicity of bicyclic guanidine cation in SB-IL decreases solubility of cellulose, along with decreasing SB-IL miscibility with water and increasing hydrolytic stability.^37^ Already during initial tests, the SB-IL 3 showed high dissolution efficiency for nylon samples.

To put the dissolution of nylons into SB-ILs in perspective, the dissolution capabilities were compared with the known nylon solvents HFIP and TFE (Chart 2). These classical nylon solvents were selected as benchmarks due to their well-documented efficacy.^7^

Among other things, the solubility of polymers is usually strongly dependent on concentration of the polymer. The solubility tests for nylon samples were conducted with 10% (by mass) polymer concentrations, which is adequate also for larger scale applications.^7^

For solubility of small molecules, the solubility and dissolution speed increases with increasing temperature. This is especially the case with viscous solvents, as the speed of dissolution increases with decreasing viscosity at higher temperatures. From this perspective, it would be advantageous to increase the dissolution temperature closer to the degradation temperature of the sample (or solvent). However, in analogue with cellulose, the solubility of nylon polymers is to a good extent dependent on braking down interchain hydrogen bonding. Therefore, the dissolution efficiency may benefit from lower temperatures, as the solvent competition with interchain hydrogen bonding becomes more favourable. As a consequence of these concurrent phenomena, the solubility of these type of ‘hydrogen bonding’ polymers often has a turning point (optimum) temperature, where the solubility and dissolution speed reaches an adequate compromise. With the well-known cellulose dissolving SB-ILs (1 and 2) the optimal temperature for cellulose dissolution is 80–90 °C, which was also used as a starting point for dissolution trials of nylon polymers. It turned out that the optimal temperature for all tested nylon polymers was slightly higher (∼100 °C), albeit not fully optimized for different combinations of the SB-ILs and nylon samples.

**Fig. 1:**
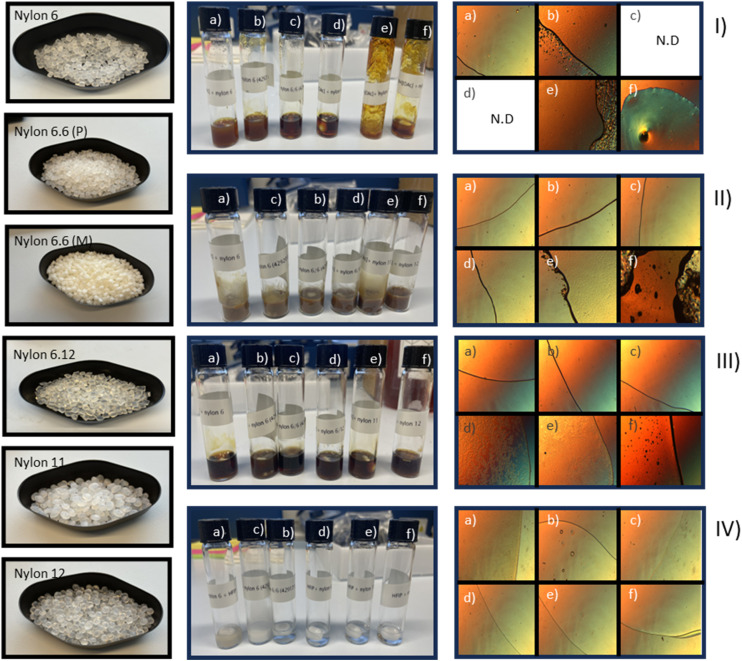
First column: general view of nylon materials; middle column: general view of the dissolved nylon samples (10% (by mass)); last column: microscope pictures of the nylon dissolution experiments in I) [mTBNH][OAc], II) [mTBDH][OAc], III) [dm_3_-mTBDH][OAc] and IV) HFIP, where (a) nylon 6, (b) nylon 6.6 P, (c) nylon 6.6 M, (d) nylon 6.12, (e) nylon 11, and (f) nylon 12. All pictures are at 4 × magnification and have been taken at room temperature at the exception of [mTBDH][OAc] solutions due to their higher melting point. For higher resolution microscope pictures see SI.

The further dissolution tests of different nylon polymers with 1 were not encouraging (Fig. 1, middle and last column row I). This SB-IL partially dissolved the nylons with the shortest carbon atom chain length (nylon 6 and 6.6 P) at 100 °C, resulting with beads with reduced size after 20 hours, yet still visible by naked eye, indicating rather low solubility concentrations. However, the texture of these solutions was closer to a gel rather than a solution, and the samples turned opaque at room temperature.

Another cellulose dissolving SB-IL, 2 showed rather good dissolution properties for various nylon polymers at 100 °C (Fig. 1, middle and last column row II). However, 2 is solid at room temperature (m.p. 82–83 °C) and the dissolved nylon samples solidified at temperatures below 60 °C. This complicates the handling of the dissolved samples and is not ideal for any possible larger scale applications. Nylon 6 and nylon 6.6 (P and M) dissolved into 2 (Fig. 1, middle and last column row II). In the case of nylon 6.12, the solution was almost clear, however few nylon particles or aggregates were still visible under microscope after 20 hours dissolution time. However, nylons 11 and 12 formed a gel in 2 along with some remaining pieces of undissolved particles. It is likely that lowering the polymer concentration from 10% (by mass) would yield better solubility. Despite the rather good nylon dissolution capability of 2, its higher melting point makes the handling of these solutions somewhat cumbersome.

The SB-IL 3 showed very good dissolution efficiency for all tested nylon polymers (Fig. 1, middle and last column row III), Upon cooling to room temperature, the nylon 6, 6.6 P and 6.6 M samples remained as clear solutions. However, the nylon 6.12, 11 and 12 samples adopted a ‘gel’ like texture upon cooling to room temperature and showed a transition to clear liquids upon re-heating to 60 °C.

During the series of dissolution experiments, some darkening in the colour of the nylon samples dissolved in SB-ILs took place (Fig. 1, middle column row I–III). This phenomenon is common when a SB-IL is heated for several hours.^23^ The origin of this colour formation remains still unclear, as no degradation of the utilized SB-ILs was detected by NMR (Fig. S67 and S68).

HFIP and TFE are established solvents for nylons and were used as references. Due to their low boiling point (60 °C and 74 °C respectively), these tests were made at 40 °C (Fig. 1, middle and last column row IV). Unexpectedly, TFE dissolved only nylon 6 and nylon 6.6 P and was thus omitted from further studies. On contrary, HFIP dissolved most of the samples (nylon 6.6 P, 6.12, 11 and 12) as expected, however nylon 6 and nylon 6.6 M samples remained cloudy and had remaining particles. The difference in solubility of nylon 6.6 M and 6.6 P in HFIP is somewhat surprising, but likely the impact modifier in 6.6 M interferes with the solubility of the polymer.

**Scheme 1:**
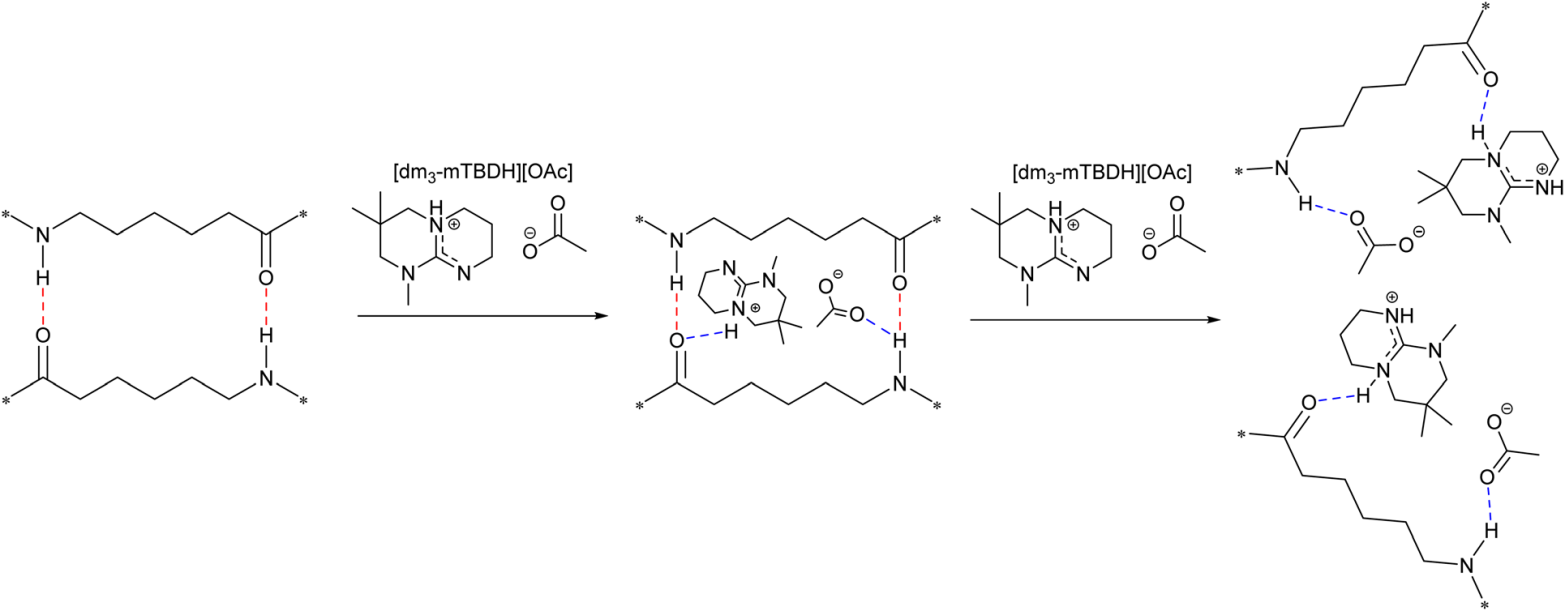
Proposed dissolution mechanism of nylon in SB-IL. The mechanism goes through a breaking of the hydrogen bonding system between the amide functionalities. This can be dependent on the structure of the SB cation and the nature of the anion (*i.e.* ability to compete for hydrogen binding).

Based on the obtained dissolution data, a plausible dissolution mechanism for nylons in SB-ILs can be proposed (Scheme 1). Presumably, the amphiphilic SB-ILs interacts with the nylon's intermolecular hydrogen bond network, primarily involving the amide functionalities (–N–H⋯O=C–), thus leading to their disruption. Likewise to the dissolution of cellulose,^42^ the acetate anion in the SB-ILs plays a central role in the dissolution of the nylon polymers by interacting with their amide functionality. After the intermolecular hydrogen bond networks are disrupted, the polar yet hydrophobic character of the superbase cation further facilitates the solubilization of the dissociated nylons' polymer chains. Although this is simplified and general mechanistic explanation, due to the complexity of the system, no detailed molecular simulations were conducted.

### Regeneration and characterization of dissolved nylons

As discussed above, SB-IL 3 showed very good dissolution efficiency for the different types of nylon polymers. Therefore SB-IL 3 was subjected for further studies on characterization of the regenerated polymers, as well as recycling experiments of the SB-IL. Additionally, HFIP solutions were used as reference in some cases. Further, ethanol was used as an antisolvent to precipitate the dissolved nylons, as it was an efficient antisolvent for both 3 and HFIP solutions. Unlike water, ethanol mixes well with the SB-IL and was easy to remove by evaporation.

NMR spectroscopy was used to provide data on the behaviour of both the solute and the solvent, *i.e.* to reveal possible degradation or reactions of the polymer, or possible formation of impurities from the polymer or the SB-IL. To investigate if the dissolution-regeneration treatment affected the recovered nylon materials, both the virgin nylon samples and recovered polymers from 3 and HFIP were analysed with NMR, SEC and FT-IR.

The ^1^H NMR spectra of nylons dissolved in 3 is dominated by the signals of SB-IL. For nylon-6, comparison of ^1^H spectrum in HFIP and diffusion edited ^1^H spectrum in 3 shows typical signals of a dissolved polymer (Fig. S21). For other nylon polymers, this direct approach was not straightforward due to high viscosity of the samples. In many cases, the dissolved polymer solution in SB-IL can be diluted with a co-solvent to adjust the viscosity.^43^ However, for nylons dissolved in 3, no suitable co-solvents were identified (Fig. S22). Thus, we opted to isolate the regenerated polymer samples, followed by re-dissolution into HFIP for a detailed NMR analysis, to investigate if the dissolution-regeneration treatment affected the recovered nylon materials (see SI). The recovered SB-IL 3 was also subjected to NMR for signs of degradation, nylon retaining, or accumulation of low molecular mass nylon fragments.

**Fig. 2:**
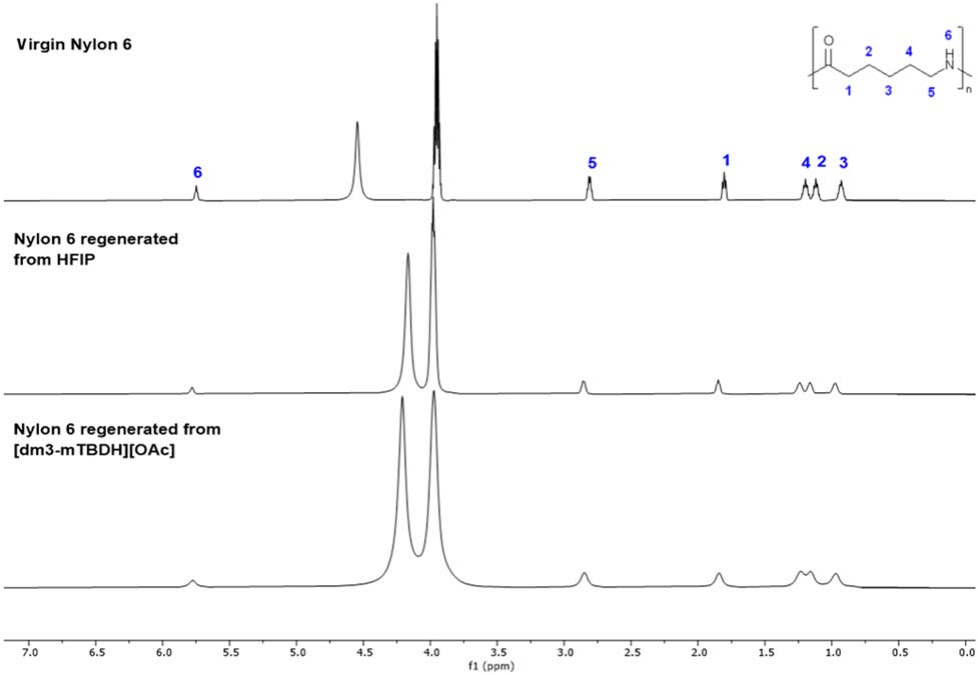
^1^H NMR in HFIP of virgin nylon 6 *vs.* regenerated nylon 6 from HFIP and [dm_3_-mTBDH][OAc]. The two intense signals originate from the solvent HFIP.

The ^1^H NMR spectra, for example of virgin nylon 6 and the regenerated one from the SB-IL 3 or the HFIP, showed no differences (Fig. 2). Importantly, there were no signs of entrapment of the SB-IL, nor the ethanol (anti-solvent) into the nylon matrix upon regeneration. Further NMR analysis showed no discrepancies between the virgin nylons and the regenerated ones (both from 3 and HFIP). Moreover, no changes were detected in the diffusion coefficients for any regenerated nylon samples compared to the virgin nylon samples (Fig. S23–S28).

In some cases, IR spectroscopy is sensitive in detecting changes for example in hydrogen bonding or crystallinity. The FT-IR spectra of nylon samples were identical for native and regenerated samples and thus confirmed that the dissolution-regeneration cycles did not introduce any chemical structure changes into the nylon polymers (Fig. S31–S36).

**Fig. 3:**
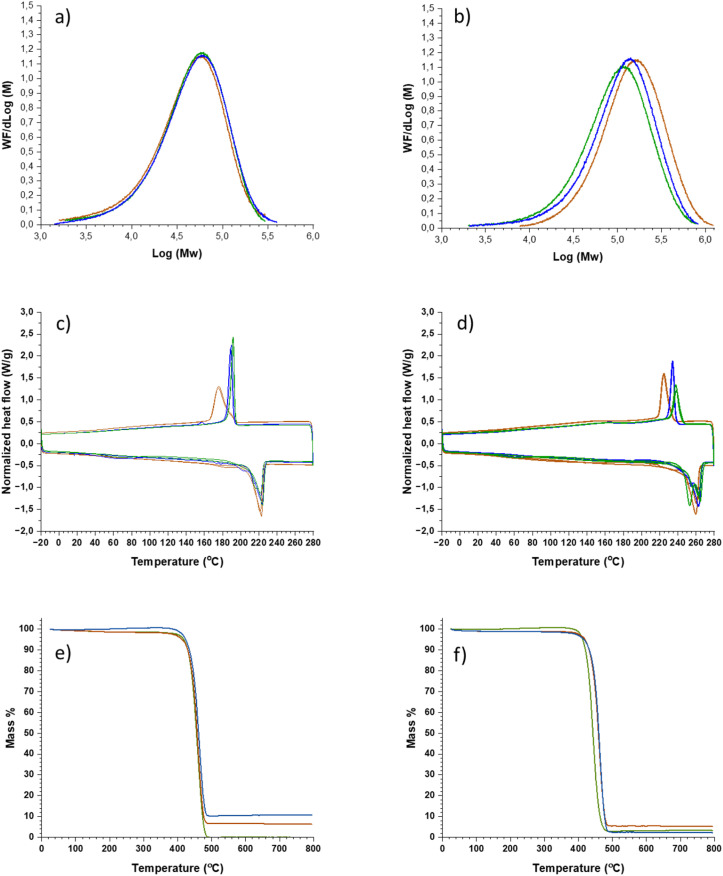
SEC chromatography, DSC and TGA analysis of different nylons (a) SEC of nylon 6, (b) SEC of nylon 6.6 P, (c) DSC of nylon 6, (d) DSC of nylon 6.6 P, (e) TGA of nylon 6, and (f) TGA of nylon 12. In all cases green line denotes regenerated form [dm_3_-mTBDH][OAc], blue line from HFIP, and light brown line the corresponding virgin nylon.

Size exclusion chromatography (SEC) was employed to assess possible changes in molecular weight during the dissolution/regeneration process. The SEC measurements showed only minor changes in all cases with exception for nylon 6.6 P (Fig. 3a, b, S39–S43 and Table S1). For nylon 6.6 P, there was a clear decrease both in the molar mass and polydispersity in both solvents (SB-IL 3 and HFIP). An explanation for this can be the presence of low mass nylon fragments or other low mass impurities present in the nylon sample. Supporting this interpretation, small amounts of unknown impurities were found in the recovered SB-IL by ^1^H NMR (Fig. S69). These small molecular weight impurities might originate from the polymer precursors used in the synthesis of nylon 6.6 (P).

Both spectroscopic (NMR, FT-IR) and SEC results show that the dissolution-regeneration cycle did not alter the chemical structure of nylons in HFIP or SB-IL 3. However, despite no chemical differences were detected during the dissolution-regeneration of nylons, the process might change the physical properties of the materials. Nylons are semi-crystalline polymers which means that the dissolution-regeneration cycle may alter their crystallinity, even though no changes were detected in the FT-IR analysis. Nevertheless, the possible changes in crystallinity can impact the physical properties of the regenerated materials (like stiffness, temperature resistance, elasticity, *etc.*).

To assess the thermal stability of the regenerated nylons thermal gravimetric analysis (TGA) were performed. For most of the samples, the TGA behaviour remained the same as to the virgin nylons (Fig. 3e, S46–S51 and Table 1). However, both nylon 11 and 12 showed a clear increase in their degradation temperatures after regeneration from 3 (nylon 11) and HFIP (both) (Fig. 3f, S50, S51 and Table 1). The increase in the degradation temperatures (21 °C for nylon 12 isolated from SB-IL 3) suggests a higher degree of crystallinity. To verify this finding differential scanning calorimetry (DSC) was used.

**Table 1:** Tabulated data from TGA and DSC analysis

Nylon	Origin^a^	*T*_onset_ (°C)	*T*_g_ (°C)	*T*_m_ (°C)	*T*_c_ (°C)
Nylon 6	Virgin	418	53	221	176
	3	417	55	222	190
	HFIP	416	56	222	192
Nylon 6.6 (P)	Virgin	406	51	260	225
	3	405	54	263	234
	HFIP	402	52	262	238
Nylon 6.6 (M)	Virgin	399	138	262	235
	3	402	135	264	238
	HFIP	397	140	265	240
Nylon 6.12	Virgin	434	41	217	183
	3	440	38	218	192
	HFIP	437	39	217	192
Nylon 11	Virgin	408	44	187	147
	3	442	45	190	160
	HFIP	410	47	183	166
Nylon 12	Virgin	422	34	178	135
	3	443	34	179	154
	HFIP	439	36	179	155

a Origin refers to the solvent used for dissolution.

Notably, DSC is sensitive to even small physical changes in material properties, *i.e.* the thermal response of nylons during heating and cooling cycles is dependent on the crystallinity of the polymer, its molecular weight or presence of additives or impurities. The DSC data show that most of the performed dissolution-regeneration cycles of the different nylons did not induce detectable changes in their material properties (Fig. 3c, d, S55–S59 and Table 1). However, for nylon 11 and 12, some variations in the crystallization and melting temperatures were observed (Table 1). This finding suggest that the dissolution-regeneration process induces some changes in the crystallinity of the nylon 11 and 12.

### Behaviour during repeated dissolution-regeneration cycles–recycling of SB-IL 3 and nylon 6

As shown, SB-IL 3 is an efficient and inert solvent for a range of nylon polymers. As compared to HFIP, this SB-IL is non-volatile and thermally stable, and it has the potential to be applied in dissolution recycling of nylons. However, to achieve any practical use, the solvent should perform in repeated cycles without losing the dissolution properties due to accumulation of impurities or degradation of the solvent itself.

For the recycling trials, the SB-IL 3 and nylon 6 were selected as representative examples. In the first recycling series, virgin nylon 6 was utilized for each recycling step, where 10% (by mass) of it were dissolved and regenerated in five consecutive cycles to follow the possible changes in SB-IL 3. In the second series, successive dissolution and regeneration steps were applied for a single polymer sample with the same nylon 6 concentration of 10% (by mass).

**Chart 3:**
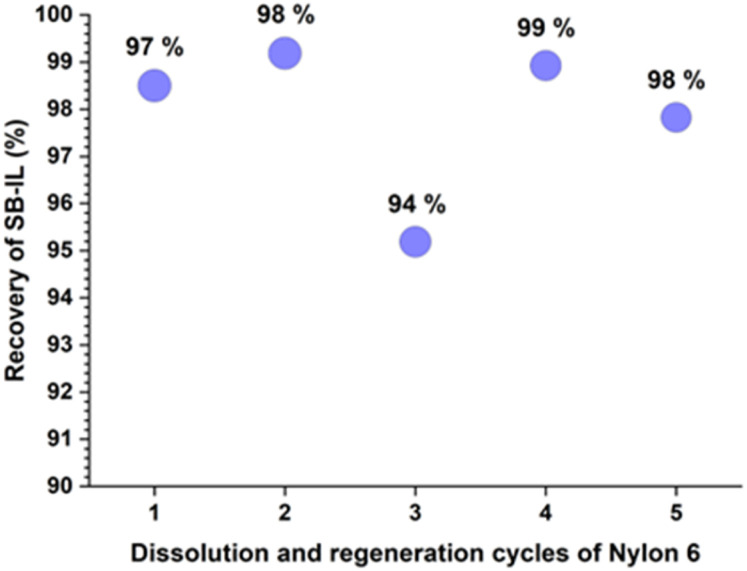
Yield of recovered SB-IL over five cycles in small scale trial. The numbers on top of the blue marks are the mass-percentage of nylon recovered at each cycle.

In the first recycling series, the continuous usage of the SB-IL 3 was probed by performing five cycles of dissolution-regeneration of nylon 6 (see details in SI, p. S43 and Table S2). Virgin nylon 6 was used for each cycle, while the SB-IL 3 (10 g scale) was recovered and reused for the next cycle. The slight loss of 3 during repeated cycles can be contributed to the aliquot samples taken for analysis and as well as to some loses during glassware transfers. The dissolution capability of the SB-IL 3 remained constant in repeated cycles (Chart 3). Further analysis of the used SB-IL 3 showed no degradation nor accumulation of low molar mass impurities (Fig. S70–S74).

In the second series, the successive dissolution-regeneration cycles for nylon 6 was repeated three times in order to see if the polymer suffers from the successive dissolution-regeneration cycles (see details in SI, p. S47 and Table S3). Each cycle consisted of the following steps: (1) virgin nylon 6 (10% (by mass)) was dissolved in 10 g SB-IL 3 or HFIP; (2) the dissolved nylon 6 was regenerated with anti-solvent (EtOH), then it was washed and dried. After that, the dissolution-regeneration cycle was repeated with the regenerated nylon 6 and concentration corrected solvent amount. Aliquot samples of the recycled nylon 6 and the SB-IL were collected for each recycling step for analysis. Thus, the isolated nylon 6 samples from this experiment were analysed with NMR, SEC, DSC and TGA, while the isolated SB-IL 3 samples were analysed by NMR (Fig. S75–S77). The NMR, FT-IR and SEC analysis of the nylon 6 samples showed no changes (Fig. S29, S30, S37, S38, S44, S45 and S78–S83). Likewise, the TGA and DSC data remained constant (Fig. S52, S53 and S60, S61). Again, there was no observable changes in the SB-IL showing no degradation or accumulation of low molar mass impurities.

Overall, the small-scale recycling experiments for nylon 6 in the SB-IL 3 showed no degradation of either the nylon material or the used SB-IL. The current results are very encouraging, as they show potential for development of efficient dissolution recycling of nylon with designed ionic liquids.

## Experimental

Description of the general and the detailed procedures can be found in the electronic supporting material associated with this article. Additionally, the used materials, methods and instrumentation are depicted in the SI.

## Conclusions

The dissolution of nylon polymers requires interfering the hydrogen bonding between the polymer chains. Three different superbase ionic liquids (SB-ILs) were tested for dissolution of nylon. Two of these were cellulose dissolving SB-ILs ([mTBNH][OAc] and [mTBDH][OAc] and the third a more hydrophobic [dm_3_-mTBDH][OAc]). While all of the tested SB-ILs show some activity for dissolution of nylons, the [dm_3_-mTBDH][OAc] demonstrated high efficiency for dissolution of different nylons at high concentrations. The recovery of dissolved nylon polymers was conducted by addition of ethanol as a non-solvent, which enables for easy and sustainable circulation of the used SB-IL.

The properties of the regenerated nylon materials were studied and compared to virgin nylon samples and those regenerated from HFIP. The nylon polymers were not altered in the dissolution-regeneration cycle as the data analysis from NMR, SEC, DSC, TGA, and FT-IR showed no degradation or impact on the dispersity or on the molar mass distribution of the material, as well as no major changes in the melting points or glass transition temperatures.

The stability and recycling results for [dm_3_-mTBDH][OAc] demonstrate that this SB-IL is stable and maintains constant dissolution efficiency through multiple dissolution/regeneration experiments. Furthermore, the consecutive recycling of the nylon 6 polymer highlights the potential of this SB-IL, as no observable changes were detected in the recycled polymer.

The current study demonstrates that recycling of nylon is likely possible with tailored dissolution of nylon polymers. This investigation also provides tools for liquid state analytics and possibly chemical modification of nylon polymers.

## Author contributions

The manuscript was written through contributions of all authors. All authors have given approval to the final version of the manuscript.

## Conflicts of interest

There are no conflicts of interest to declare.

## Supplementary Material

RA-015-D5RA07715J-s001

## Data Availability

The data supporting this article have been included as part of the supplementary information (SI) Supplementary information is available. See DOI: https://doi.org/10.1039/d5ra07715j.
