# Supplementary material for: Dissolution and regeneration of nylons utilizing superbase-acid conjugate ionic liquids
Source: RSC Adv. 2025 Dec 3;15(56):47930–7. doi: 10.1039/d5ra07715j (PMC12679557; doi:10.1039/d5ra07715j)
Supplement: RA-015-D5RA07715J-s001 [file RA-015-D5RA07715J-s001.pdf]

Supplementary Information (SI) for RSC Advances.
This journal is © The Royal Society of Chemistry 2025

Supporting information

Dissolution and Regeneration of Nylons utilizing Superbase-Acid Conjugate Ionic Liquids

Eva Gazagnaire,[a] Yuliia Bardadym,[a] Sami Hietala,[a] Sami-Pekka Hirvonen,[a] Vladimir Aseyev,[a] Ilkka Kilpeläinen*[a] and Aleksandar Todorov,*[a]

[a] Materials Chemistry Division, Department of Chemistry, University of Helsinki, FI-00560 Helsinki, Finland.

Corresponding authors: aleksandar.todorov@helsinki.fi; ilkka.kilpelainen@helsinki.fi

Contents

General information 4

General procedure for the synthesis of ionic liquid 4

General procedure for dissolution of nylons into different solvents 4

General procedure for regeneration of nylons from different solvents 4

General procedure for dissolution of nylons for NMR analysis 4

General procedure for dissolution of nylons for SEC analysis 4

Determination of water content in Ionic liquids 5

Dissolution of nylon 6

a. Specific procedures 6

b. Optical microscopy 10

Comparison of Nylon 6 in different NMR solvents 17

Nylon 6 viscosity reduction trial with different co-solvents 17

DOSY spectra comparison 18

a. DOSY measurements of virgin and regenerated nylons 18

b. Characterization of regenerated nylon from [dm_3-mTBDH][OAc] cycle 1,2,3 22

c. Characterization of regenerated nylon from HFIP cycle 1,2,3 22

Characterisation of nylons 23

a. InfraRed characterization 23

b. SEC chromatography 27

c. TGA characterization 31

d. DSC characterization 35

Characterisation of the SB-ILs for nylon dissolution by ^{1}H and ^{13}C NMR 40

Analysis of superbase ionic liquids after nylon dissolution by ^{1}H NMR 43

Recycling of superbase ionic liquid 45

Recycling of nylon 6 (three dissolution-regeneration cycles) 49

NMR spectra of nylons 55

a. Characterization of virgin nylon 6 55

b. Characterization of virgin nylon 6,6 (P) 58

c. Characterization of virgin 6,6 (M) 62

d. Characterization of virgin nylon 6,12 64

e. Characterisation of virgin nylon 11 68

f. Characterisation of virgin nylon 12 71

g. Characterisation of nylon 6 dissolved in [mTBDH][OAc] 75

h. Characterisation of nylon 6 dissolved in [dm_3-mTBDH][OAc] 77

i. Characterisation of nylon 6 in TFE 78

j. Characterisation of nylon 6,6 (P) in TFE 79

k. Characterisation of nylon 6,6 (M) in TFE 80

l. Characterisation (^{1}H) of recovered nylons from [dm_3-mTBDH][OAc] 81

m. Characterisation (^{1}H) of recovered nylons from HFIP 84

References 87

General information

All procedures, spectroscopic information and utilized equipment are collected and presented below. All commercial chemicals were used as received without further purification. Nylon materials were purchased from Sigma Aldrich: Nylon 6 pellets (product number: 181110), Nylon 6,6 (P) pellets (product number: 429171), Nylon 6,6 (M) pellets (product number: 429201), Nylon 6,12 pellets – 2 mm (product number: 181145), Nylon 11 pellets – 3 mm (product number: 181153), Nylon 12 pellets (product number: 181161). Hexafluoro-2-propanol was purchased from FluoroChem and the used superbase ionic liquids were prepared in our research group. Liquid state ^{1}H, diffusion-edited ^{1}H, ^{13}C{^{1}H}, HSQC, HSQC-TOCSY, HMBC, and DOSY NMR spectra for nylons were recorded at 40 °C or 65 °C using a Bruker Avance Neo 600 [599.69 MHz]. For the analysis of the ionic liquids Bruker Avance Neo 400 [400.15 MHz] was used at standard conditions. ^{1}H and ^{13}C{^{1}H} spectra were referenced to the residual solvent signals of DMSO-d_6 2.50 and 39.5 ppm, respectively, with no special notation for the equivalent carbons.

General procedure for the synthesis of ionic liquid

The used superbase ionic liquids were prepared in our research group following the procedure published by Gazagnaire et al.[1] A 20 mL round-bottom flask was charged with 1 molar equivalent of the corresponding superbase. Then 1 molar equivalent of acid was added, and the reaction mixture was stirred for an hour under ambient conditions. After the indicated time the ionic liquid can be directly utilized for the dissolution of nylon. The color of the prepared ionic liquid may vary from colorless to dark orange.

General procedure for dissolution of nylons into different solvents

In an 8 mL vial equipped with a cylindrical stirrer bar (10 x 6 mm), 0.1 g (10 % (by mass)) of nylon and 0.9 g of solvent (SB-IL or HFIP or TFE) were charged and the vial was sealed. Then the suspended nylon sample was immersed into a preheated silicon oil bath (100 °C) for 20 hours. After that, the samples were analysed by optical microscopy, or they were regenerated (see section general procedure for regeneration of nylons from different solvents).

General procedure for regeneration of nylons from different solvents

To the hot nylon solution was added 7 mL ethanol (EtOH) in order to avoid gelation of the solutions. Once the nylon has started to precipitate the mixture was transferred to 150 mL of ethanol for its full precipitation. After one hour at room temperature the formed solids were filtered over a sintered funnel (Pore size 4) and were transferred back into a fresh EtOH (150 mL). This step was repeated in total 3 times to wash out as much as possible of the used nylon solvent. After this, the nylon sample was dried under high vacuum at room temperature over 20 hours.

General procedure for dissolution of nylons for NMR analysis

Typically, nylon sample 0.025 g was placed into 4 mL vial, followed by a cylindrical stirrer bar (10 x 6 mm) and 1 g of HFIP (2.5 % (by mass)). Then the nylon sample was stirred until complete dissolution. After that, the nylon solution was transferred to an NMR tube, where a sealed capillary containing DMSO-d_6 was added and the sample was analysed by NMR.

General procedure for dissolution of nylons for SEC analysis

Samples for size exclusion chromatography (SEC) were prepared by weighing 1 mg of nylon in a 4 ml glass vial with a PTFE sealing cap followed by addition of 1.0 ml of pure HFIP. Nylons were left to dissolve overnight at room temperature and the samples were visually inspected for complete dissolution. All samples were filtered through 0.22 μm PTFE syringe filters (Clarify) prior to the SEC measurements.

Determination of water content in Ionic liquids

The water content of the ionic liquids has been determined by coulometric Karl-Fischer titration using the C10S Mettler Toledo coulometric titrator. To mitigate the differences in the physical states (solid or viscous liquids), the SB-IL samples were measured as a DMSO solutions with known concentrations. The amount of water in each sample was determined utilizing the following equation:

$$H_2O_{(total)} = H_2O_{(SB\text{-}IL\text{-}m)} + H_2O_{(DMSO\text{-}m)}$$

from there,

$$H_2O_{(SB\text{-}IL\text{-}m)} = H_2O_{(total)} - H_2O_{(DMSO\text{-}m)}$$

where, $H_2O_{(total)}$ is the water measured in the SB-IL/DMSO mixture and $H_2O_{(DMSO\text{-}m)}$ is the water measured for the DMSO in the mixture. To find the $H_2O_{(DMSO\text{-}m)}$ we can use the following equation:

$$H_2O_{(DMSO\text{-}m)} = (H_2O_{(DMSO\text{-}p)} / m_{(injected\ DMSO\text{-}p)}) * m\text{-}fr._{(DMSO\ for\ the\ mixture)}$$

where, $H_2O_{(DMSO\text{-}p)}$ is the water amount in pure DMSO (in grams), $m_{(injected\ DMSO\text{-}p)}$ is the mass of injected DMSO for the water measurement, and $m\text{-}fr._{(DMSO\ for\ the\ mixture)}$ is the mass fraction of DMSO present in the injected SB-IL/DMSO mixture. The last term can be calculated easily from the following equation:

$$m\text{-}fr._{(DMSO\ for\ the\ mixture)} = (m_{(injected\ SB\text{-}IL/DMSO\ mixture)} / m_{(prepared\ SB\text{-}IL/DSMO\ sample)}) * m_{(DMSO\ used\ for\ the\ SB\text{-}IL/DMSO\ mixture)}$$

where, $m_{(injected\ SB\text{-}IL/DMSO\ mixture)}$ is the mass of injected SB-IL/DMSO mixture for titration (in grams), $m_{(prepared\ SB\text{-}IL/DSMO\ sample)}$ is the total mass of the prepared SB-IL/DMSO mixture (in grams), and $m_{(DMSO\ used\ for\ the\ SB\text{-}IL/DMSO\ mixture)}$ is the mass of the used DMSO for the preparation of the SB-IL/DMSO mixture.

For [mTBNH][OAc] a mixture of 0.5205 g [mTBNH][OAc] and 0.5053 g DMSO was prepared. The titration was first performed on a pure DMSO sample (0.6347 g), where the water content in the DMSO was determined to be 0.09 % (by mass) (881.24 ppm or 0.000559 g). Then the titration was performed on the [mTBNH][OAc]/DMSO mixture (0.9671 g). The total amount of water for the injected mixture was determined to be 0.20 % (by mass) (1981.35 ppm or 0.001916 g). From there in the [mTBNH][OAc] fraction the water content was determined to be 0.30 % (by mass) (3049.33 ppm or 0.001496 g).

For [mTBDH][OAc] a mixture of 0.5168 g [mTBDH][OAc] and 0.5052 g DMSO was prepared. The titration was first performed on a pure DMSO sample (0.5686 g), where the water content in the DMSO was determined to be 0.09 % (by mass) (921.47 ppm or 0.000524). Then the titration was performed on the [mTBDH][OAc]/DMSO mixture (0.9640 g). The total amount of water for the injected mixture was determined to be 0.26 % (by mass) (2618.68 ppm or 0.002524 g). From there in the [mTBDH][OAc] fraction the water content was determined to be 0.43 % (by mass) (4277.79 ppm or 0.002085 g).

For [dm$_3$-mTBDH][OAc] a mixture of 0.5211 g [dm$_3$-mTBDH][OAc] and 0.5046 g DMSO was prepared. The titration was first performed on a pure DMSO sample (0.5349 g), where the water content in the DMSO was determined to be 0.09 % (by mass) (923.53 ppm or 0.000494 g). Then the titration was performed on the [dm$_3$-mTBDH][OAc]/DMSO mixture (0.9646 g). The total amount of water for the injected mixture was determined to be 0.38 % (by mass) (3752.22 ppm or 0.003619 g). From there in the [dm$_3$-mTBDH][OAc] fraction the water content was determined to be 0.65 % (by mass) (6491.34 ppm or 0.003181 g).

Dissolution of nylon

a. Specific procedures

Dissolution-regeneration of Nylon 6: The regenerated material was obtained following the *general procedures for dissolution and regeneration*.

Starting from Nylon 6 0.50 g and 5.00 g of [dm_3-mTBDH][OAc]. Dissolution time was 20 h and the temperature was set at 100 °C. Obtained: 0.47 g, light yellow powder.

Starting from Nylon 6 0.50 g and 5.00 g of HFIP. Dissolution time was 20 h and the temperature was set at 40 °C. Obtained: 0.50 g, white powder.

NMR data for virgin Nylon 6 in different solvents:

HFIP/DMSO:

^{1}H NMR (600 MHz, DMSO/HFIP) δ 5.77 (s, 1H), 2.85 (q, *J* = 6.8 Hz, 2H), 1.84 (t, *J* = 7.9 Hz, 2H), 1.24 (h, *J* = 8.1 Hz, 2H), 1.16 (p, *J* = 7.6 Hz, 2H), 0.97 (hept, *J* = 8.5 Hz, 2H).

^{13}C NMR (151 MHz, DMSO/HFIP) δ 176.1, 119.2, 68.4, 39.5, 38.6, 34.9, 26.8, 24.5, 23.8.

TFE/DMSO:

^{1}H NMR (600 MHz, DMSO/TFE) δ 6.48 (s, 1H), 2.93 (q, *J* = 6.4 Hz, 2H), 1.96 – 1.90 (m, 2H), 1.34 (dt, *J* = 15.1, 7.7 Hz, 2H), 1.25 (p, *J* = 7.8 Hz, 2H), 1.07 (td, *J* = 8.6, 4.3 Hz, 2H).

NMR data for regenerated Nylon 6 from [dm_3-mTBDH][OAc] in HFIP/DMSO:

^{1}H NMR (600 MHz, DMSO/HFIP) δ 5.78 (s, 1H), 2.85 (d, *J* = 7.8 Hz, 2H), 1.84 (d, *J* = 7.9 Hz, 2H), 1.24 (p, *J* = 8.2 Hz, 2H), 1.16 (t, *J* = 7.6 Hz, 2H), 0.98 (d, *J* = 9.0 Hz, 2H).

NMR data for regenerated Nylon 6 from HFIP in HFIP/DMSO:

^{1}H NMR (600 MHz, DMSO) δ 5.78 (s, 1H), 2.85 (d, *J* = 6.8 Hz, 2H), 1.85 (t, *J* = 7.8 Hz, 2H), 1.24 (p, *J* = 8.2 Hz, 2H), 1.16 (p, *J* = 7.9 Hz, 2H), 0.98 (s, 2H).

Dissolution-regeneration of Nylon 6,6 (P): The regenerated material was obtained following the *general procedures for dissolution and regeneration*.

Starting from Nylon 6,6 (P) 0.50 g and 5.00 g of [dm_3-mTBDH][OAc]. Dissolution time was 20 h and the temperature was set at 100 °C. Obtained: 0.43 g, white powder.

Starting from Nylon 6,6 (P) 0.50 g and 5.00 g of HFIP. Dissolution time was 20 h and the temperature was set at 40 °C. Obtained: 0.50 g, white powder.

NMR data for virgin Nylon 6,6 (P) in different solvents:

HFIP/DMSO:

^{1}H NMR (600 MHz, DMSO/HFIP) δ 6.20 (s, 1H), 3.13 (q, *J* = 6.8 Hz, 2H), 2.14 (d, *J* = 6.8 Hz, 2H), 1.53 (p, *J* = 3.6 Hz, 2H), 1.43 (t, *J* = 7.2 Hz, 2H), 1.25 (q, *J* = 3.6 Hz, 2H).

^{13}C NMR (151 MHz, DMSO/HFIP) δ 176.2, 119.8, 69.0, 40.1, 39.5, 35.1, 27.7, 25.2, 24.2.

TFE/DMSO:

^{1}H NMR (600 MHz, DMSO/TFE) δ 6.49 (s, 1H), 2.92 (q, *J* = 6.7 Hz, 2H), 1.93 (d, *J* = 6.5 Hz, 2H), 1.34 (h, *J* = 4.9 Hz, 2H), 1.24 (p, *J* = 7.0 Hz, 2H), 1.11 – 1.01 (m, 2H).

NMR data for regenerated Nylon 6,6 (P) from [dm_3-mTBDH][OAc] in HFIP/DMSO:

^{1}H NMR (600 MHz, DMSO/HFIP) δ 5.82 (s, 1H), 2.85 (s, 2H), 1.86 (s, 2H), 1.24 (s, 2H), 1.15 (s, 2H), 0.97 (s, 2H).

NMR data for regenerated Nylon 6,6 (P) from HFIP in HFIP/DMSO:

^{1}H NMR (600 MHz, DMSO/HFIP) δ 5.82 (s, 1H), 2.86 (s, 2H), 1.87 (s, 2H), 1.25 (s, 2H), 1.16 (s, 2H), 0.98 (s, 2H).

Dissolution-regeneration of Nylon 6,6 (M): The regenerated material was obtained following the *general procedures for dissolution and regeneration*.

Starting from Nylon 6,6 (M) 0.50 g and 5.00 g of [dm_3-mTBDH][OAc]. Dissolution time was 20 h and the temperature was set at 100 °C. Obtained: 0.43 g, white powder.

Starting from Nylon 6,6 (M) 0.50 g and 5.00 g of HFIP. Dissolution time was 20 h and the temperature was set at 40 °C. Obtained: 0.50 g, white powder.

NMR data for virgin Nylon 6,6 (M) in different solvents:

HFIP/DMSO:

^{1}H NMR (600 MHz, DMSO/HFIP) δ 5.82 (s, 1H), 2.86 (q, *J* = 6.8 Hz, 2H), 1.87 (d, *J* = 6.4 Hz, 2H), 1.29 – 1.22 (m, 2H), 1.15 (q, *J* = 7.0 Hz, 2H), 1.00 – 0.95 (m, 2H).

TFE/DMSO:

^{1}H NMR (600 MHz, DMSO/TFE) δ 6.48 (s, 1H), 2.93 (d, *J* = 6.8 Hz, 2H), 1.95 (s, 2H), 1.35 (s, 2H), 1.30 – 1.19 (m, 2H), 1.08 (s, 2H).

NMR data for regenerated Nylon 6,6 (M) from [dm_3-mTBDH][OAc] in HFIP/DMSO:

^{1}H NMR (600 MHz, DMSO/HFIP) δ 5.83 (s, 1H), 2.87 (s, 2H), 1.88 (s, 2H), 1.24 (s, 2H), 1.18 (s, 2H), 0.99 (s, 2H).

NMR data for regenerated Nylon 6,6 (M) from HFIP in HFIP/DMSO:

^{1}H NMR (600 MHz, DMSO/HFIP) δ 5.80 (s, 1H), 2.84 (q, *J* = 6.6 Hz, 2H), 1.86 (d, *J* = 6.6 Hz, 2H), 1.24 (p, *J* = 3.5 Hz, 2H), 1.14 (t, *J* = 7.2 Hz, 2H), 1.01 – 0.92 (m, 2H).

Dissolution-regeneration of Nylon 6,12: The regenerated material was obtained following the *general procedures for dissolution and regeneration*.

Starting from Nylon 6,12 0.50g and 5.00 g of [dm_3-mTBDH][OAc]. Dissolution time was 20 h and the temperature was set at 100 °C. Obtained: 0.42 g, light yellow powder.

Starting from Nylon 6,12 0.50 g and 5.00 g of HFIP. Dissolution time was 20 h and the temperature was set at 40 °C. Obtained: 0.50 g, white powder.

NMR data for virgin Nylon 6,12 in HFIP/DMSO:

^{1}H NMR (600 MHz, DMSO/HFIP) δ 5.72 (s, 2H), 2.87 (q, *J* = 6.8 Hz, 4H), 1.85 (t, *J* = 7.9 Hz, 4H), 1.22 (t, *J* = 7.4 Hz, 4H), 1.16 (d, *J* = 7.1 Hz, 4H), 1.05 – 0.82 (m, 16H).

^{13}C NMR (151 MHz, DMSO/HFIP) δ 177.4, 123.5, 121.7, 119.8, 117.9, 69.0, 40.1, 39.4, 35.8, 28.2, 28.0, 28.0, 27.7, 25.2, 24.9.

NMR data for regenerated Nylon 6,12 from [dm_3-mTBDH][OAc] in HFIP/DMSO:

^{1}H NMR (600 MHz, DMSO/HFIP) δ 5.71 (s, 2H), 2.86 (s, 4H), 1.85 (s, 4H), 1.21 (s, 4H), 1.16 (s, 4H), 0.95 (s, 16H).

NMR data for regenerated Nylon 6,12 from HFIP in HFIP/DMSO:

^{1}H NMR (600 MHz, DMSO/HFIP) δ 5.72 (s, 2H), 2.87 (q, *J* = 6.8 Hz, 4H), 1.86 (t, *J* = 7.9 Hz, 4H), 1.21 (q, *J* = 7.5 Hz, 4H), 1.16 (q, *J* = 7.0 Hz, 4H), 1.03 – 0.92 (m, 16H).

Dissolution-regeneration of Nylon 11: The regenerated material was obtained following the *general procedures for dissolution and regeneration*.

Starting from Nylon 11 0.50 g and 5.00 g of [dm_3-mTBDH][OAc]. Dissolution time was 20 h and the temperature was set at 100 °C. Obtained: 0.41 g, light yellow powder.

Starting from Nylon 11 0.50 g and 5.00 g of HFIP. Dissolution time was 20 h and the temperature was set at 40 °C. Obtained: 0.50 g, white powder.

NMR data for virgin Nylon 11 in HFIP/DMSO:

^{1}H NMR (600 MHz, DMSO/HFIP) δ 3.12 (q, *J* = 6.8 Hz, 2H), 2.11 (t, *J* = 7.8 Hz, 2H), 1.40 (s, 2H), 1.27 – 1.15 (m, 12H).

^{13}C NMR (151 MHz, DMSO) δ 177.3, 123.5, 121.7, 119.8, 117.9, 69.0, 40.1, 39.6, 35.8, 28.2, 28.2, 28.0, 28.0, 27.8, 25.6, 24.9.

NMR data for regenerated Nylon 11 from [dm_3-mTBDH][OAc] in HFIP/DMSO:

^{1}H NMR (600 MHz, DMSO/HFIP) δ 5.67 (s, 1H), 2.86 (s, 2H), 1.85 (t, *J* = 7.9 Hz, 2H), 1.22 (s, 2H), 1.15 (s, 2H), 0.95 (s, 12H).

NMR data for regenerated Nylon 11 from HFIP in HFIP/DMSO:

^{1}H NMR (600 MHz, DMSO/HFIP) δ 5.67 (s, 1H), 2.85 (q, *J* = 6.8 Hz, 2H), 1.84 (t, *J* = 7.8 Hz, 2H), 1.20 (q, *J* = 7.3 Hz, 2H), 1.14 (t, *J* = 7.0 Hz, 2H), 0.98 – 0.92 (m, 12H).

Dissolution-regeneration of Nylon 12: The regenerated material was obtained following the *general procedures for dissolution and regeneration*.

Starting from Nylon 12 0.50 g and 5.00 g of [dm_3-mTBDH][OAc]. Dissolution time was 20 h and the temperature was set at 100 °C. Obtained: 0.40 g, white powder.

Starting from Nylon 12 0.50 g and 5.00 g of HFIP. Dissolution time was 20 h and the temperature was set at 40 °C. Obtained: 0.50 g, white powder.

NMR data for virgin Nylon 12 in HFIP/DMSO:

^{1}H NMR (600 MHz, DMSO/HFIP) δ 5.68 (s, 1H), 2.84 (q, *J* = 6.5 Hz, 2H), 1.83 (t, *J* = 8.0 Hz, 2H), 1.20 (p, *J* = 7.2 Hz, 2H), 1.13 (q, *J* = 6.9 Hz, 2H), 1.00 – 0.85 (m, 14H).

^{13}C NMR (151 MHz, DMSO/HFIP) δ 177.3, 123.5, 121.7, 119.8, 117.9, 69.0, 40.1, 39.6, 35.8, 28.3, 28.3, 28.3, 28.1, 28.0, 27.8, 25.6, 24.9.

NMR data for regenerated Nylon 12 from [dm_3-mTBDH][OAc] in HFIP/DMSO:

^{1}H NMR (600 MHz, DMSO/HFIP) δ 5.69 (s, 1H), 2.88 (s, 2H), 1.87 (s, 2H), 1.24 (s, 2H), 1.17 (s, 2H), 0.97 (s, 14H).

NMR data for regenerated Nylon 12 from HFIP in HFIP/DMSO:

^{1}H NMR (600 MHz, DMSO/HFIP) δ 5.66 (s, 1H), 2.85 (q, *J* = 6.8 Hz, 2H), 1.84 (t, *J* = 7.8 Hz, 2H), 1.20 (d, *J* = 7.8 Hz, 2H), 1.14 (t, *J* = 7.0 Hz, 2H), 0.98 – 0.91 (m, 14H).

b. Optical microscopy

Each nylon dissolution trial was studied with an optical microscope (Olympus BX51TF microscope, equipped with DP70 colour camera and adjustable polarized lenses). The Cross-polarizer angle was optimized for better contrast between the solvent and the remaining nylon particles (if any).

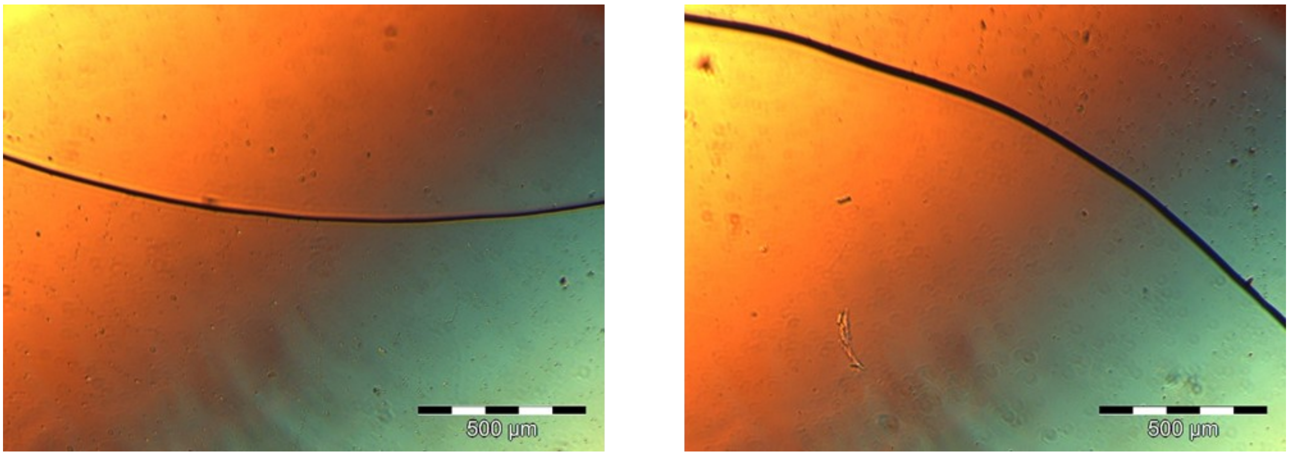

Figure S1. Microscopy images of nylon 6 dissolution in [dm$_3$-mTBDH][OAc].

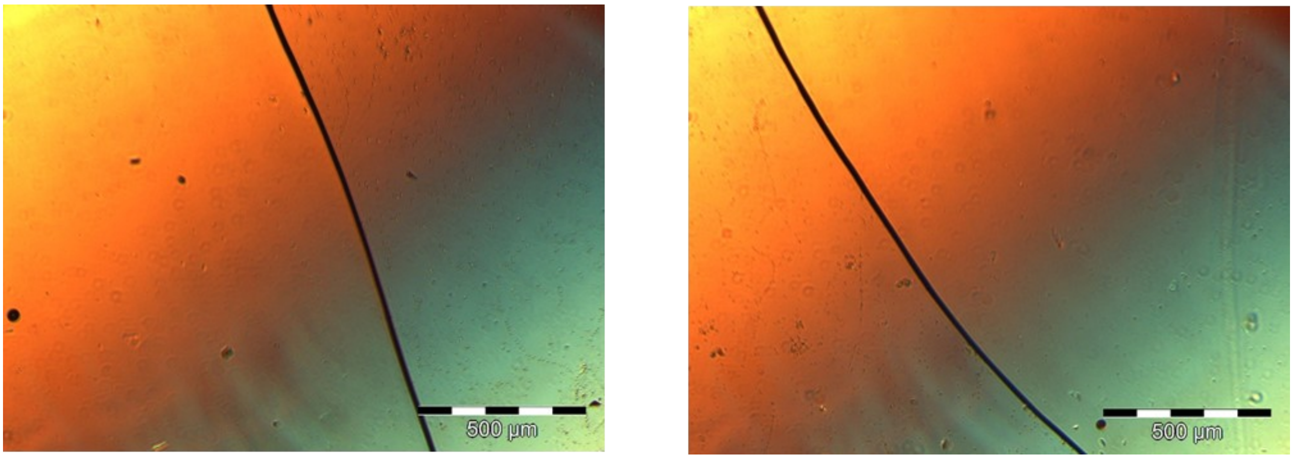

Figure S2. Microscopy images of nylon 6,6 (P) dissolution in [dm$_3$-mTBDH][OAc].

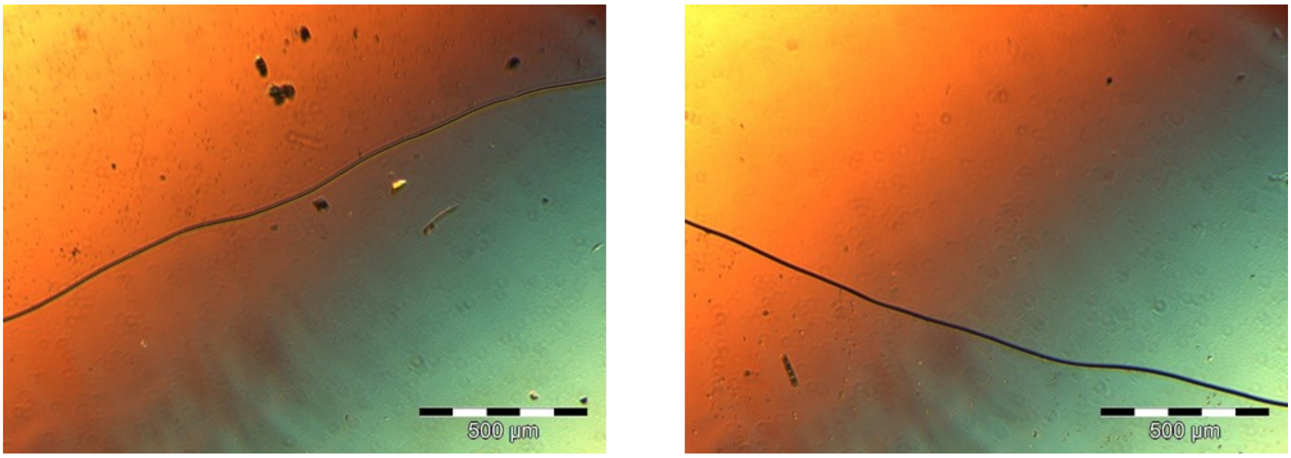

Figure S3. Microscopy images of nylon 6,6 (M) dissolution in [dm$_3$-mTBDH][OAc].

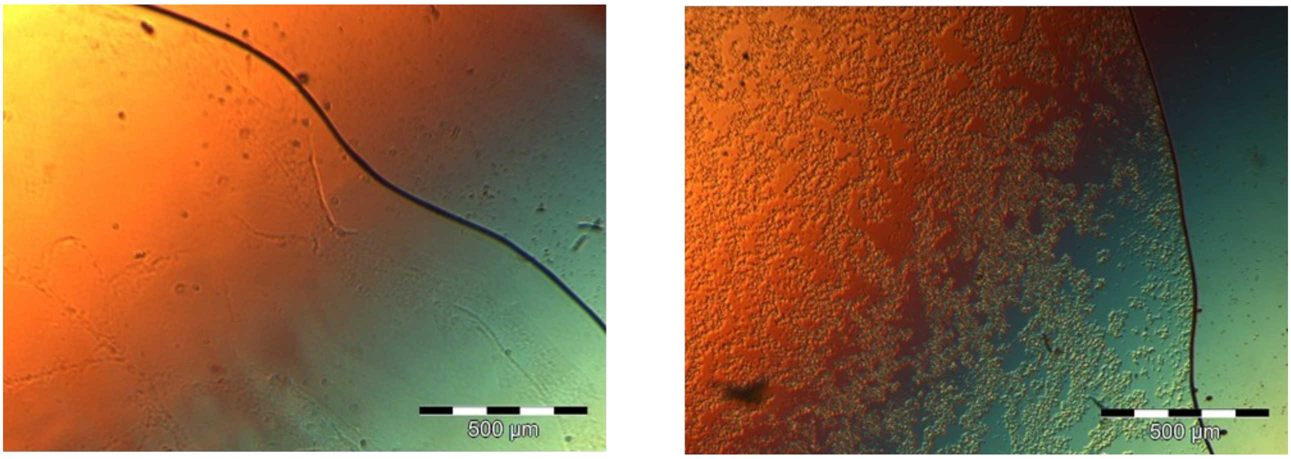

Figure S4. Microscopy images of nylon 6,12 dissolution in [dm$_3$-mTBDH][OAc]. The left picture was taken after heating the sample to 60°C and the right picture was taken at RT

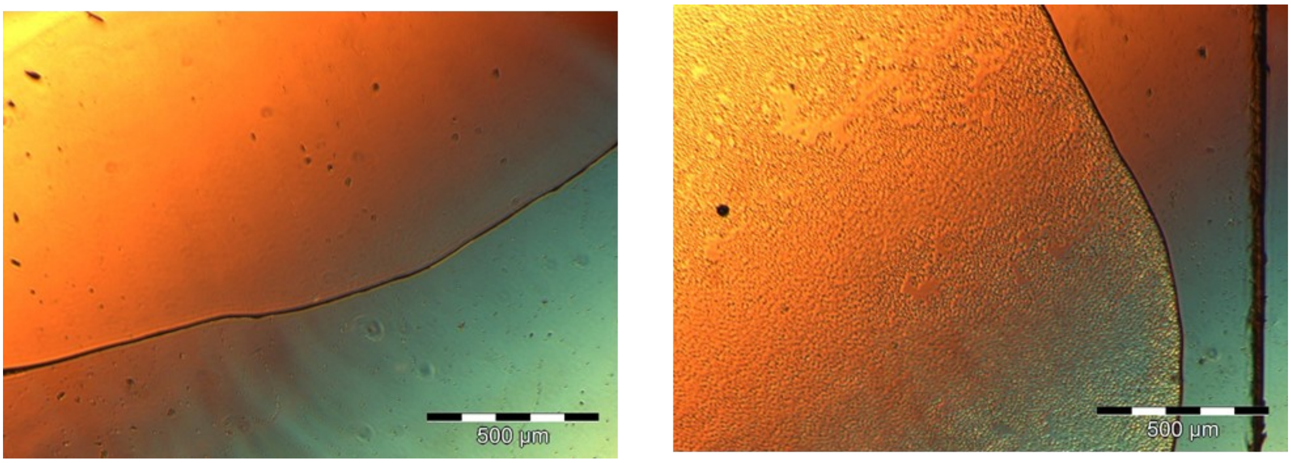

Figure S5. Microscopy images of nylon 11 dissolution in [dm$_3$-mTBDH][OAc]. The left picture was taken after heating the sample to 60°C and the right picture was taken at RT.

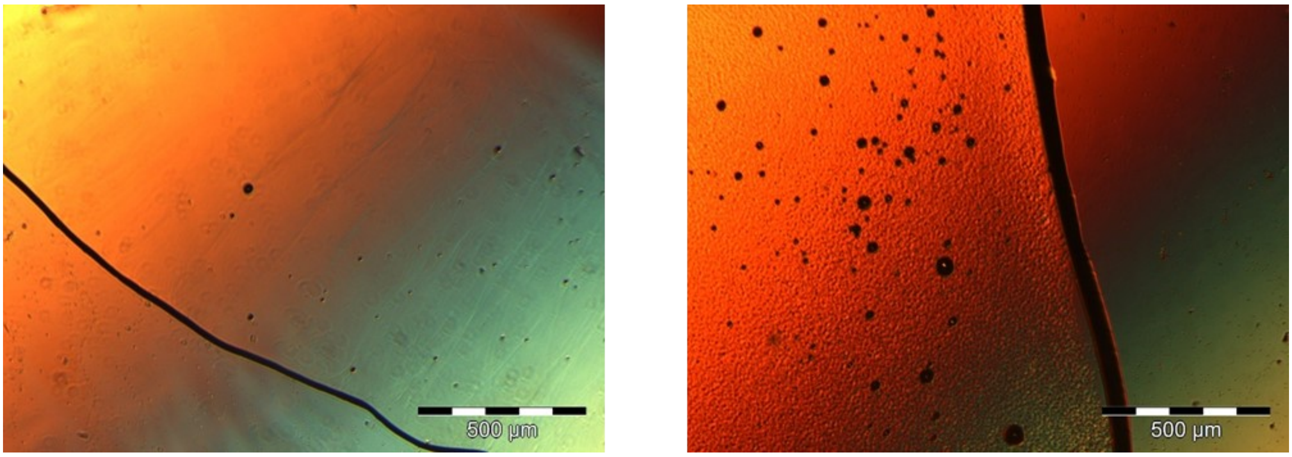

Figure S6. Microscopy images of nylon 12 dissolution in [dm$_3$-mTBDH][OAc]. The left picture was taken after heating the sample to 60°C and the right picture was taken at RT

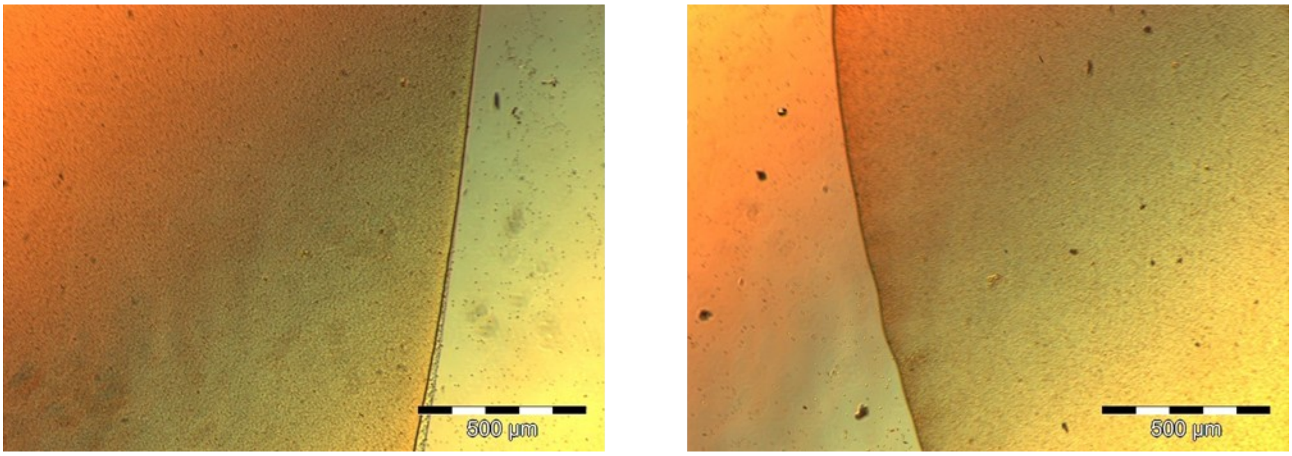

Figure S7. Microscopy images of nylon 6 dissolution in HFIP. The pictures were taken at RT

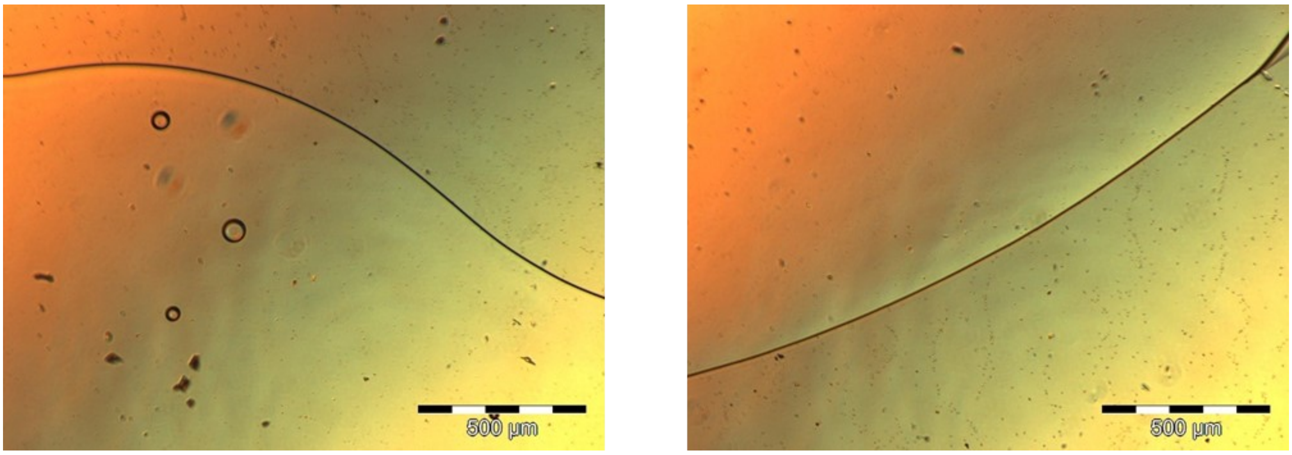

Figure S8. Microscopy images of nylon 6,6 (P) dissolution in HFIP. The pictures were taken at RT.

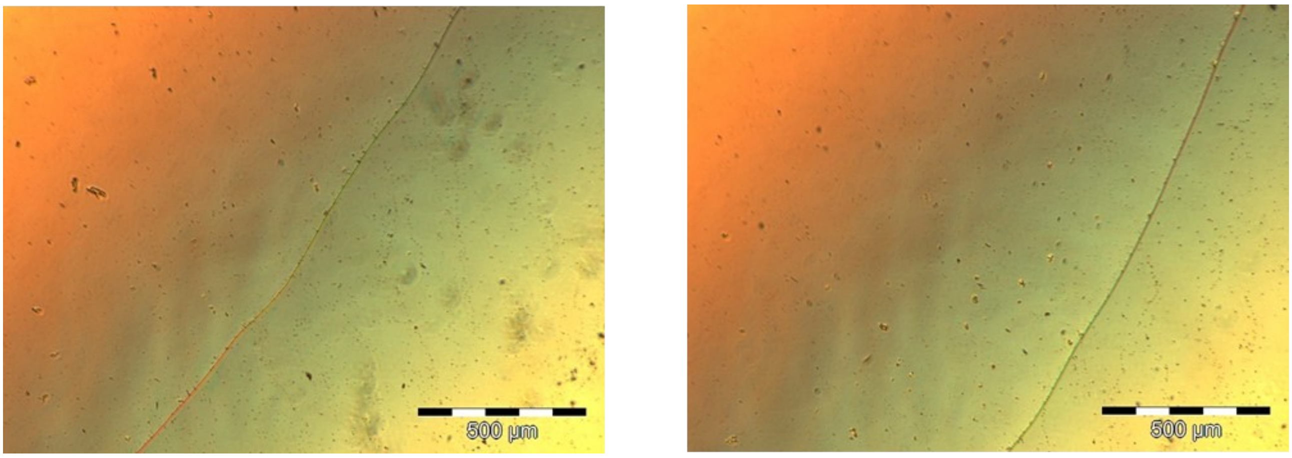

Figure S9. Microscopy images of nylon 6,6 (M) dissolution in HFIP. The pictures were taken at RT.

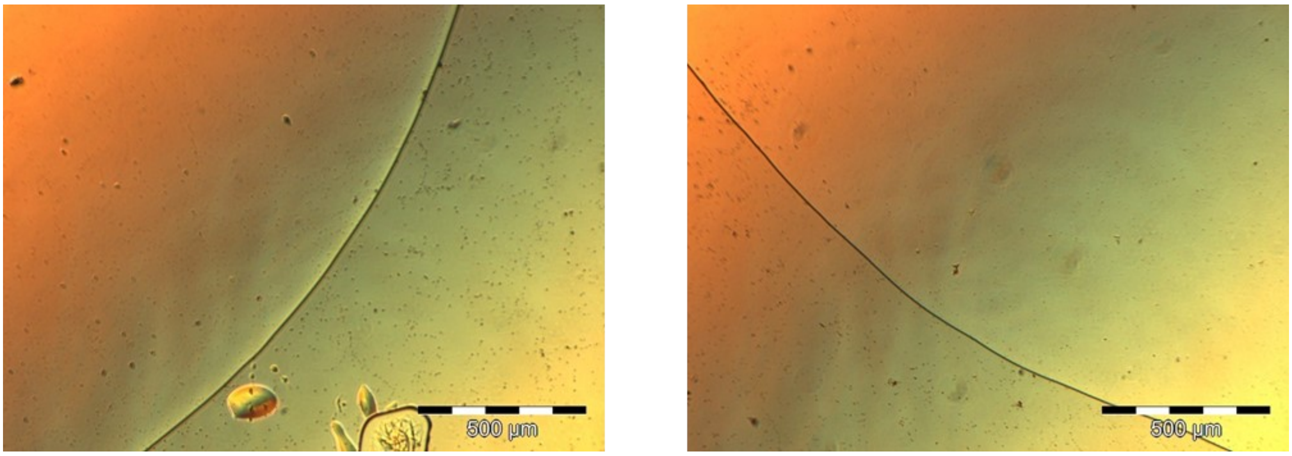

Figure S10. Microscopy images of nylon 11 dissolution in HFIP. The pictures were taken at RT.

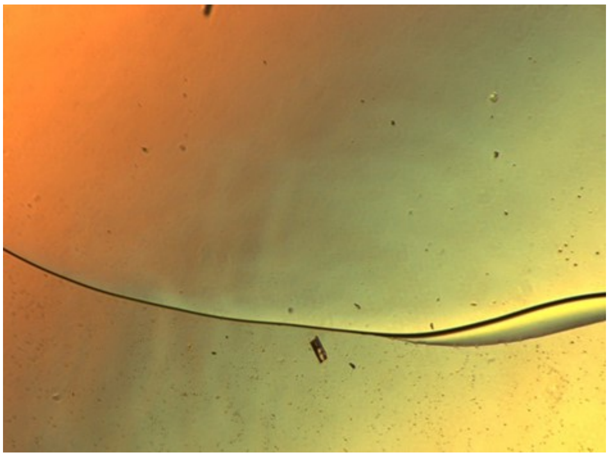

Figure S11. Microscopy images of nylon 12 dissolution in HFIP. The pictures were taken at RT.

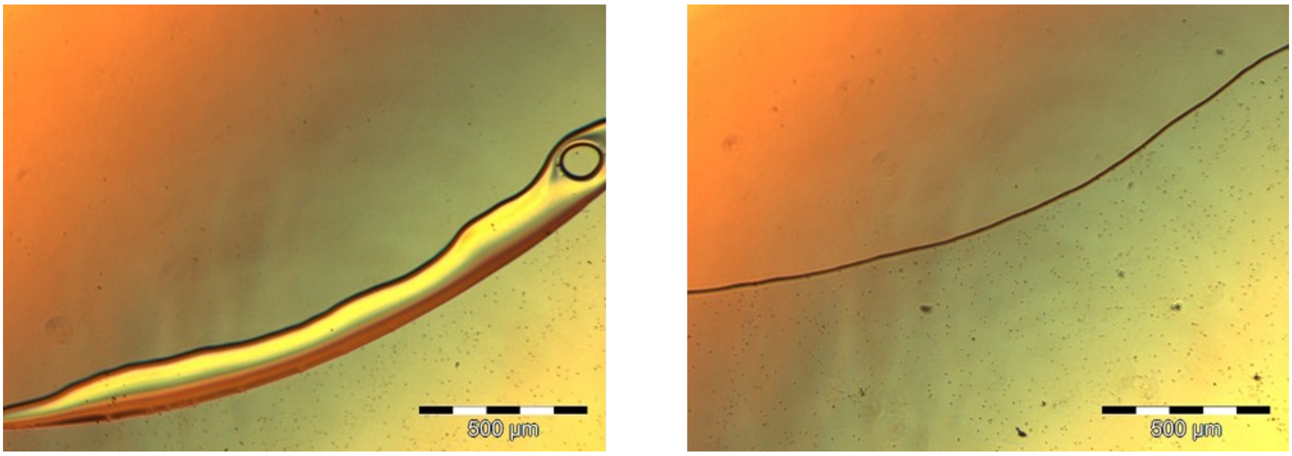

Figure S12. Microscopy images of nylon 6 dissolution in [mTBDH][OAc]. The pictures were taken at RT.

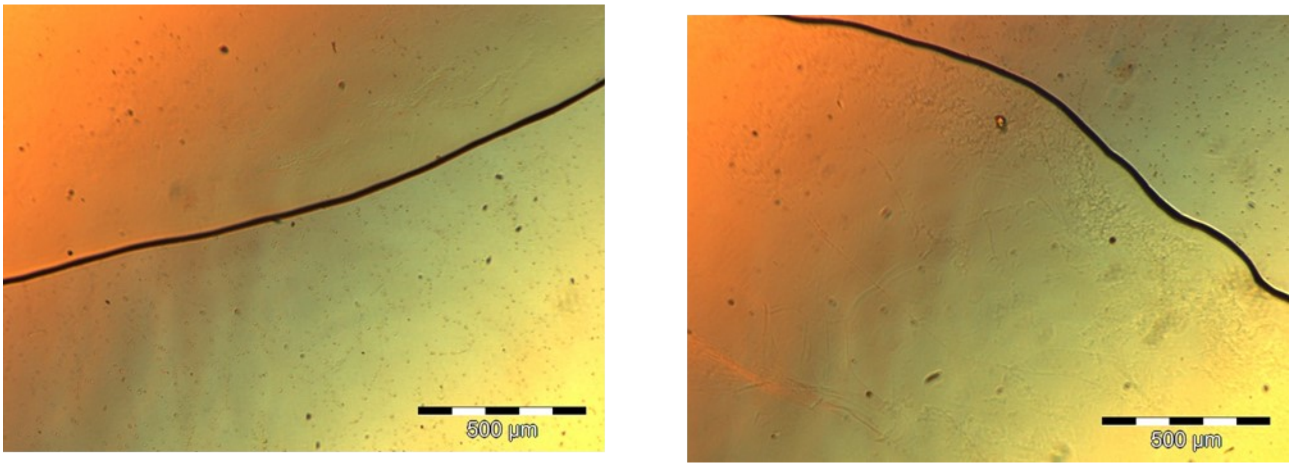

Figure S13. Microscopy images of nylon 6,6 (P) dissolution in [mTBDH][OAc]. The pictures were taken at RT.

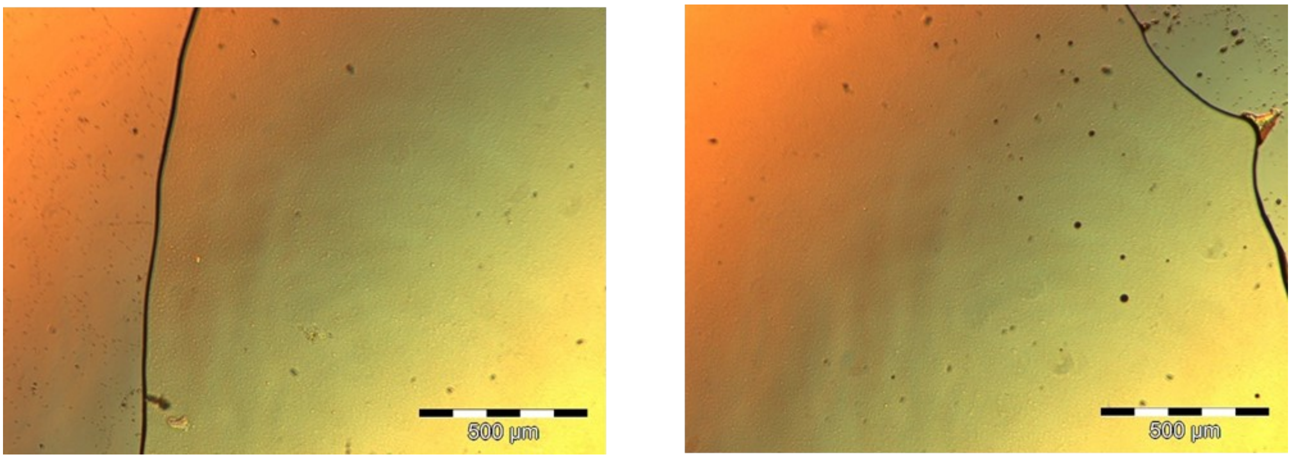

Figure S14. Microscopy images of nylon 6,6 (M) dissolution in [mTBDH][OAc]. The pictures were taken at RT.

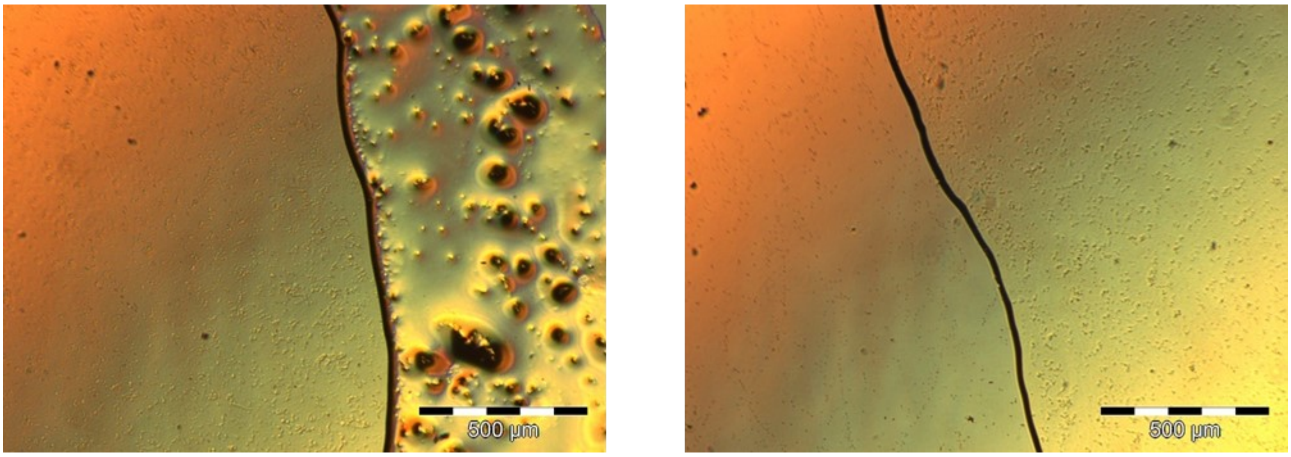

Figure S15. Microscopy images of nylon 6,12 dissolution in [mTBDH][OAc]. The pictures were taken at RT.

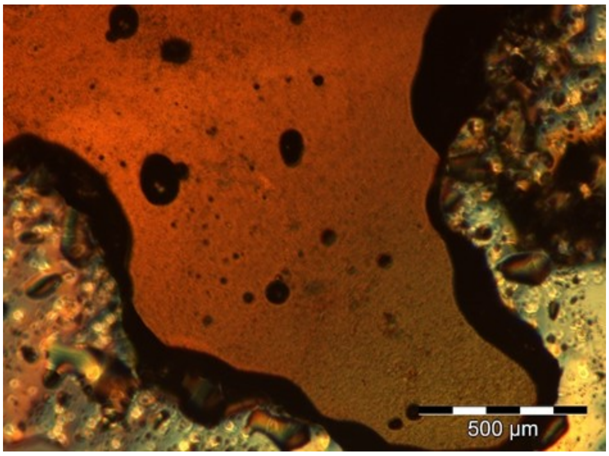

Figure S16. Microscopy images of nylon 12 dissolution in [mTBDH][OAc]. The picture was taken at RT.

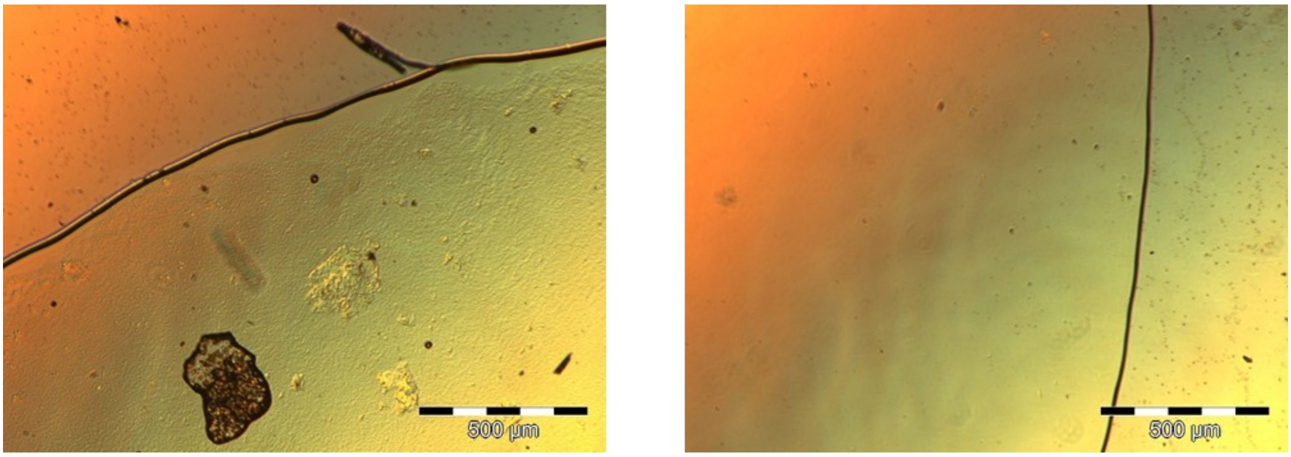

Figure S17. Microscopy images of nylon 6 dissolution in [mTBNH][OAc]. The left picture was taken after heating the sample to 60°C and the right picture was taken at RT.

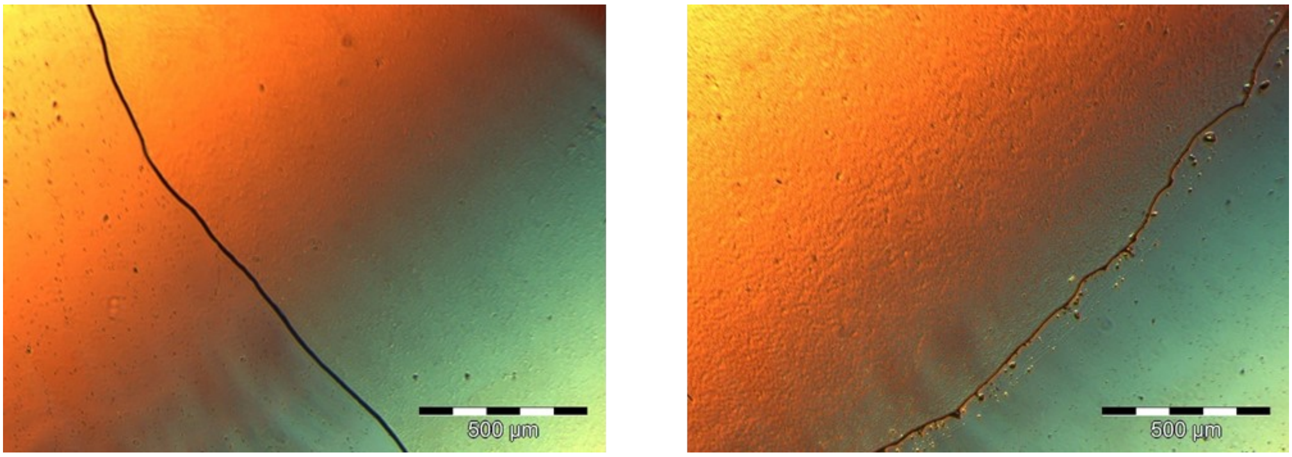

Figure S18. Microscopy images of nylon 6,6 (P) dissolution in [mTBNH][OAc]. The left picture was taken after heating the sample to 60°C and the right picture was taken at RT.

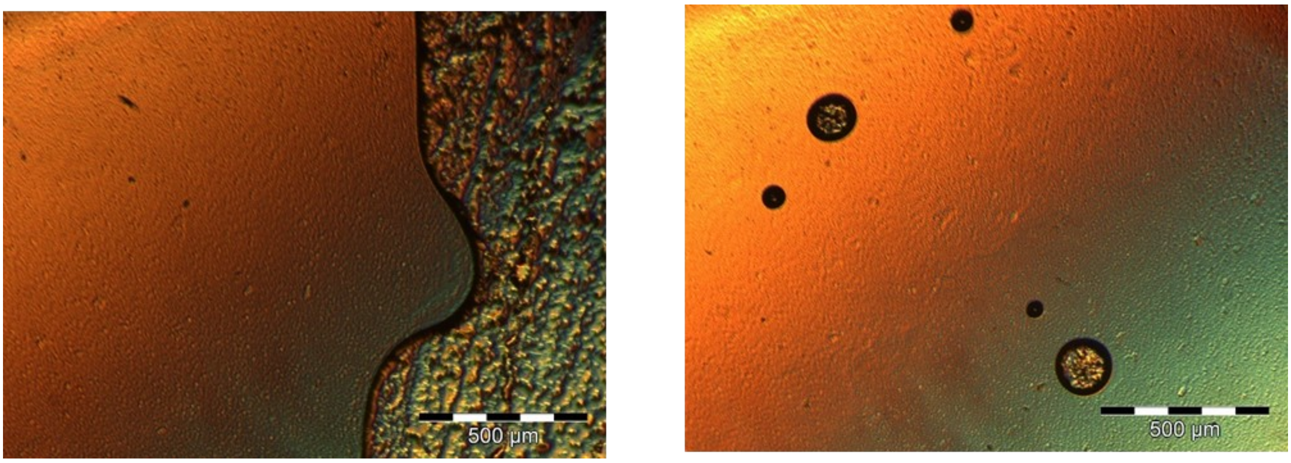

Figure S19. Microscopy images of nylon 11 dissolution in [mTBNH][OAc]. The pictures were taken at RT.

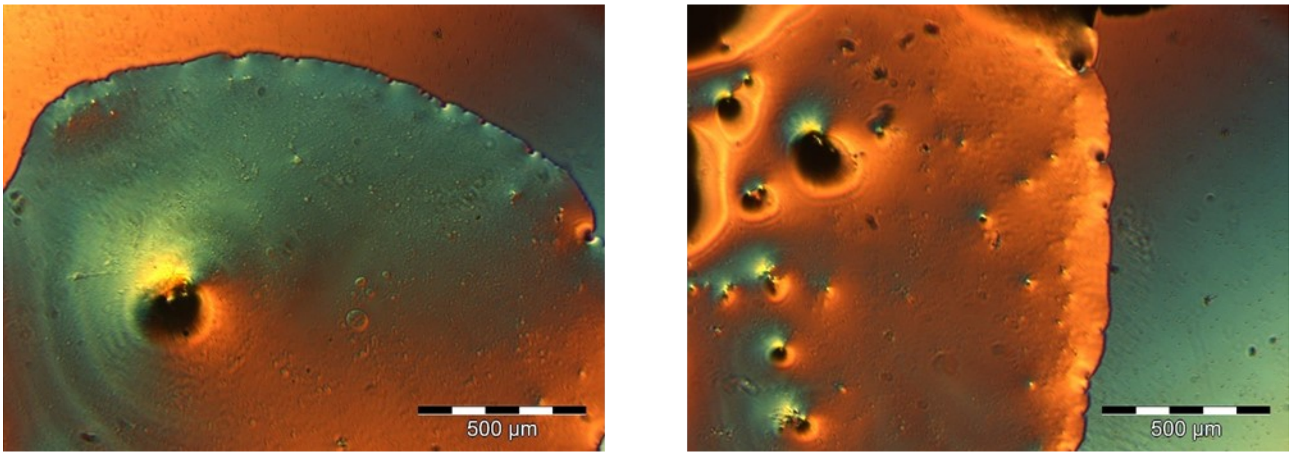

Figure S20. Microscopy images of nylon 11 dissolution in [mTBNH][OAc]. The pictures were taken at RT.

Comparison of Nylon 6 in different NMR solvents

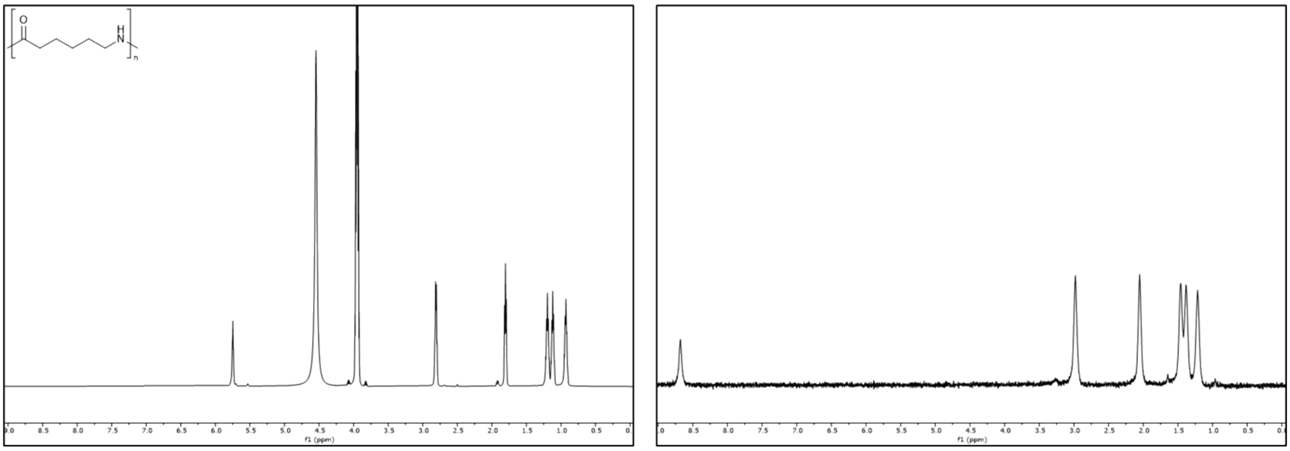

Figure S21 ^{1}H NMR of Nylon 6 in HFIP compared to diffusion edited ^{1}H NMR in [dm$_3$-mTBDH][OAc]

Nylon 6 viscosity reduction trial with different co-solvents

To reduce the viscosity of nylon 6 dissolved in [dm$_3$-mTBDH][OAc], different co-solvents were tried. For this experiment aliquots (0.5g) of nylon 6 (10 % (by mass)) were transferred to separate vial where equal amount of co-solvent was added. The following co-solvents: dichloromethane (DCM), chloroform ($CDCl_3$), acetone, acetonitrile (MeCN), dioxane, tetrahydrofuran (THF), gamma-valerolactone (g-VL), dimethylformamide (DMF), dimethylacetamide (DMA), dimethyl sulfoxide (DMSO) and water (H_2O) were tested (figure S79).

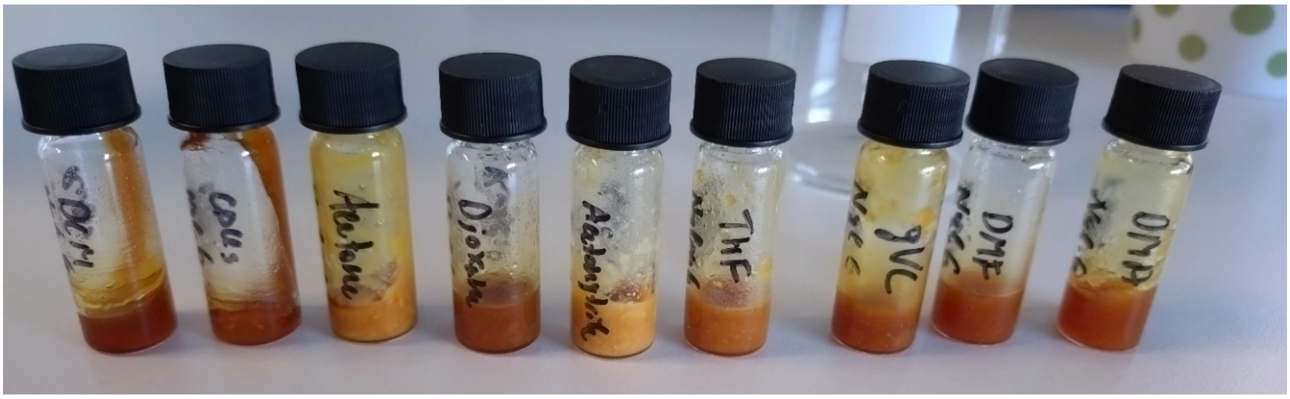

However, no suitable co-solvent was found.

Figure S22. Viscosity reduction trials with different co-solvents

DOSY spectra comparison

2D DOSY experiments for nylons were performed at 40 °C. The nylons were dissolved in HFIP at 2.5 % (by mass) and were analysed as such. The DMSO solvent for locking and tunning the NMR spectrometer was introduced in a sealed capillary to the nylon solutions. The following parameters were utilised to record the 2D DOSY experiments: number of scans - 16; repetitions - 32; relaxation delay - 3; diffusion gradient strength GPZ6 - linear gradient from 5 to 98 %; diffusion delay D20 – 0.800; gradient length P30 – 2500.

a. DOSY measurements of virgin and regenerated nylons

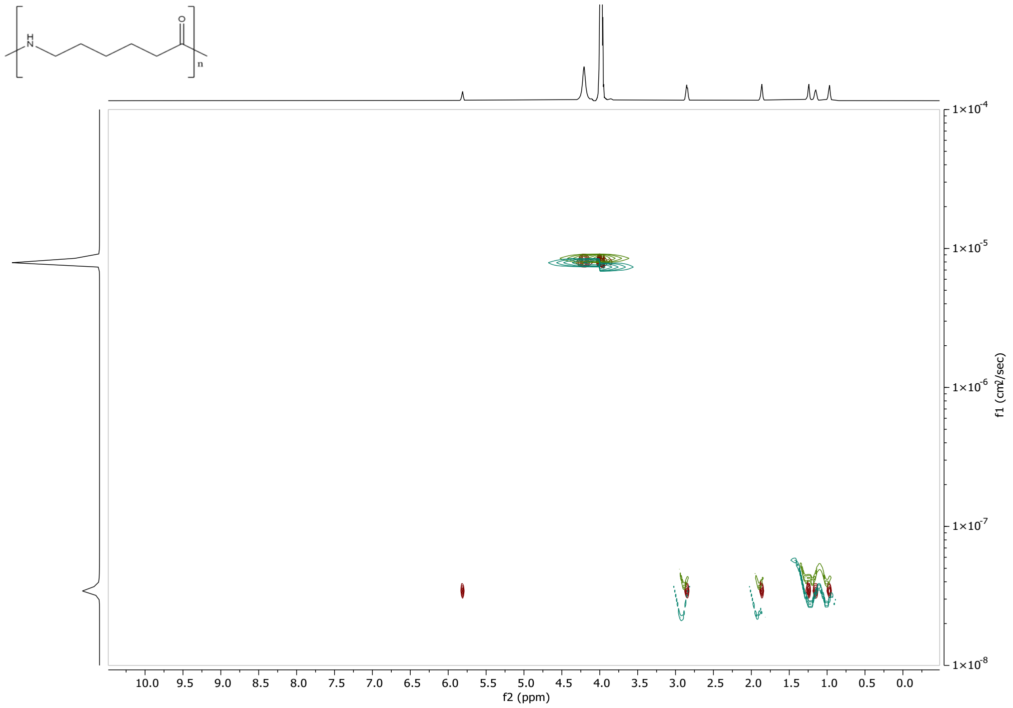

Figure S23. DOSY NMR of nylon 6. The blue signals correspond to nylon 6 after regeneration from HFIP. The green signals correspond to nylon 6 after regeneration from [dm$_3$-mTBDH][OAc]. The red signals correspond to the original nylon 6.

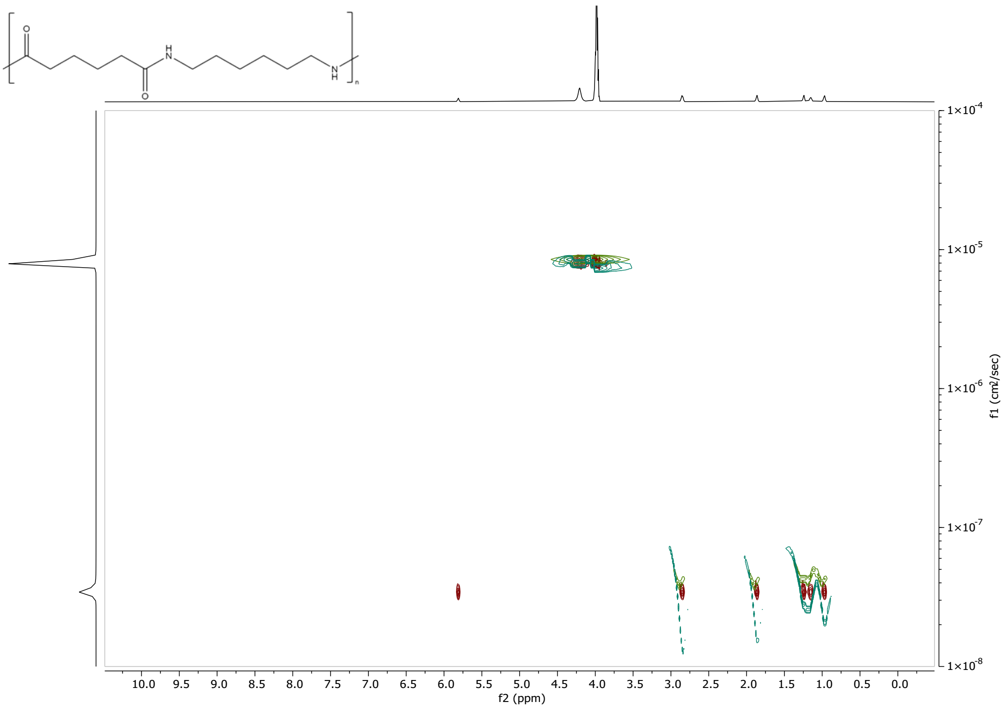

Figure S24 2D DOSY NMR of nylon 6,6 (P). The blue signals correspond to nylon 6,6 (P) after regeneration from HFIP. The green signals correspond to nylon 6,6 (P) after regeneration from [dm$_3$-mTBDH][OAc]. The red signals correspond to the original nylon 6,6 (P).

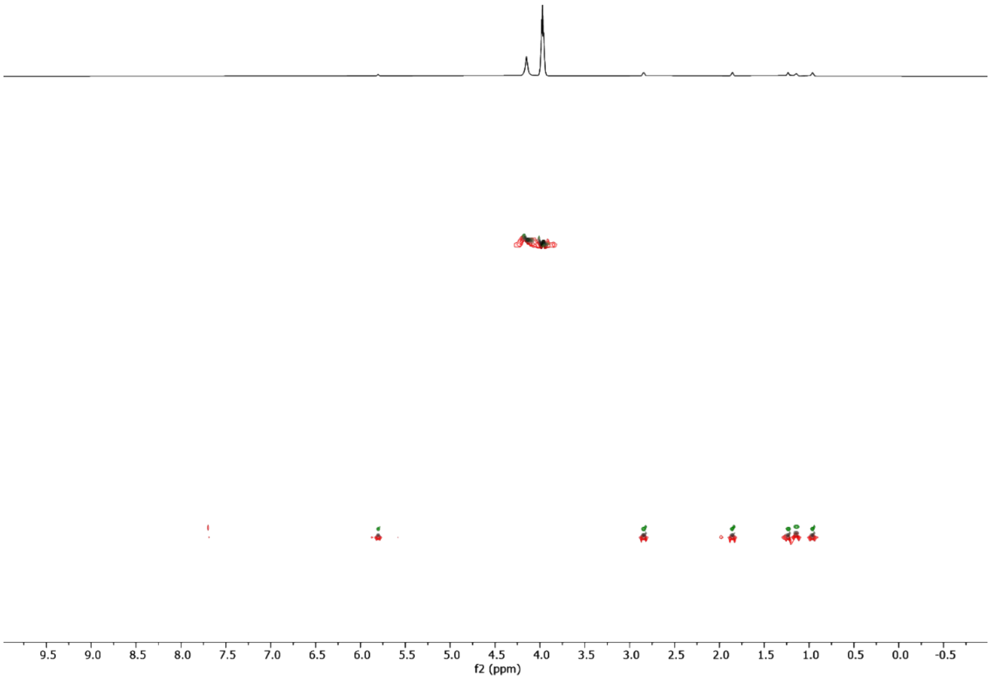

Figure S25. 2D DOSY NMR of nylon 6,6 (M). The black signals correspond to nylon 6,6 (M) after regeneration from HFIP. The green signals correspond to nylon 6,6 (M) after regeneration from [dm$_3$-mTBDH][OAc]. The red signals correspond to the original nylon 6,6 (M).

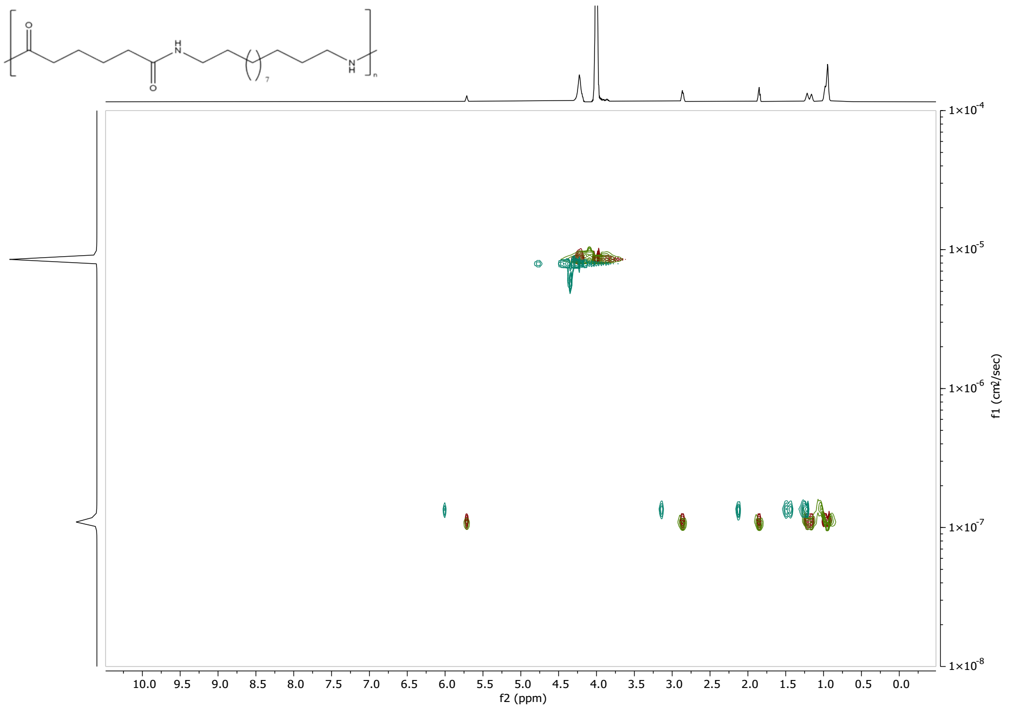

Figure S26. 2D DOSY NMR of nylon 6,12. The blue signals correspond to nylon 6 after regeneration from HFIP. The green signals correspond to nylon 6,12 after regeneration from [dm$_3$-mTBDH][OAc]. The red signals correspond to the original nylon 6,12.

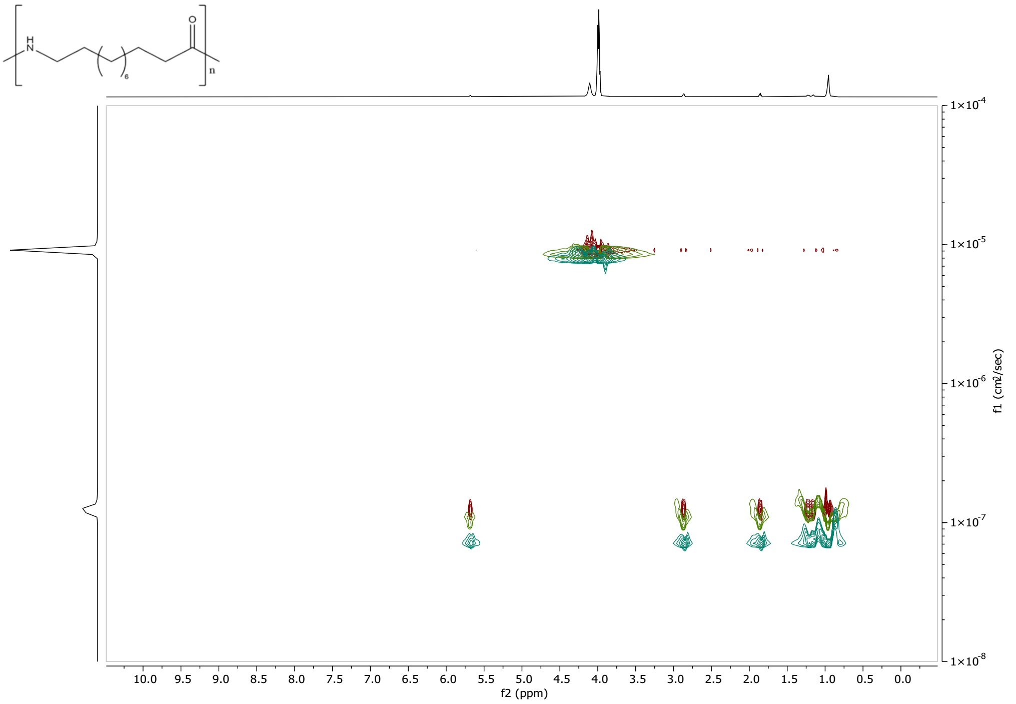

Figure S27. 2D DOSY NMR of nylon 11. The blue signals correspond to nylon 11 after regeneration from HFIP. The green signals correspond to nylon 11 after regeneration from [dm$_3$-mTBDH][OAc]. The red signals correspond to the original nylon 11.

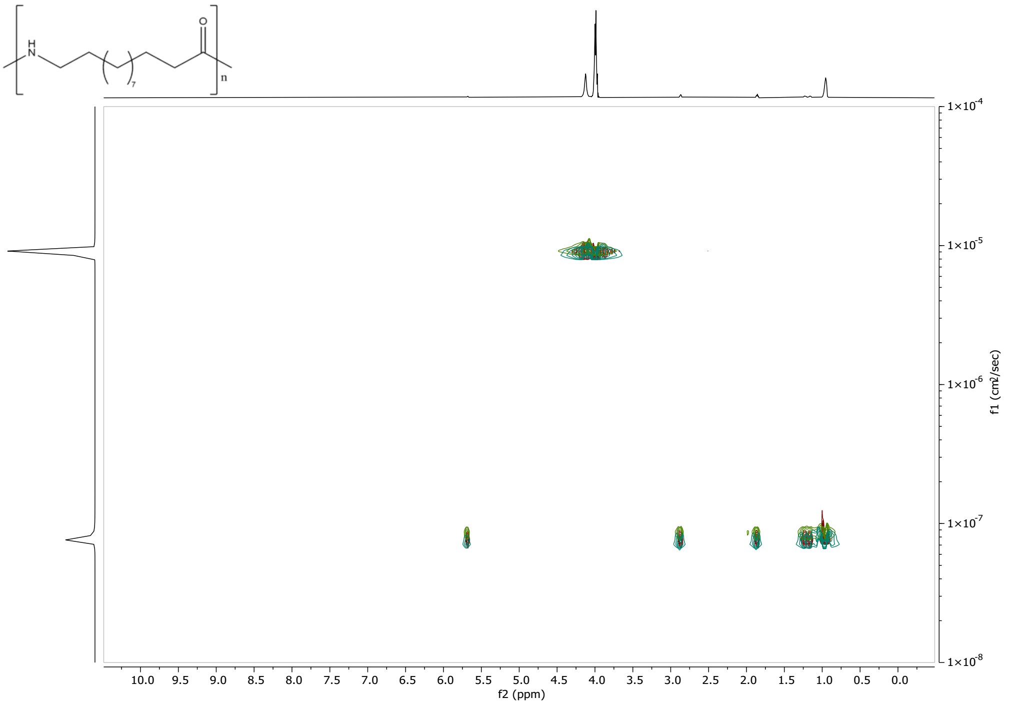

Figure S28. DOSY NMR of nylon 12. The black signals correspond to nylon 12 after regeneration from HFIP. The green signals correspond to nylon 12 after regeneration from [dm$_3$-mTBDH][OAc]. The red signals correspond to the original nylon 12.

b. <u>Characterization of regenerated nylon from [dm$_3$-mTBDH][OAc] cycle 1,2,3</u>

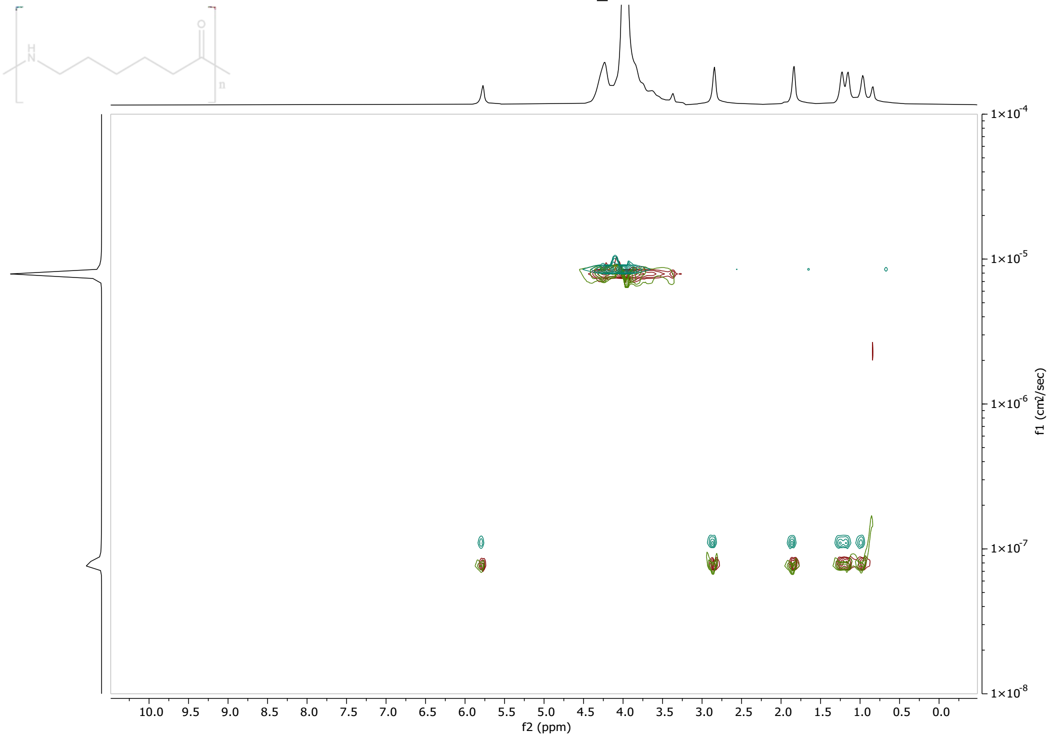

Figure S29. 2D DOSY NMR of nylon 6. The red signals correspond to nylon 6 after the first regeneration from [dm$_3$-mTBDH][OAc]. The green signals correspond to nylon 6 after the second regeneration after regeneration from [dm$_3$-mTBDH][OAc]. The blue signals correspond to nylon 6 after the third regeneration from [dm$_3$-mTBDH][OAc].

c. <u>Characterization of regenerated nylon from HFIP cycle 1,2,3</u>

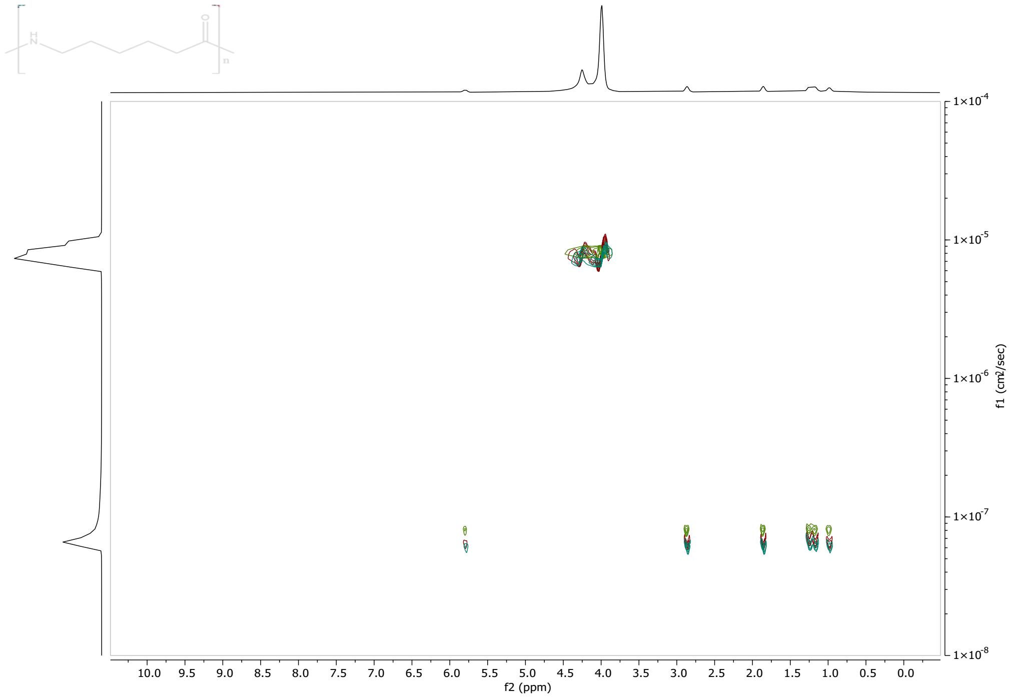

Figure S30. DOSY NMR of nylon 6. The red signals correspond to nylon 6 after the first regeneration from [dm$_3$-mTBDH][OAc]. The green signals correspond to nylon 6 after the second regeneration after regeneration from [dm$_3$-mTBDH][OAc]. The blue signals correspond to nylon 6 after the third regeneration from [dm$_3$-mTBDH][OAc].

Characterisation of nylons

a. InfraRed characterization

Infrared spectroscopy was conducted with a Shimadzu IRTracer-100 Fourier transform infrared spectrophotometer at room temperature by averaging 64 spectrum scans obtained within a range of the wavenumbers between 500 and 4000 cm^{-1}. The samples were powders for the regenerated nylons or thin films with a thickness of about 0.5 mm for the virgin nylons.

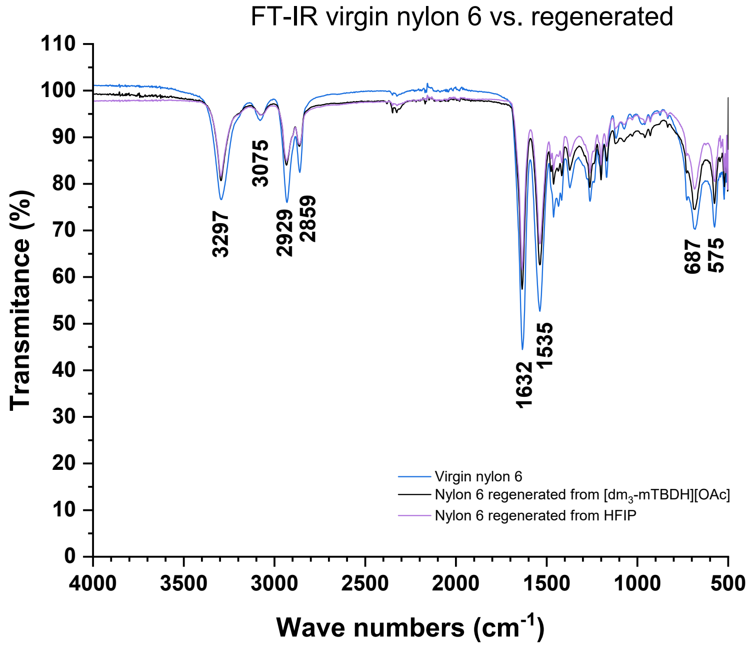

Figure S31. FT-IR spectra of nylon 6.

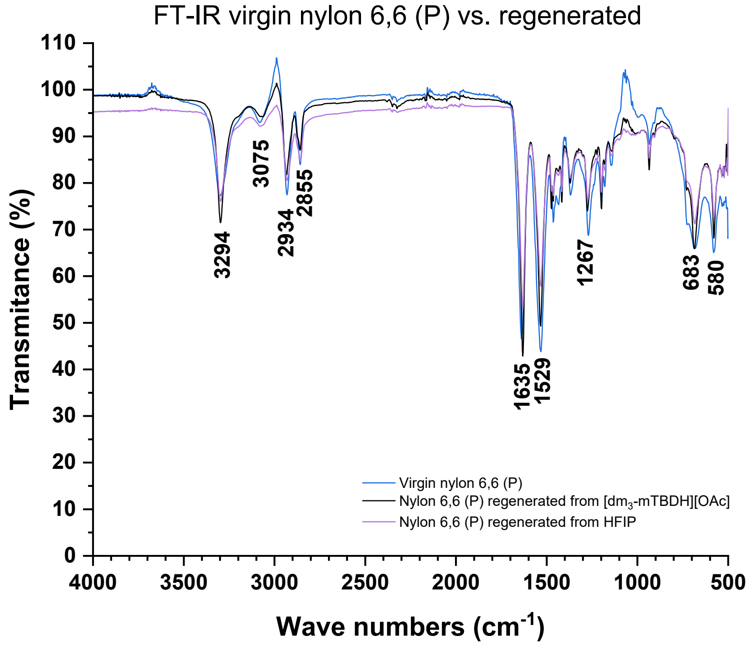

Figure S32. FT-IR spectra of nylon 6,6 (P).

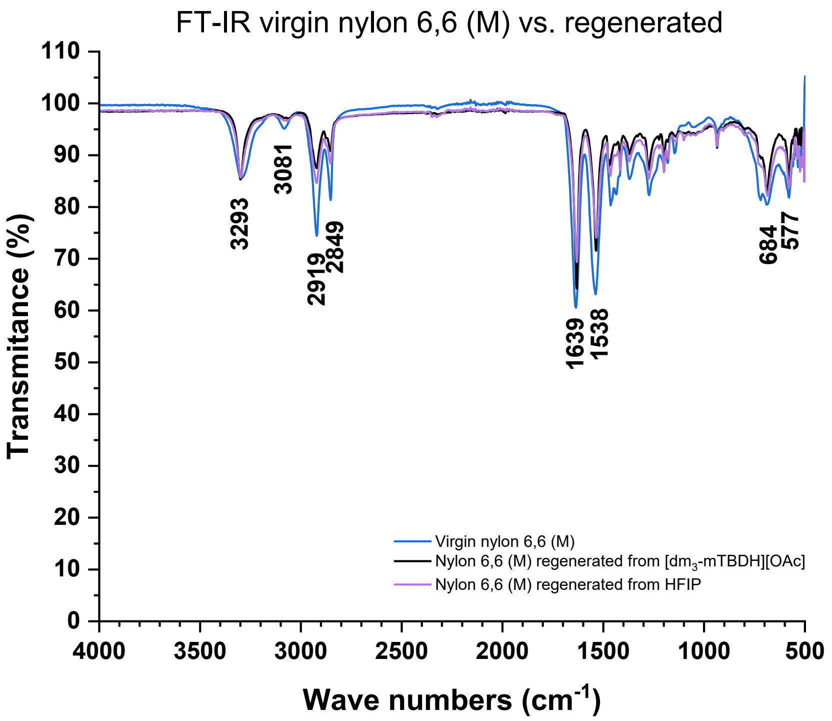

Figure S33. FT-IR spectra of nylon 6,6 (M).

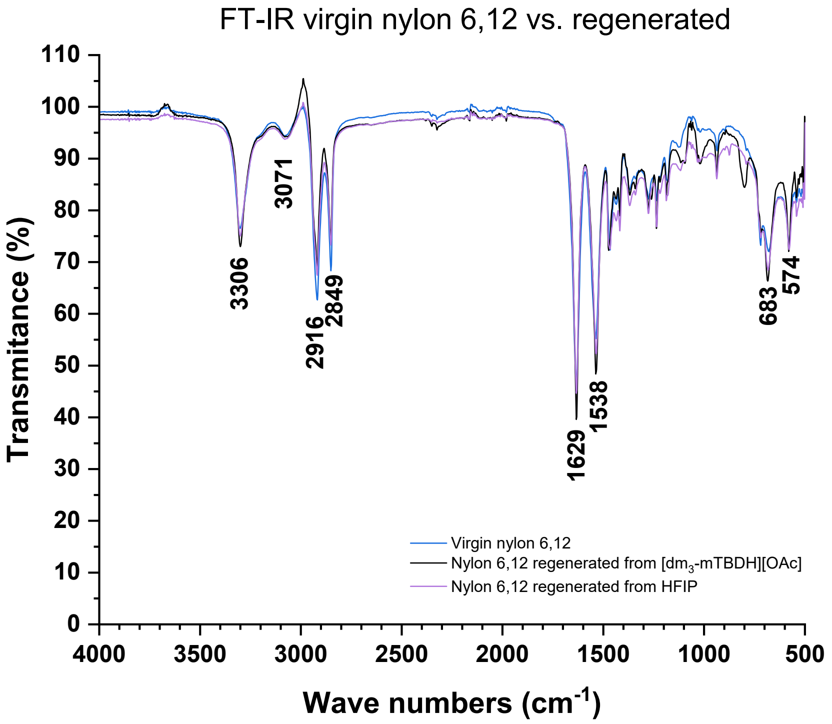

Figure S34. FT-IR spectra of nylon 6,12.

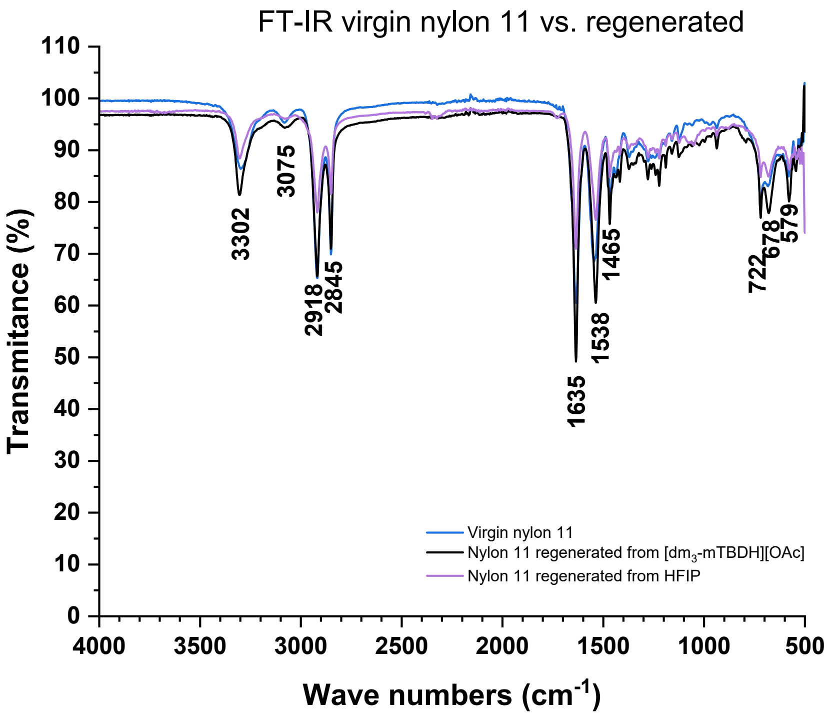

Figure S35. FT-IR spectra of nylon 11.

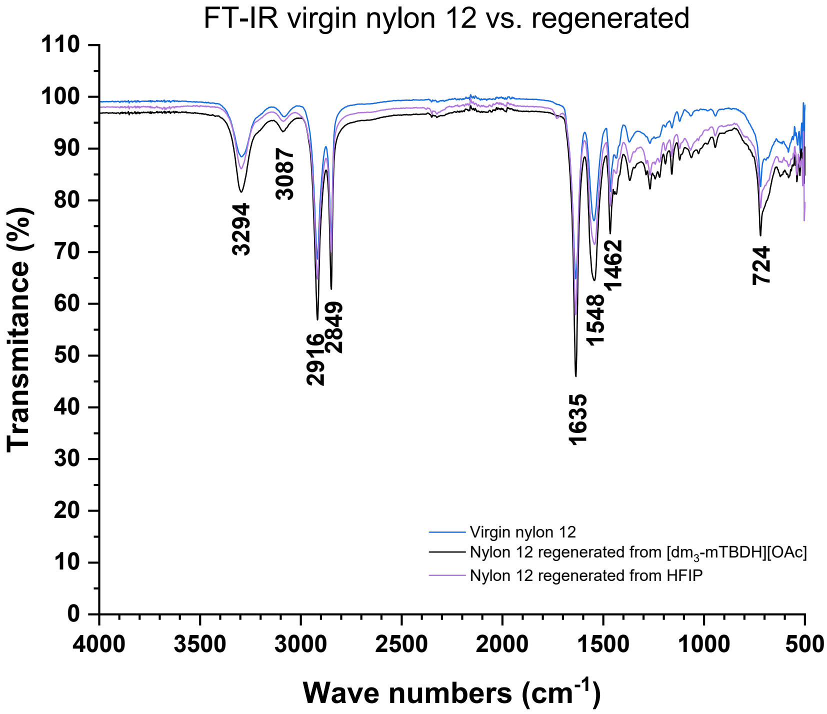

Figure S36. FT-IR spectra of nylon 12.

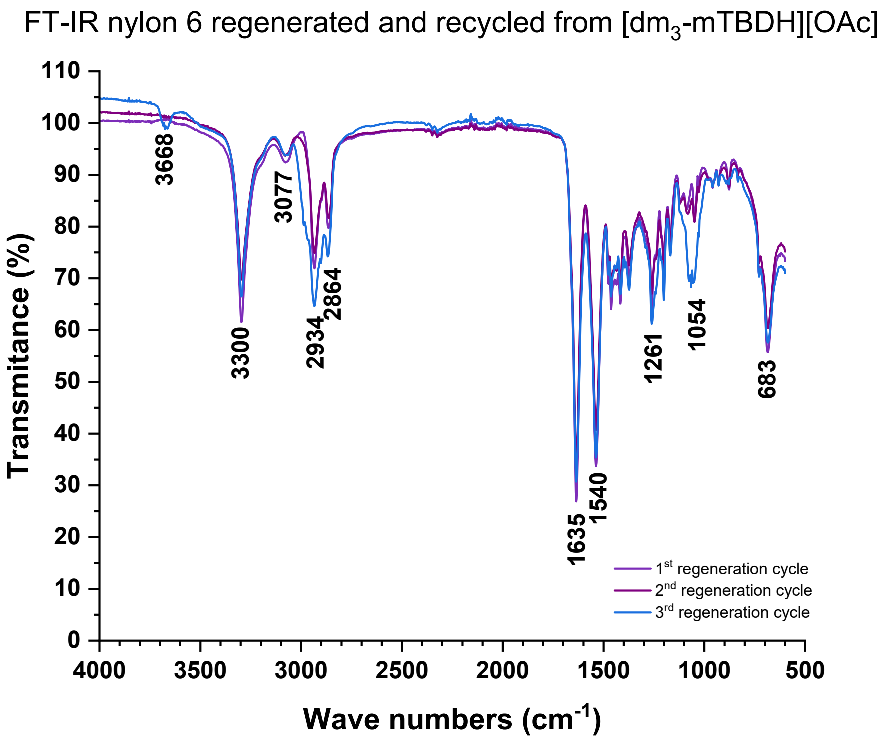

Figure S37. FT-IR spectra of Nylon 6 recycled three times in [dm$_3$-mTBDH][OAc].

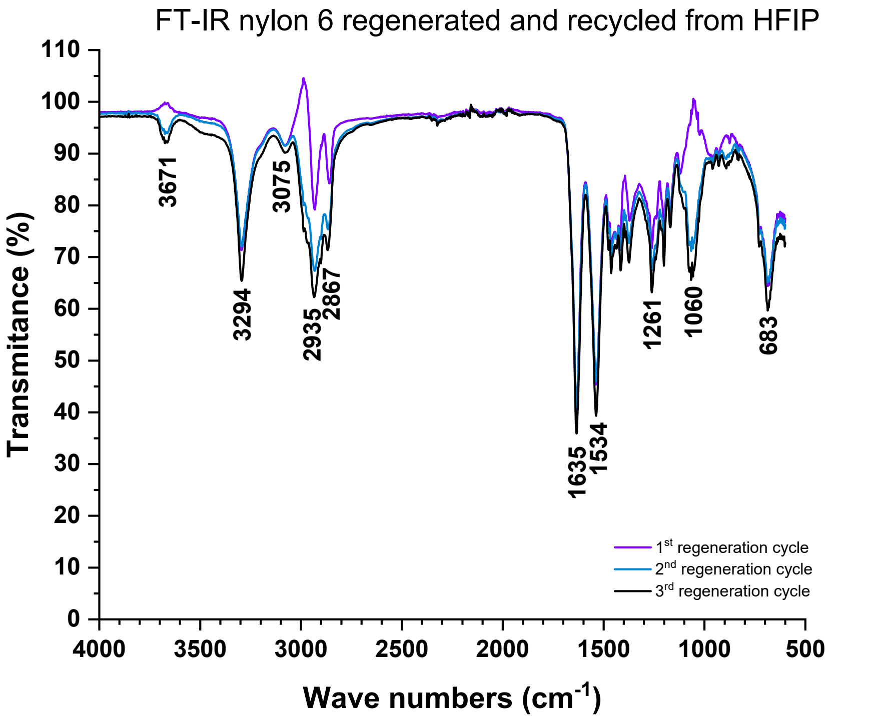

Figure S38. FT-IR spectra of Nylon 6 recycled three times in HFIP.

b. SEC chromatography

For SEC measurements, a setup consisting of Waters 515 HPLC pump, Waters 717 plus autosampler, Waters 2487 dual λ absorbance detector and Waters 2410 differential refractometer equipped with Agilent PL HFIPgel 300x7.5 mm column was used. Eluent was HFIP (Fluorochem) having 3.0 g/L of potassium trifluoroacetate (Sigma-Aldrich). Eluent flow rate was set at 0.40 ml/min and column temperature was set to 45 °C. Due to difficulties in dissolving some of the nylons in salt containing eluent, all nylon samples were dissolved in pure HFIP. Relative calibration was done using poly (methyl methacrylate) (PMMA) standards provided by Polymer Standards Service. The standards were dissolved in the same eluent. The samples were filtered through 0.22 μm PTFE filters (Clarify) prior to the SEC measurements. The number-average molecular weights (Mn) and the weight-average molecular weights (Mw) were directly obtained from the software of the device and are located in table S1.

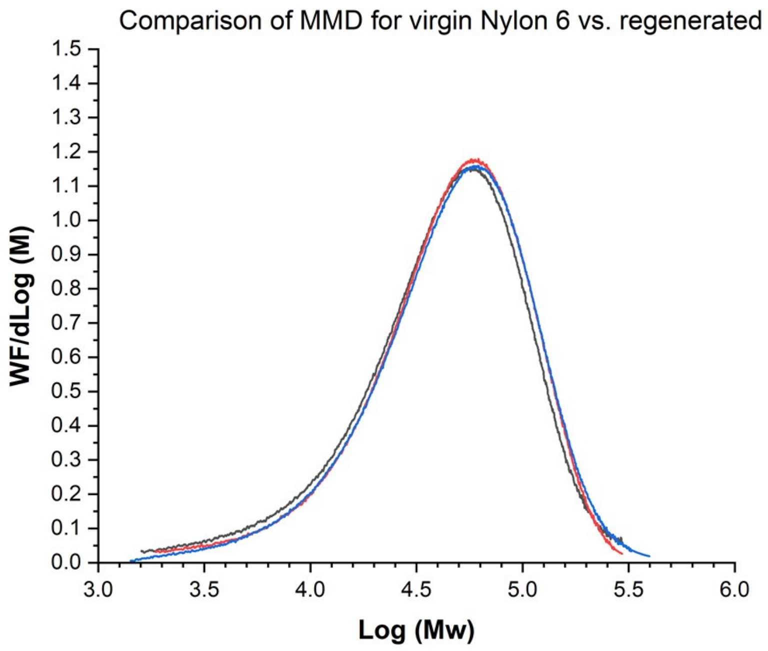

Figure S39. Molecular mass distribution (MMD) of nylon 6. The black curve represents the virgin nylon 6, the red curve represents nylon 6 after one dissolution in [dm$_3$-mTBDH][OAc] and the blue curve represents nylon 6 after one dissolution in HFIP.

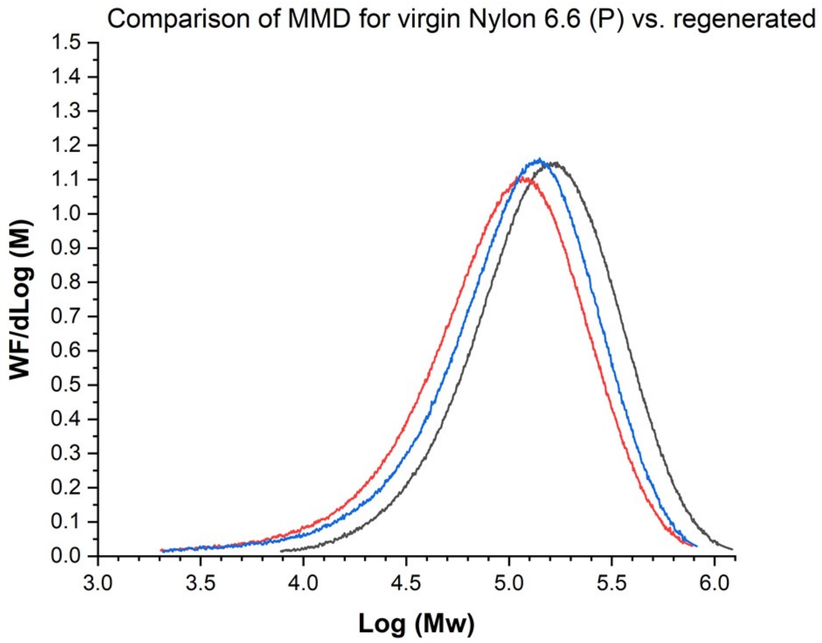

Figure S40. Molecular mass distribution of nylon 6,6 (P). The black curve represents the virgin nylon 6,6 (P), the red curve represents nylon 6,6 (P) after one dissolution in [dm$_3$-mTBDH][OAc] and the blue curve represents nylon 6,6 (P) after one dissolution in HFIP.

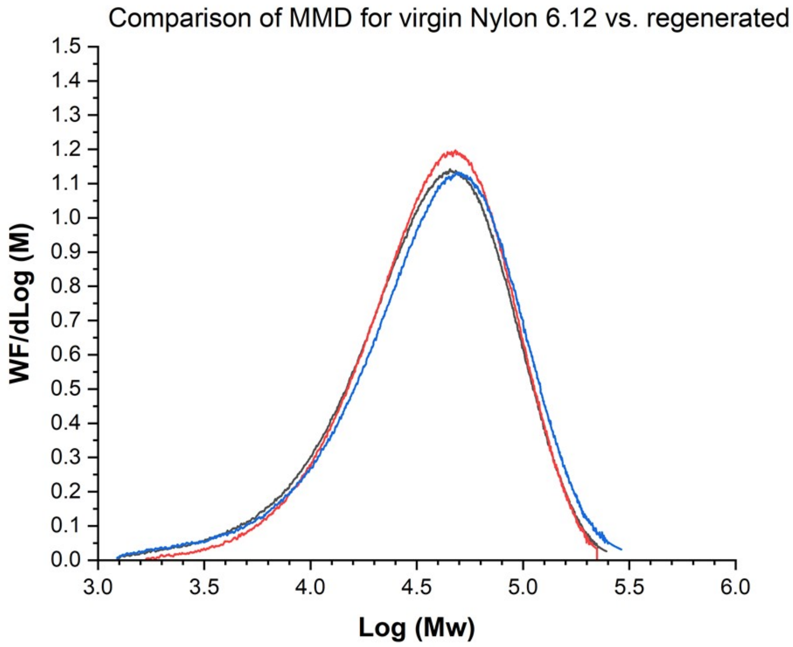

Figure S41. Molecular mass distribution of nylon 6,12. The black curve represents the virgin nylon 6,12, the red curve represents nylon 6,12 after one dissolution in [dm$_3$-mTBDH][OAc] and the blue curve represents nylon 6,12 after one dissolution in HFIP.

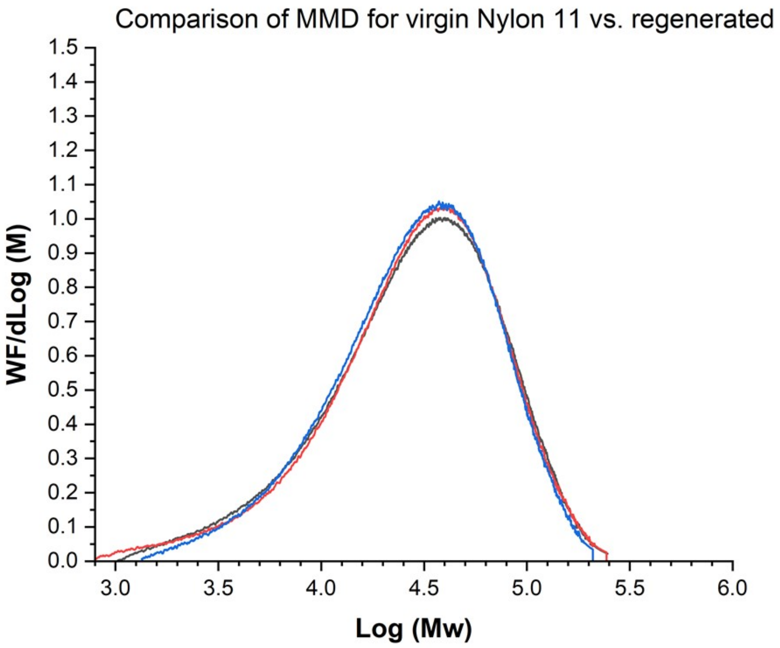

Figure S42. Molecular mass distribution of nylon 11. The black curve represents the virgin nylon 11, the red curve represents nylon 11 after one dissolution in [dm$_3$-mTBDH][OAc] and the blue curve represents nylon 11 after one dissolution in HFIP.

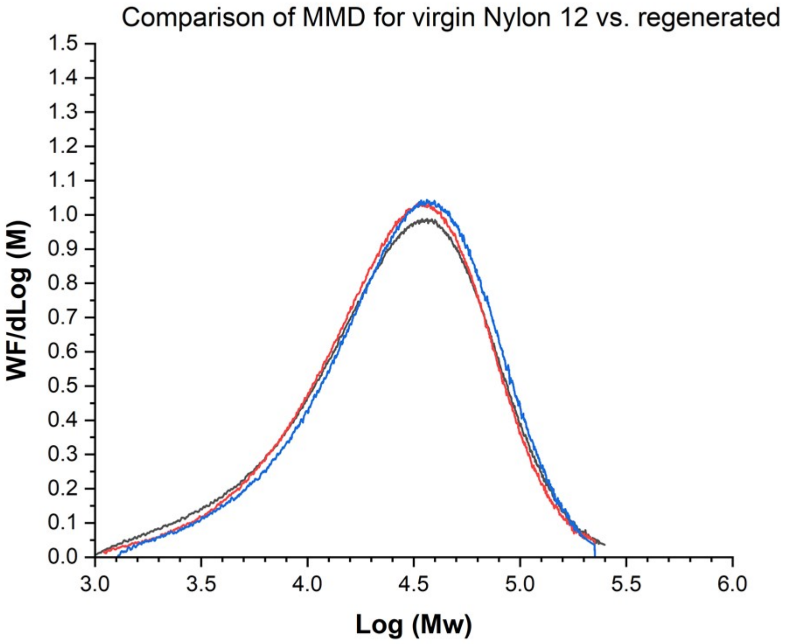

Figure S43. Molecular mass distribution of nylon 12. The black curve represents the virgin nylon 12, the red curve represents nylon 12 after one dissolution in [dm$_3$-mTBDH][OAc] and the blue curve represents nylon 12 after one dissolution in HFIP.

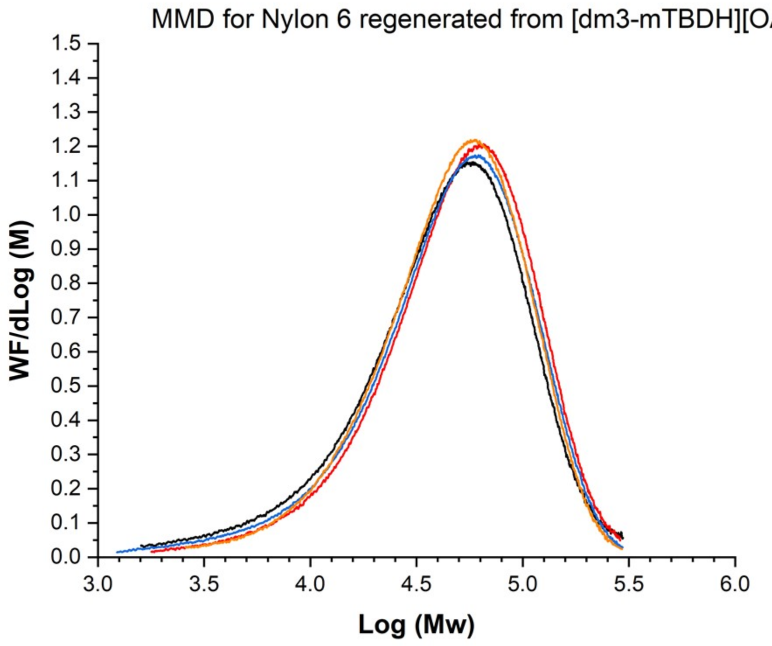

Figure S44. Molecular mass distribution of nylon 6 after three dissolution-regeneration cycles using [dm$_3$-mTBDH][OAc] as solvent. The black curve represents the virgin nylon 6, the red curve represents nylon 6 after the first cycle, the blue curve represents nylon 6 after the second cycle and the orange curve represents nylon 6 after the third cycle.

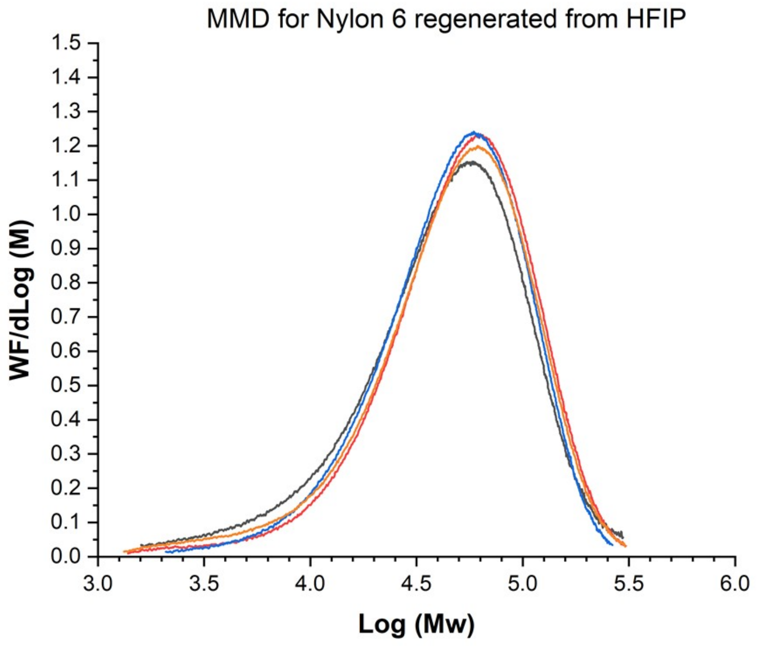

Figure S45. Molecular mass distribution of nylon 6 after three dissolution-regeneration cycles using HFIP as solvent. The black curve represents the virgin nylon 6, the red curve represents nylon 6 after the first cycle, the blue curve represents nylon 6 after the second cycle and the orange curve represents nylon 6 after the third cycle.

c. TGA characterization

Thermogravimetric measurements were performed using a Netzsch STA 449 F3 instrument. The samples were heated between 25 and 800 °C with a heating rate of 10 °C/min under nitrogen atmosphere (20 mL/min). Mass of the studied samples was in a range of 8 - 12 mg. The temperature onset was obtained using the tangent tool from the Origin software, however for clarity of figures, the tangents are not visualised.

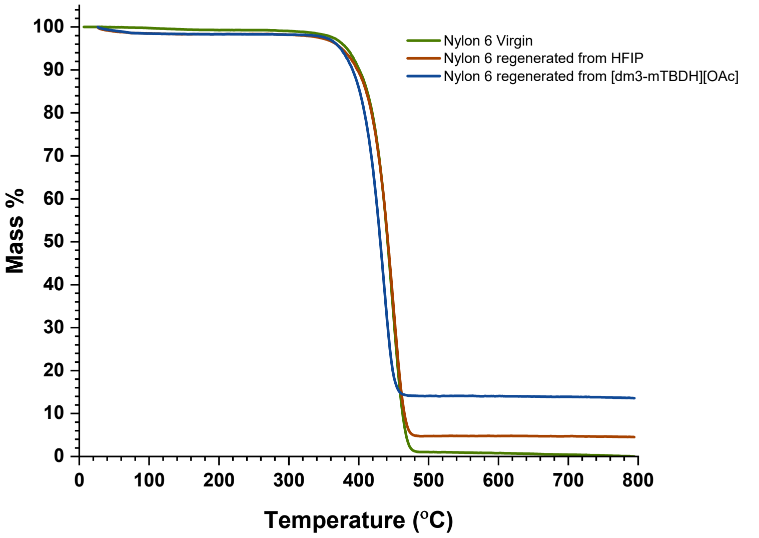

Figure S46. Thermographs of nylon 6. Temperature onset: virgin 417.65 °C, [dm$_3$-mTBDH][OAc] 417.51°C, HFIP 416.98 °C.

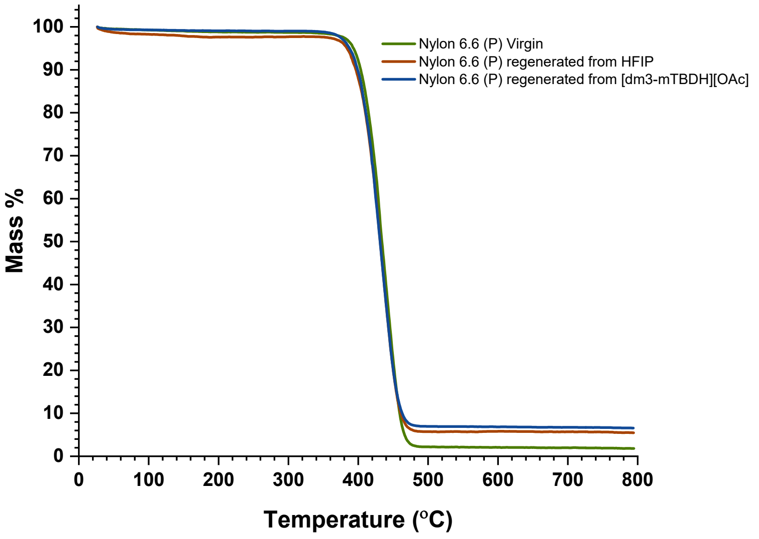

Figure S47. Thermographs of nylon 6,6 (P). Temperature onset: virgin 406.27 °C, [dm$_3$-mTBDH][OAc] 405.05 °C, HFIP 401.50 °C.

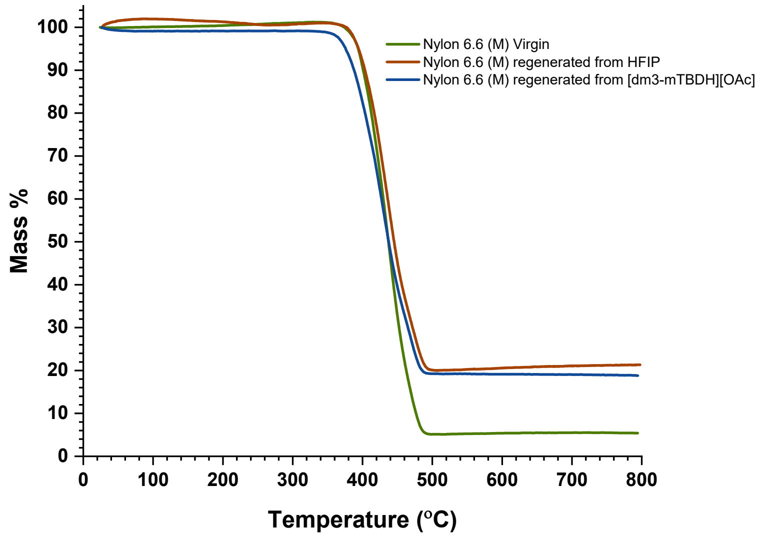

Figure S48. Thermograph of nylon 6,6 (M). Temperature onset: virgin 399.55 °C, [dm$_3$-mTBDH][OAc] 401.64 °C, HFIP 397.46 °C.

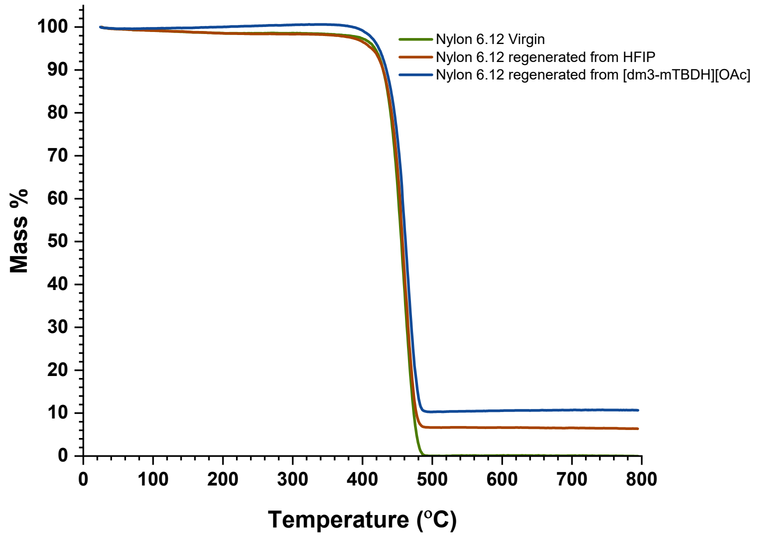

Figure S49. Thermograph of nylon 6,12. Temperature onset: virgin 434.21 °C, [dm$_3$-mTBDH][OAc] 437.88 °C, HFIP 439.88 °C.

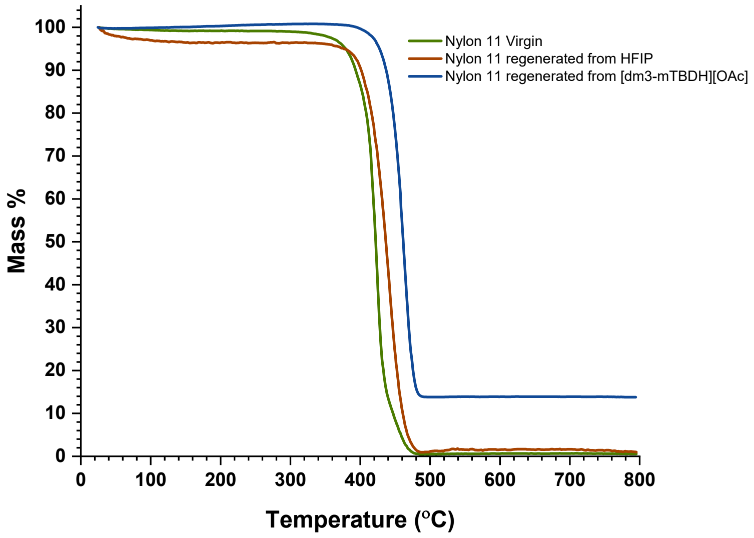

Figure S50. Thermograph of nylon 11. Temperature onset: virgin 408.28°C, [dm$_3$-mTBDH][OAc] 410.97 °C, HFIP 441.59 °C.

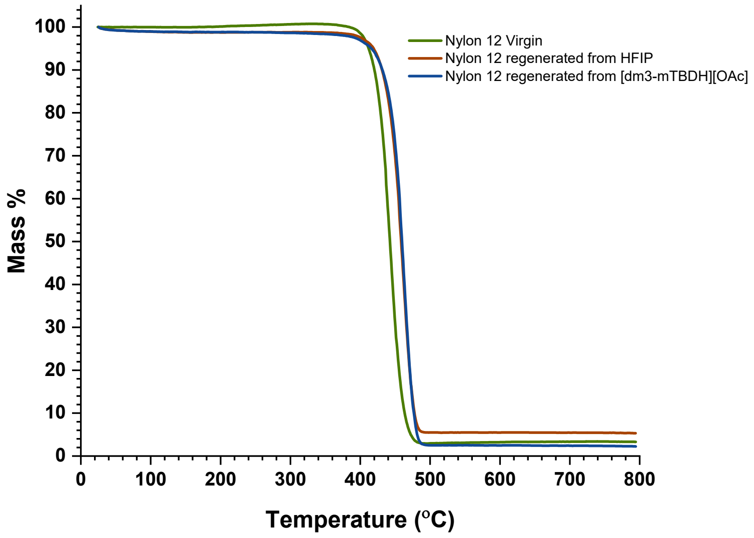

Figure S51. Thermograph of nylon 12. Temperature onset: virgin 422.29°C, [dm$_3$-mTBDH][OAc] 442.63 °C, HFIP 438.52 °C.

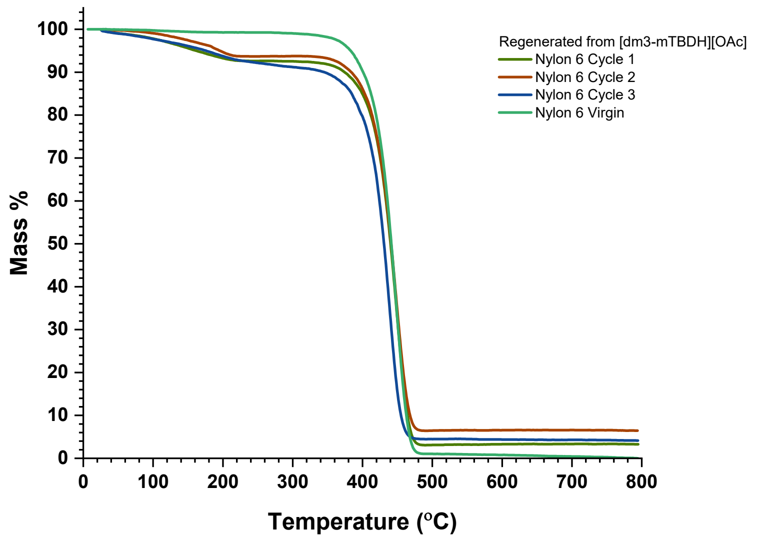

Figure S52. Thermograph of nylon 6 recycled from [dm$_3$-mTBDH][OAc]. Temperature onset: virgin 417.65 °C, cycle 1 417.33 °C, cycle 2 414.14 °C, cycle 3 410.82 °C.

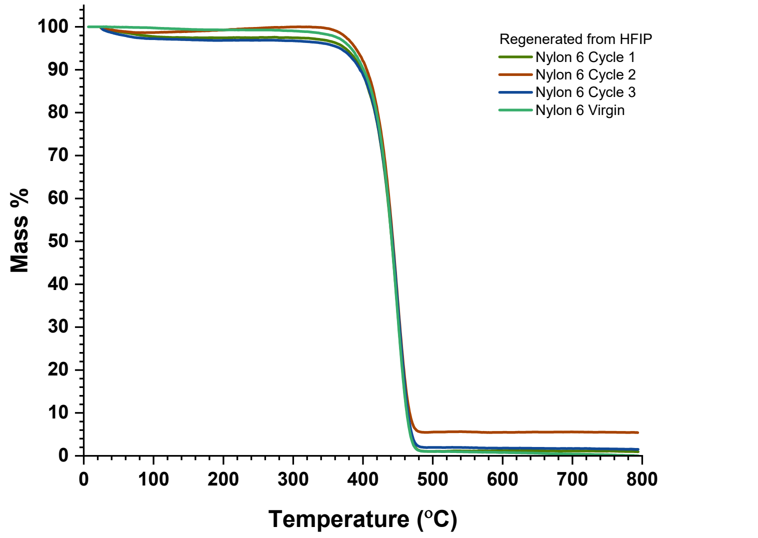

Figure S53. Thermograph of nylon 6 recycled from HFIP. Temperature onset: virgin 417.65 °C, cycle 1 416.98 °C, cycle 2 415.10 °C, cycle 3 418.65 °C.

d. DSC characterization

Calorimetric measurements were carried out using a TA Instrument Q 2000. The samples were heated from -20 to 280 °C under nitrogen atmosphere (50 mL/min). Heating and cooling rates were 10 °C/min. Mass of the studied polymer samples was in a range of 5 - 10 mg. The temperatures were directly obtained from the software of the device and are located in table S1.

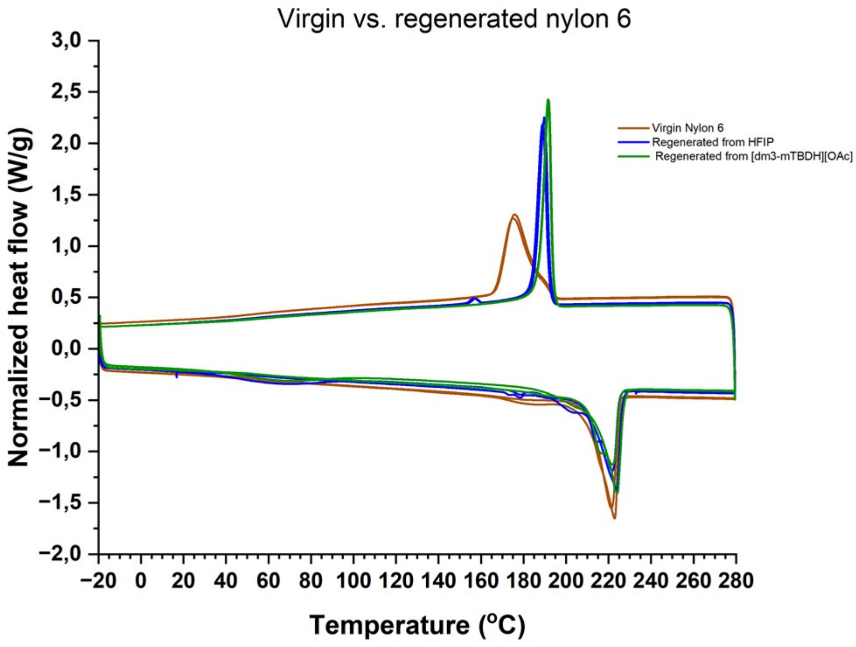

Figure S54. DSC heating and cooling curves of virgin nylon 6 and regenerated once from HFIP and [dm_3 -mTBDH][OAc]

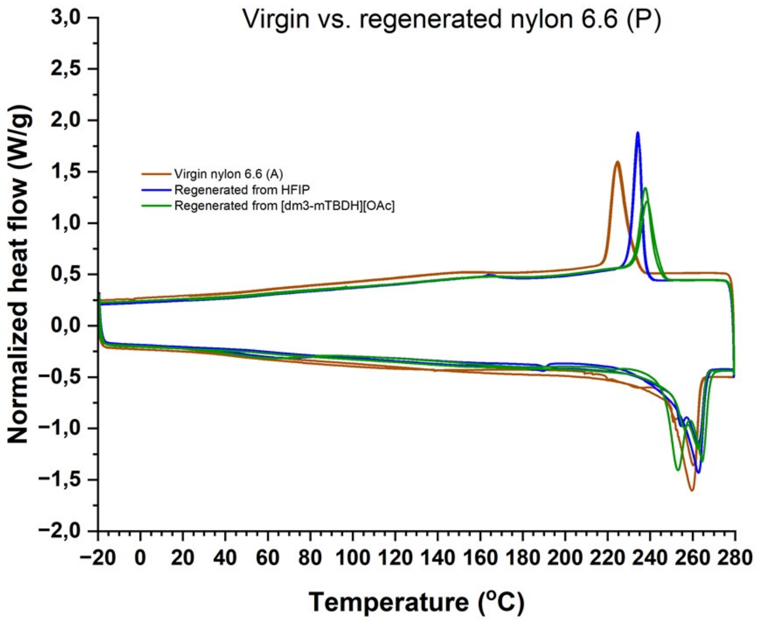

Figure S55. DSC heating and cooling curves of virgin nylon 6,6 (P) and regenerated once from HFIP and [dm_3-mTBDH][OAc]

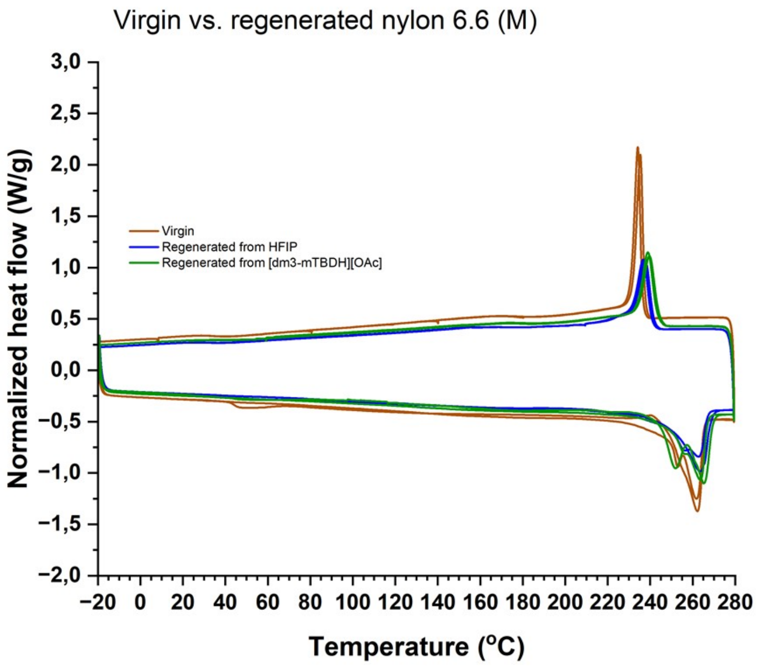

Figure S56. DSC heating and cooling curves of virgin nylon 6,6 (M) and regenerated once from HFIP and [dm$_3$-mTBDH][OAc]

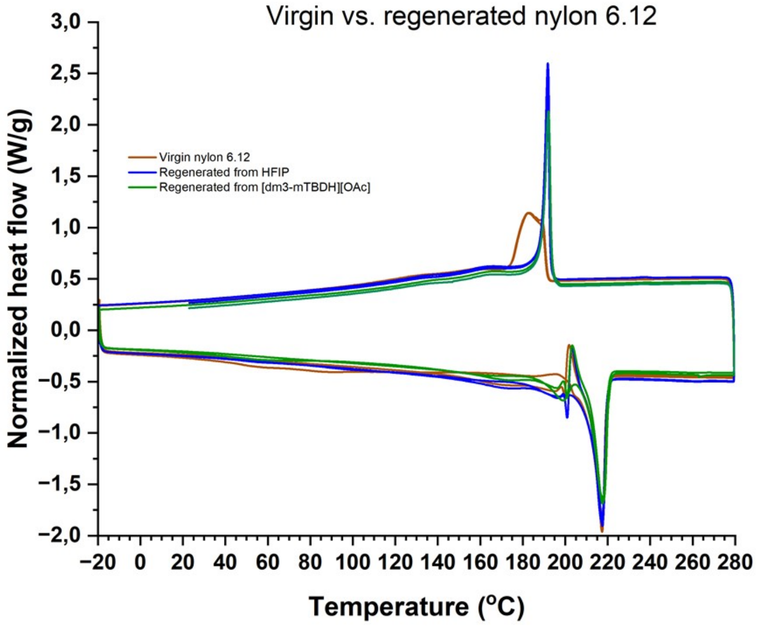

Figure S57. DSC heating and cooling curves of virgin nylon 6,12 and regenerated once from HFIP and [dm$_3$-mTBDH][OAc]

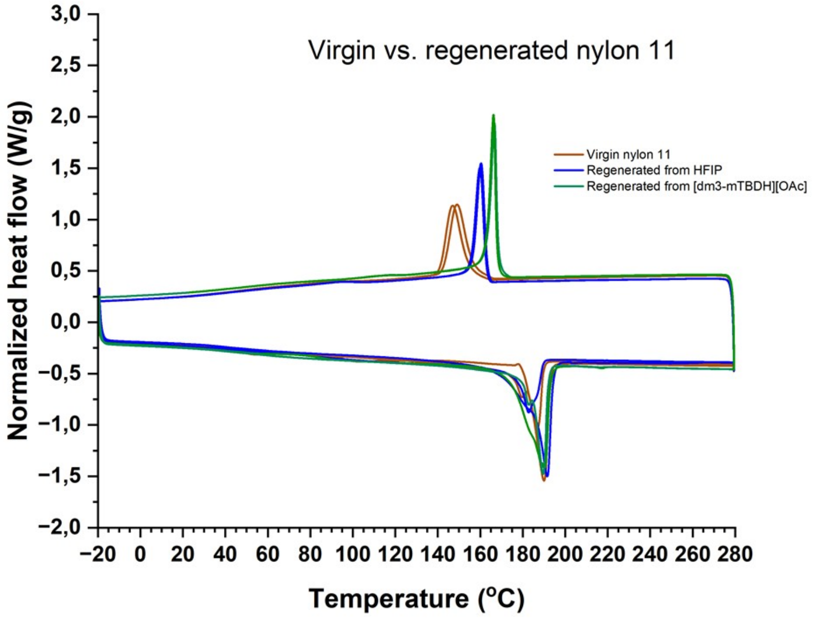

Figure S58. DSC heating and cooling curves of virgin nylon 11 and regenerated once from HFIP and [dm$_3$-mTBDH][OAc]

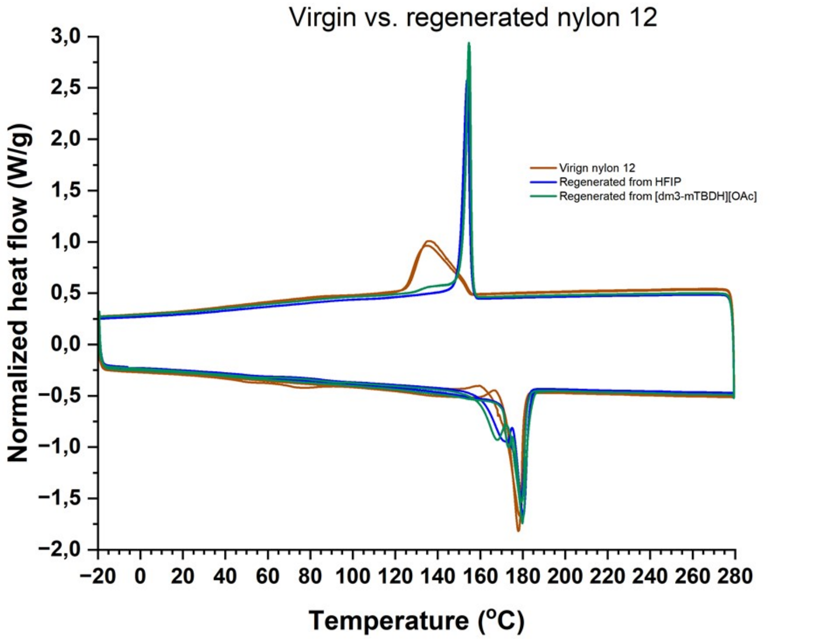

Figure S59. DSC heating and cooling curves of virgin nylon 12 and regenerated once from HFIP and [dm$_3$-mTBDH][OAc]

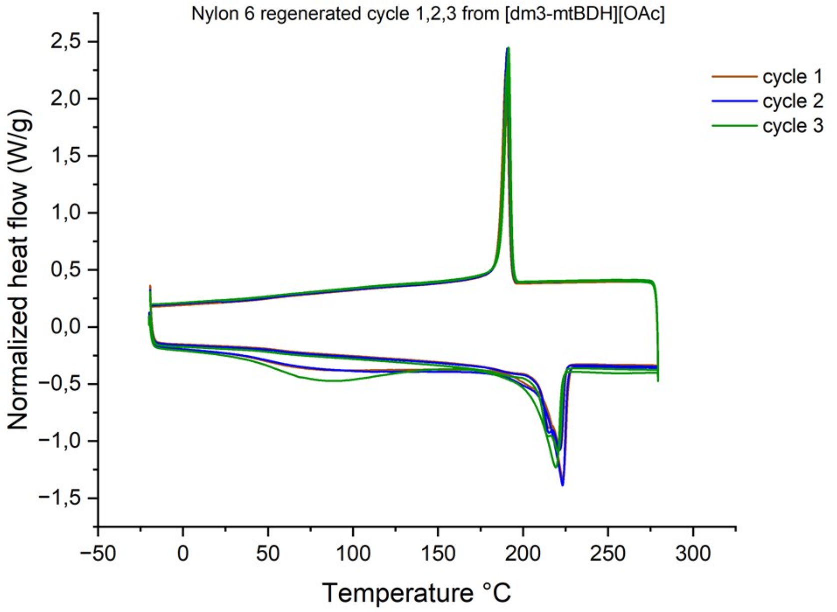

Figure S60 DSC of nylon 6 regenerated cycle 1,2,3 from [dm$_3$-mTBDH][OAc]

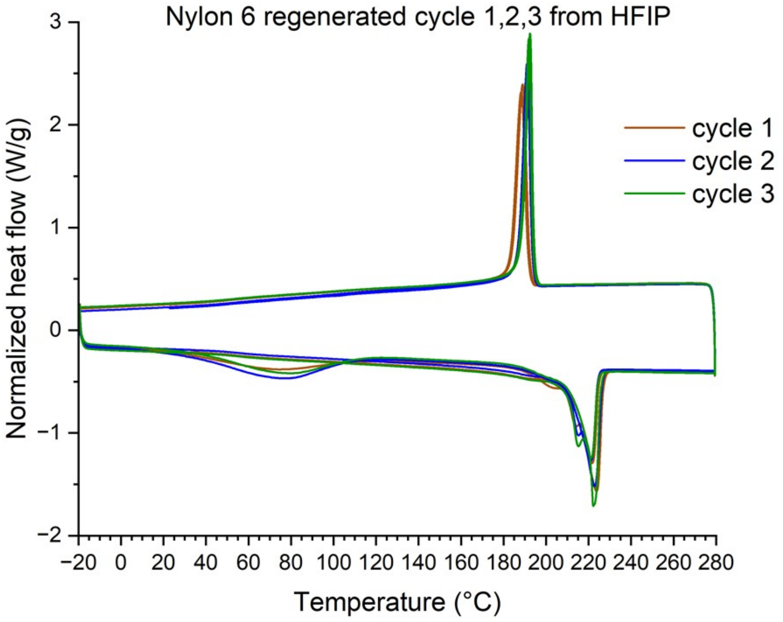

Figure S61 DSC of nylon 6 regenerated cycle 1,2,3 from HFIP

Nylon	Origin*	T_g (°C)	T_m (°C)	T_c(°C)	M_w	M_n	Đ= M_w/M_n	D_{pn}= $M_n/M_{w(AUG)}$	D_{pw}= $M_{wpolymer}/M_{w(AUG)}$	T_{onset} (°C)
Nylon 6	Virgin	53	221	176	58058	24782	2.34	219	513	418
	[dm_3-TBDH][OAc]	55	222	190	56262	26097	2.15	230	497	417
	HFIP	56	222	192	63432	28631	2.21	253	561	416
Nylon 6,6 (P)	Virgin	51	260	225	195414	98487	1.98	435	870	406
	[dm_3-TBDH][OAc]	54	263	234	111602	45695	2.44	202	493	405
	HFIP	52	262	238	149844	58727	2.55	260	662	402
Nylon 6,6 (M)	Virgin	138	262	235	N.D.	N.D.	N.D.	N.D.	N.D.	399
	[dm_3-TBDH][OAc]	135	264	238	N.D.	N.D.	N.D.	N.D.	N.D.	402
	HFIP	140	265	240	N.D.	N.D.	N.D.	N.D.	N.D.	397
Nylon 6,12	Virgin	41	217	183	48243	22229	2.17	72	155	434
	[dm_3-TBDH][OAc]	38	218	192	45010	15945	2.82	51	145	440
	HFIP	39	217	192	52887	23024	2.29	74	170	437
Nylon 11	Virgin	44	187	147	41622	16190	2.57	52	227	408
	[dm_3-TBDH][OAc]	45	190	160	34853	13162	2.64	42	190	442
	HFIP	47	183	166	40078	17637	2.27	57	219	410
Nylon 12	Virgin	34	178	135	38778	14374	2.7	46	212	422
	[dm_3-TBDH][OAc]	34	179	154	33887	12071	2.8	39	185	443
	HFIP	36	179	155	40361	16863	2.4	54	220	439

Table S1. Tabulated data from SEC, DSC and TGA measurements of nylon samples

* Origin refers to the solvent used for dissolution; N.D. not determined

Characterisation of the SB-ILs for nylon dissolution by ^{1}H and ^{13}C NMR

NMR reports:

[dm$_3$-mTBDH][OAc] mixture of isomers:

^{1}H NMR (400 MHz, DMSO) δ 3.26 (tt, *J* = 12.4, 5.9 Hz, 8H), 3.07 – 2.87 (m, 14H), 1.93 (p, *J* = 6.0 Hz, 2H), 1.83 (p, *J* = 5.8 Hz, 2H), 1.62 (s, 6H), 0.96 (d, *J* = 9.1 Hz, 12H).

^{13}C NMR (101 MHz, DMSO) δ 173.8, 150.5, 59.3, 58.9, 58.3, 50.2, 48.1, 48.0, 47.3, 40.0, 39.0, 38.1, 38.0, 28.8, 27.9, 25.7, 24.0, 24.0, 21.2, 21.0.

[mTBDH][OAc]:

^{1}H NMR (400 MHz, DMSO) δ 3.30 – 3.18 (m, 8H), 2.97 (s, 3H), 1.96 – 1.88 (m, 2H), 1.81 (p, *J* = 6.0 Hz, 2H), 1.60 (s, 3H).

^{13}C NMR (101 MHz, DMSO) δ 173.8, 151.4, 48.0, 47.7, 47.1, 39.0, 37.8, 25.8, 21.2, 21.1.

[mTBNH][OAc] mixture of isomers:

^{1}H NMR (400 MHz, DMSO) δ 3.55 – 3.36 (m, 8H), 3.30 – 3.12 (m, 8H), 2.98 (s, 3H), 2.90 (s, 3H), 1.97 (p, *J* = 5.9 Hz, 2H), 1.89 – 1.79 (m, 2H), 1.68 (s, 6H).

^{13}C NMR (101 MHz, DMSO) δ 174.1, 157.5, 155.5, 49.9, 48.0, 47.2, 47.1, 42.8, 42.6, 42.3, 40.0, 38.6, 37.6, 32.2, 24.9, 21.0, 20.3.

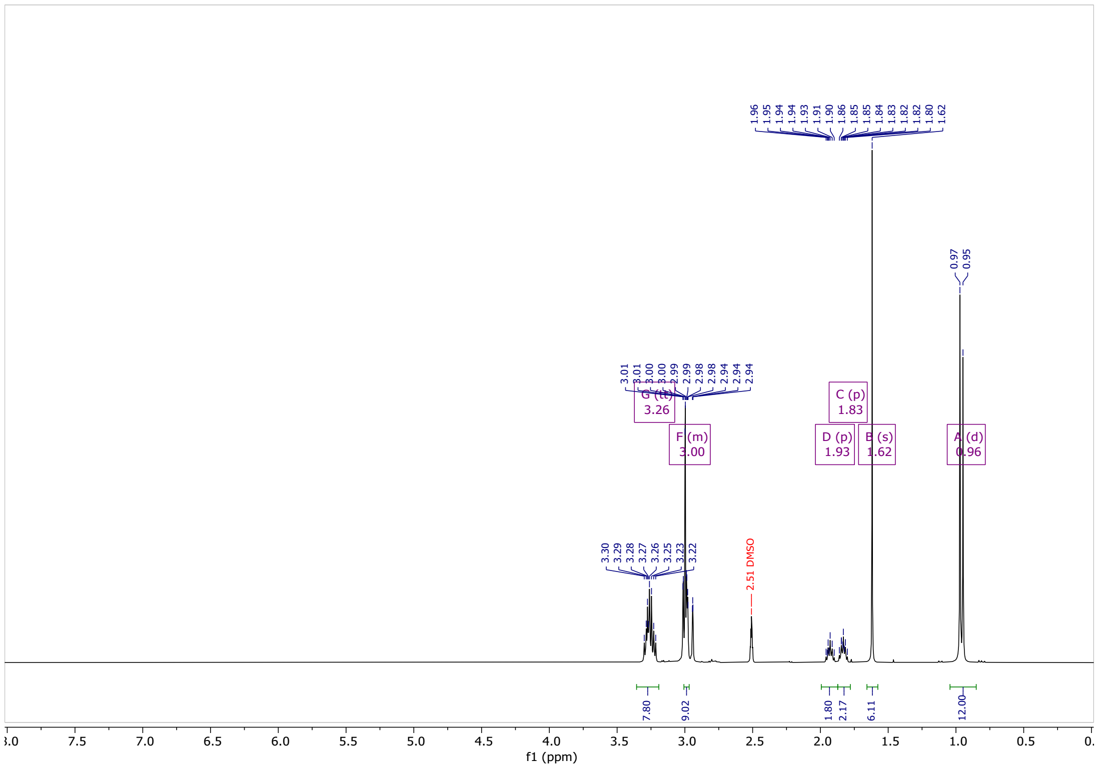

Figure S62 ^{1}H NMR of [dm$_3$-mTBDH][OAc]

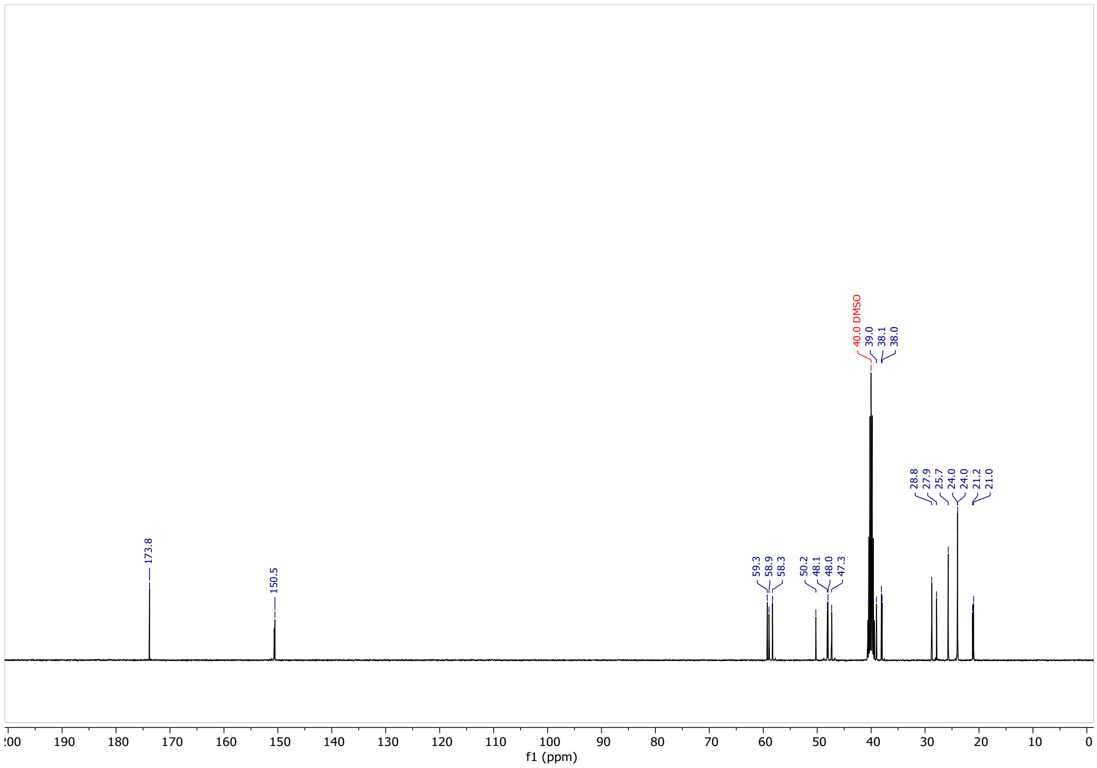

Figure S63 ¹³C NMR of [dm₃-mTBDH][OAc]

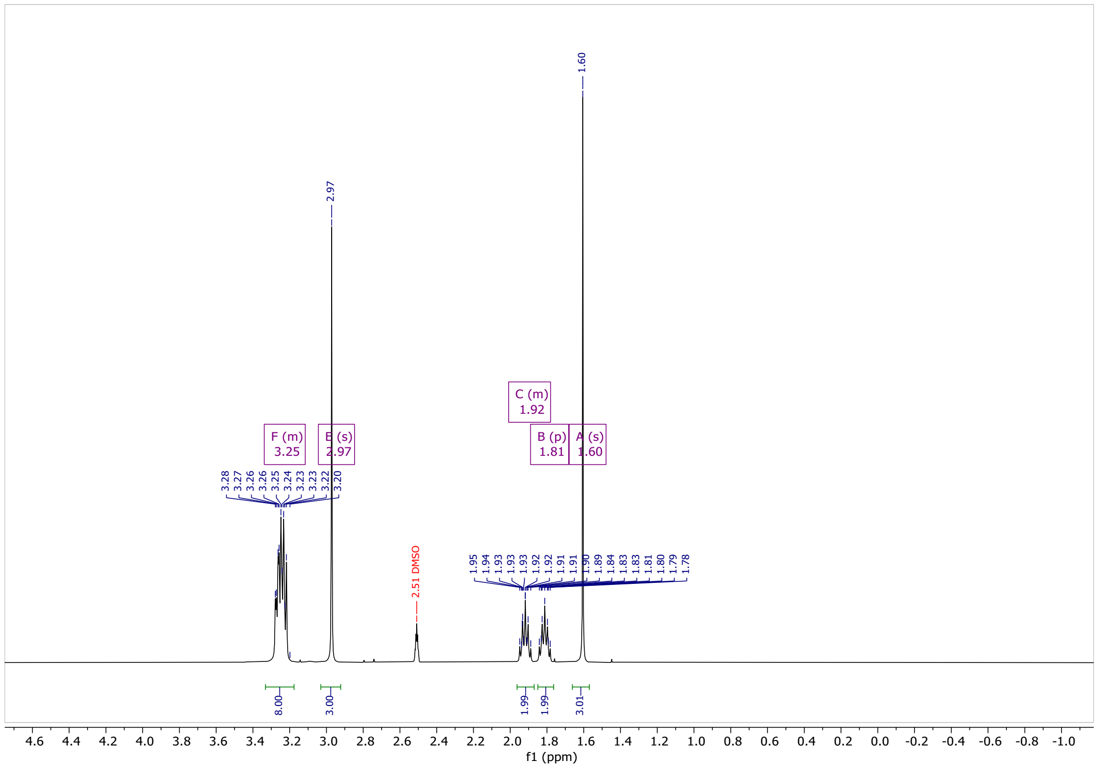

Figure S64 ¹H NMR of [mTBDH][OAc]

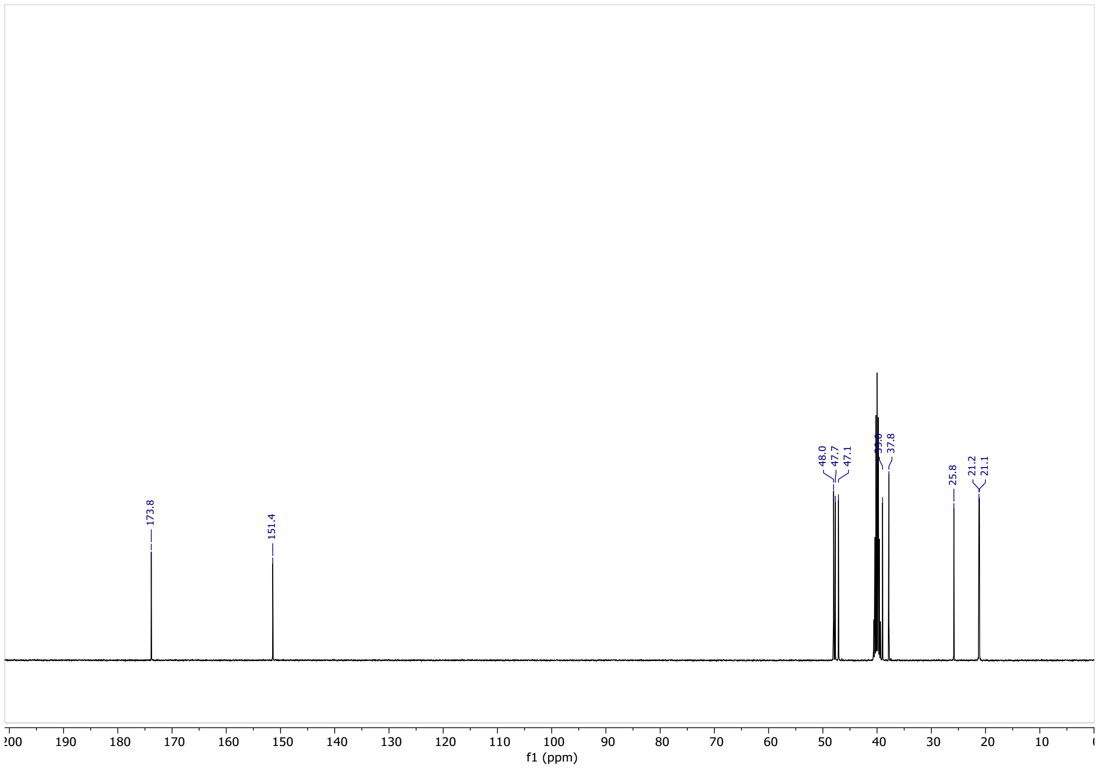

Figure S65 ^{13}C NMR of [mTBDH][OAc]

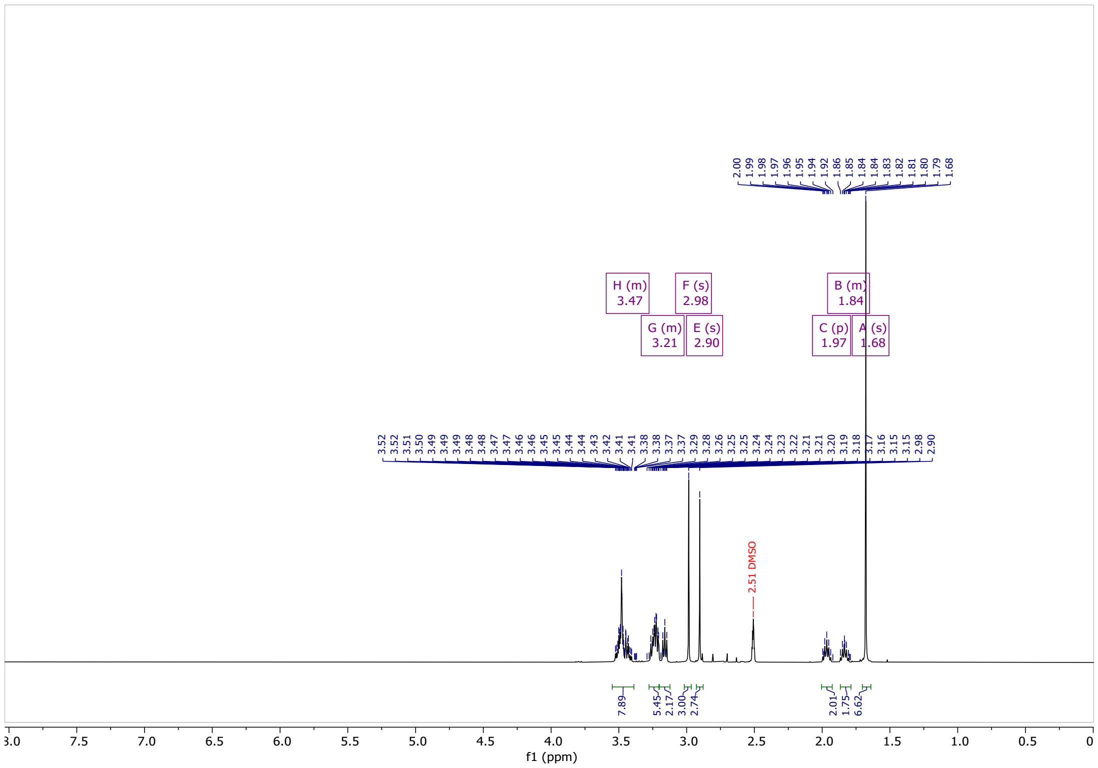

Figure S66 ^{1}H NMR of [mTBNH][OAc]

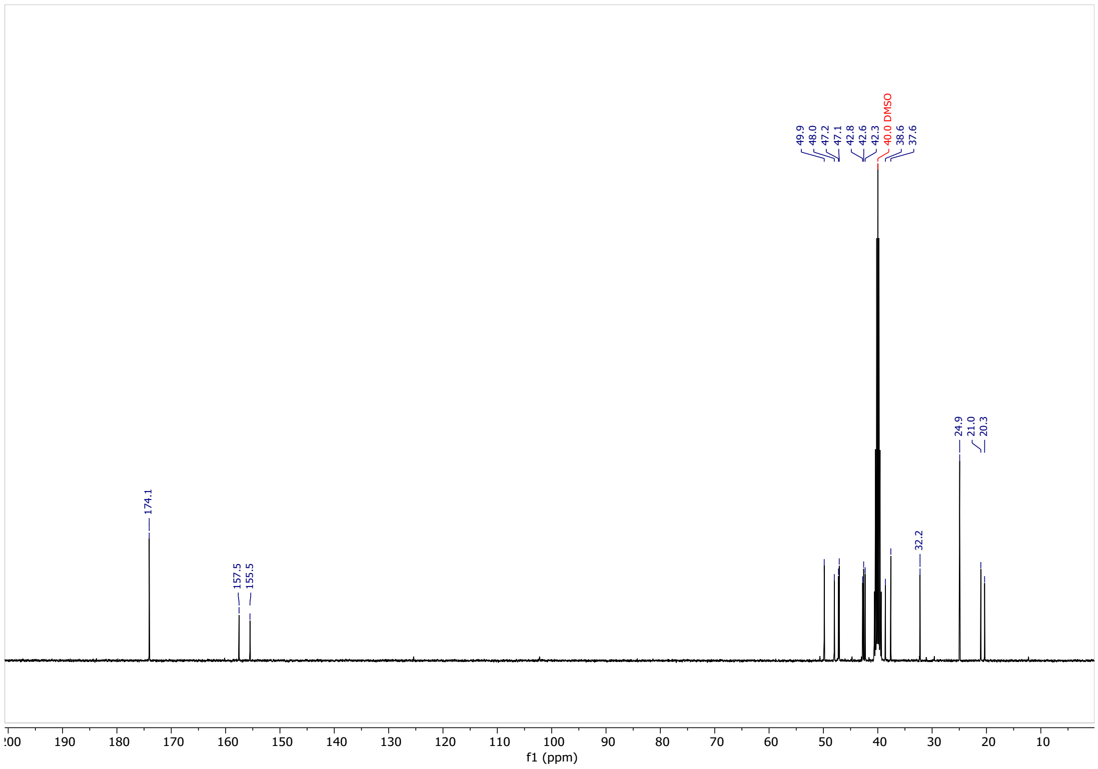

Figure S67 ¹³C NMR of [mTBNH][OAc]

Analysis of superbase ionic liquids after nylon dissolution by ¹H NMR

NMR reports:

[dm$_3$-mTBDH][OAc] after dissolution and regeneration of nylon 11.

¹H NMR (400 MHz, DMSO) δ 3.34 – 3.19 (m, 8H), 3.07 – 2.89 (m, 14H), 1.94 (ddt, J = 11.9, 9.0, 4.4 Hz, 2H), 1.89 – 1.82 (m, 2H), 1.66 (s, 6H), 0.97 (d, J = 5.1 Hz, 12H).

[dm$_3$-mTBDH][OAc] after dissolution and regeneration of nylon 6,6 (P).

¹H NMR (400 MHz, DMSO) δ 3.36 – 3.22 (m, 8H), 3.09 – 2.92 (m, 14H), 1.99 – 1.90 (m, 2H), 1.85 (h, *J* = 5.9 Hz, 2H), 1.71 (s, 6H), 0.97 (d, *J* = 2.9 Hz, 12H).

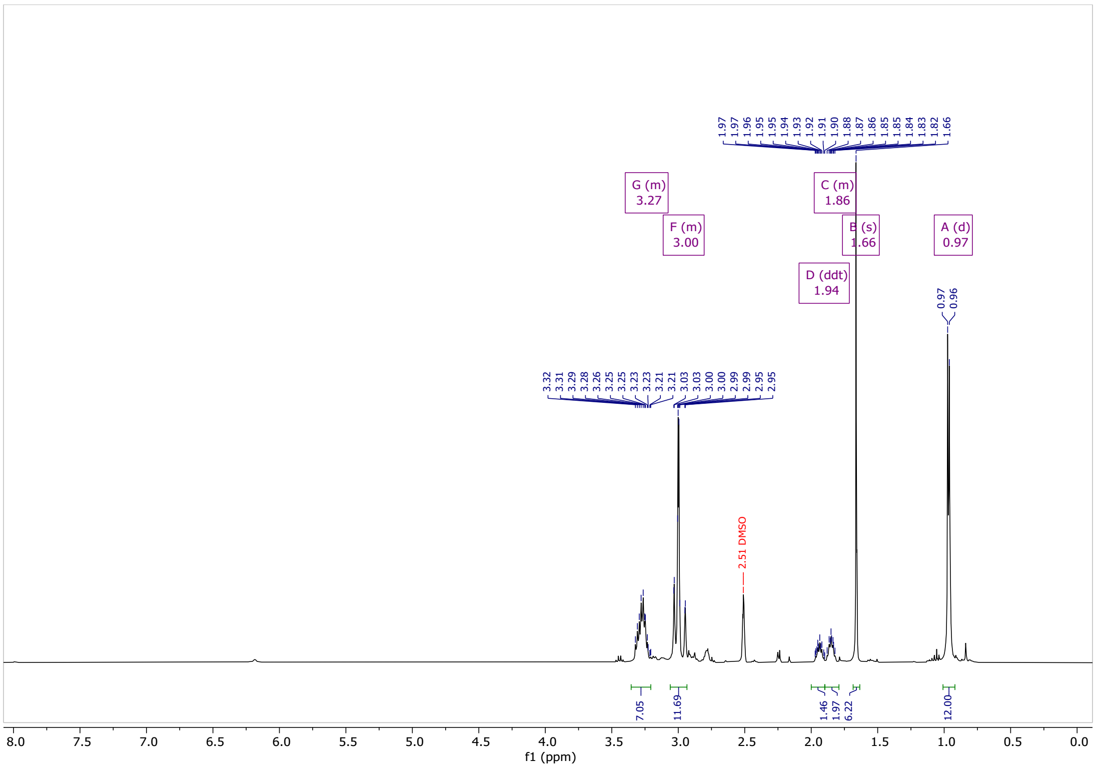

Figure S68 ^{1}H NMR of [dm_3-mTBDH][OAc] after dissolution and regeneration of nylon 11.

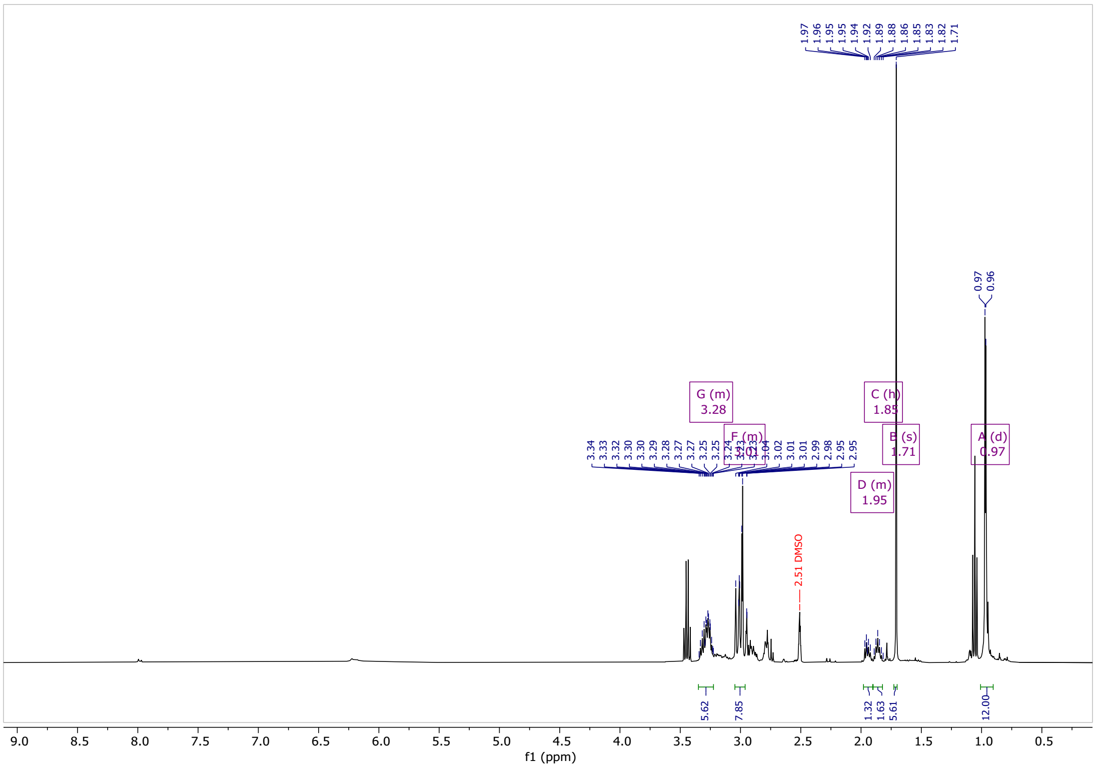

Figure S69 ^{1}H NMR of [dm_3-mTBDH][OAc] after dissolution and regeneration of nylon 6,6 (P).

Recycling of superbase ionic liquid

In a 100 mL round bottom flask equipped with an oval stirrer bar (20 x 10 mm), 1 g (10 % (by mass)) of nylon 6 and 10 g of [dm_3-mTBDH][OAc] were charged. Then the suspended nylon sample was immersed into a preheated silicon oil bath (100 °C) for 20 hours. After that to the hot nylon solution was added 150 mL EtOH in order to precipitate the nylon 6 material from the solutions and the heating was ceased. After that the mixture is stirred for an hour at room temperature and then the solids were filtered over a sintered funnel (Pore size 4) and were further transferred back into 150ml fresh ethanol. This step was repeated in total 3 times to wash the nylon as much as possible from the used solvent. After this, the separated nylon was dried under high vacuum at room temperature over 20 hours. The ethanol washings were combined, and the ethanol was evaporated. The concentrated ionic liquid was further dried under high vacuum at room temperature over 20 hours. Then a 0.1 g sample was taken for further analysis. After the drying and analysis, the same ionic liquid was re-used to dissolve a fresh batch of nylon 6. The dissolution and regeneration follow the same procedure as mentioned earlier observing the correct concentration (see table S2). Once the five regenerations cycle are over, the dried ionic liquid can be stored in a sealed vial at room temperature and can be re-used further.

Note: *These experiments were done in small scale, which increases the possibility for experimental errors.*

NMR reports:

Cycle 1: **^{1}H NMR** (400 MHz, DMSO) δ 3.33 – 3.20 (m, 8H), 3.08 – 2.90 (m, 14H), 1.93 (p, *J* = 6.0 Hz, 2H), 1.84 (p, *J* = 6.0 Hz, 2H), 1.62 (s, 6H), 0.97 (d, *J* = 6.7 Hz, 12H).

Cycle 2: **^{1}H NMR** (400 MHz, DMSO) δ 3.27 (ddt, *J* = 12.1, 10.0, 5.9 Hz, 8H), 3.09 – 2.88 (m, 14H), 1.93 (p, *J* = 6.0 Hz, 2H), 1.88 – 1.81 (m, 2H), 1.63 (s, 6H), 0.96 (d, *J* = 6.4 Hz, 12H).

Cycle 3: **^{1}H NMR** (400 MHz, DMSO) δ 3.27 (ddt, *J* = 12.4, 9.4, 5.9 Hz, 8H), 3.12 – 2.89 (m, 14H), 1.93 (p, *J* = 6.0 Hz, 2H), 1.85 (p, *J* = 5.8 Hz, 2H), 1.64 (s, 6H), 0.97 (d, *J* = 5.5 Hz, 12H).

Cycle 4: **^{1}H NMR** (600 MHz, DMSO) δ 3.33 – 3.21 (m, 8H), 3.00 (d, *J* = 3.1 Hz, 14H), 1.93 (p, *J* = 6.2 Hz, 2H), 1.85 (p, *J* = 5.9 Hz, 2H), 1.65 (s, 6H), 0.96 (d, *J* = 8.4 Hz, 12H).

Cycle 5: **^{1}H NMR** (500 MHz, DMSO) δ 3.27 (ddt, *J* = 15.4, 11.4, 6.0 Hz, 8H), 3.00 (t, *J* = 2.4 Hz, 14H), 1.94 (h, *J* = 6.0 Hz, 2H), 1.88 – 1.80 (m, 2H), 1.65 (s, 6H), 0.96 (d, *J* = 8.1 Hz, 12H).

Table S2. Tabulated data from recycling of the superbase ionic liquid

Cycle	Initial SB-IL (g)	Initial nylon 6 (g)	Recovered SB-IL (g)	Recovered nylon 6 (g)
1	10.00	1.000	9.85	0.970
2	9.85	0.985	9.77	0.970
3	9.77	0.977	9.30	0.920
4	9.30	0.930	9.20	0.920
5	9.20	0.920	9.00	0.900

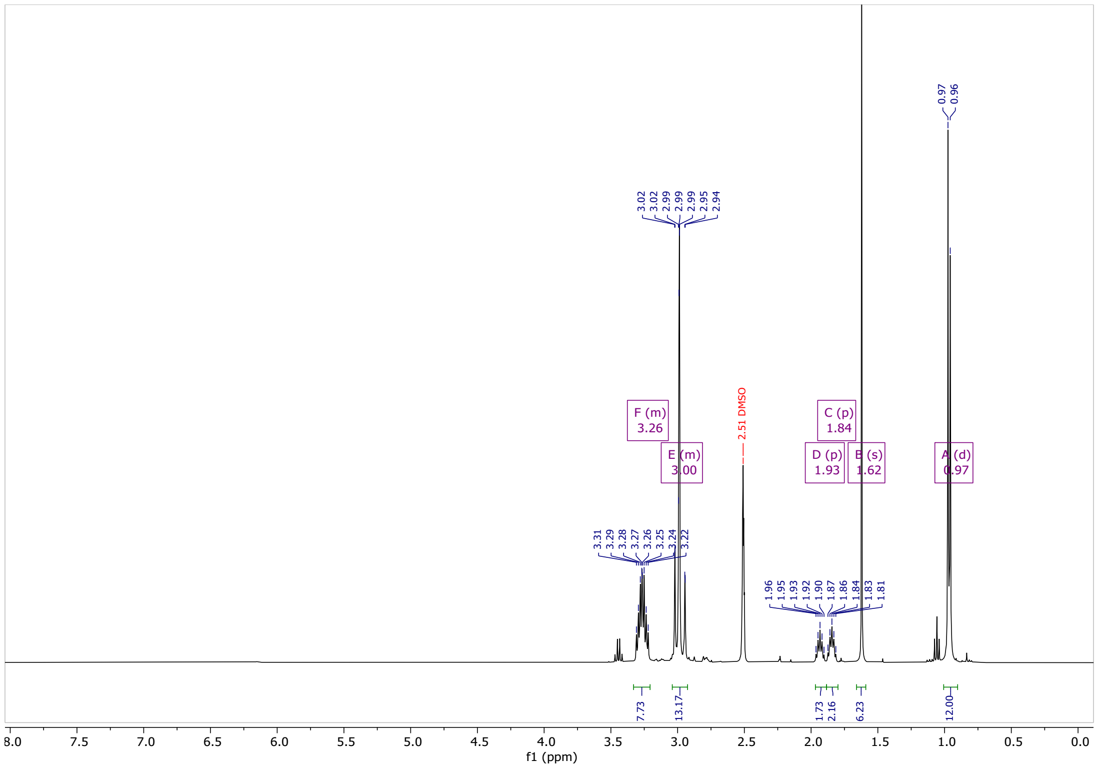

Figure S70 ^{1}H NMR spectra of [dm$_3$-mTBDH][OAc] after the 1st nylon 6 dissolution. Traces of residual ethanol are visible on the spectra.

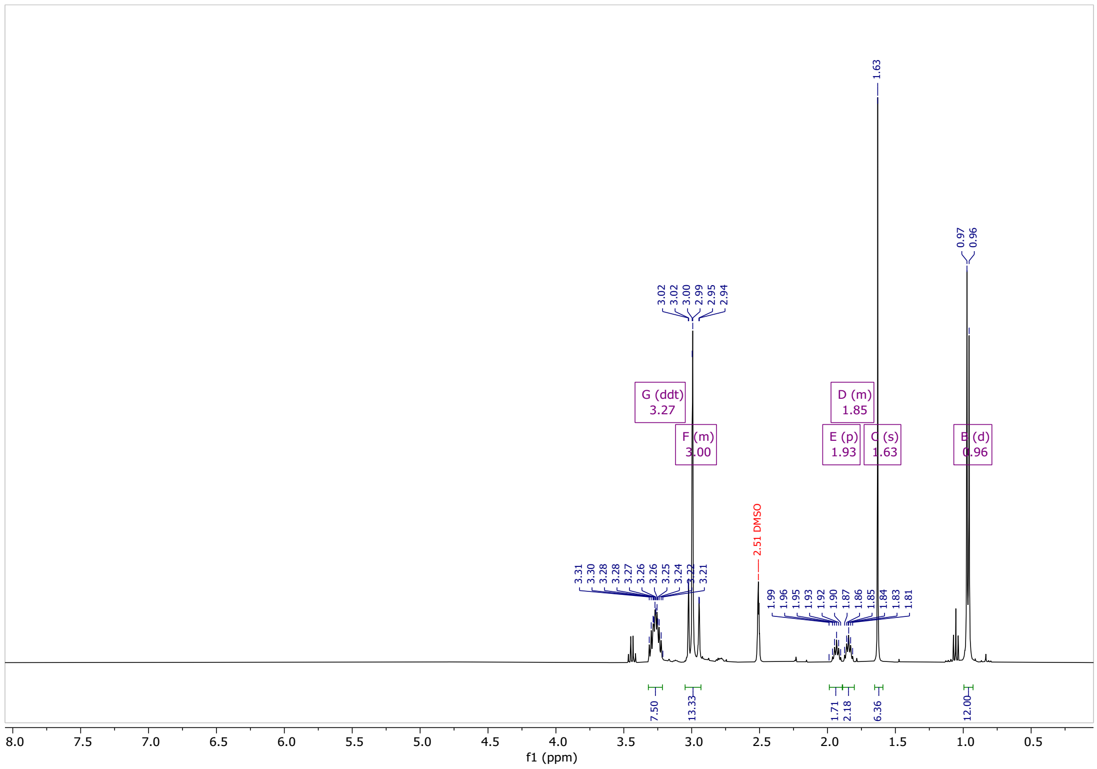

Figure S71 ^{1}H NMR spectra of [dm$_3$-mTBDH][OAc] after the 2nd nylon 6 dissolution. Traces of residual ethanol are visible on the spectra.

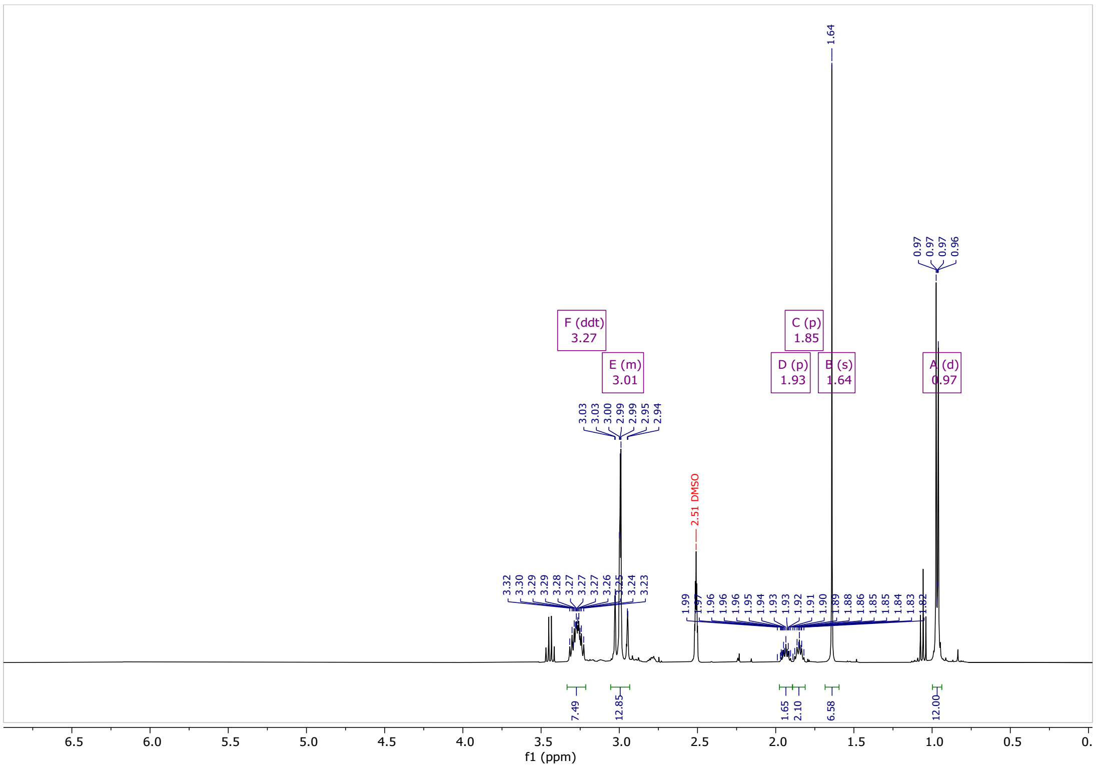

Figure S72 ¹H NMR spectra of [dm$_3$-mTBDH][OAc] after the 3rd nylon 6 dissolution. Traces of residual ethanol are visible on the spectra.

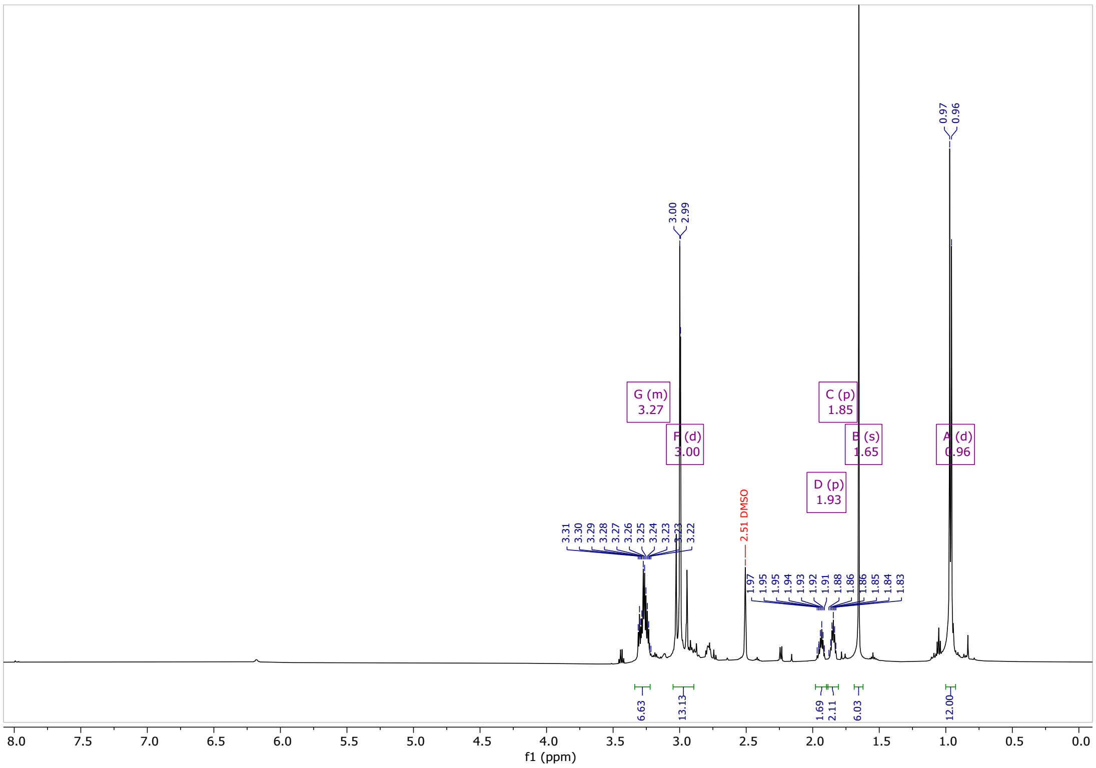

Figure S73 ¹H NMR spectra of [dm$_3$-mTBDH][OAc] after the 4th nylon 6 dissolution. Traces of residual ethanol are visible on the spectra.

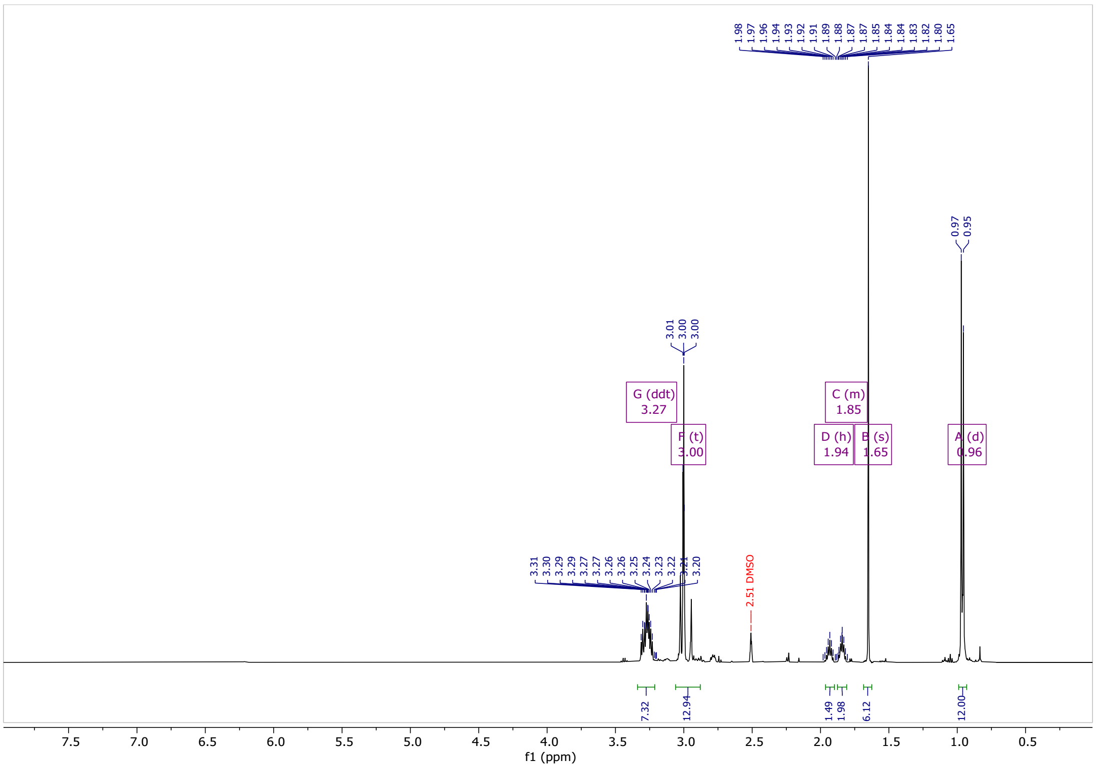

Figure S74 ^{1}H NMR spectra of [dm$_3$-mTBDH][OAc] after the 5th nylon 6 dissolution. Traces of residual ethanol are visible on the spectra.

Recycling of nylon 6 (three dissolution-regeneration cycles)

In a 100 mL round bottom flask equipped with an oval stirrer bar (20 x 10 mm), 1 g (10 % (by mass)) of nylon 6 and 10 g of [dm$_3$-mTBDH][OAc] or HFIP were charged. Then the suspended nylon sample was immersed into a preheated silicon oil bath (100 °C) for 20 hours. After that to the hot nylon solution was added 150 mL of EtOH in order to precipitate the nylon 6 material from the solutions and the heating was ceased. After that the mixture is stirred for an hour at room temperature and then the solids were filtered over a sintered funnel (Pore size 4) and were further transferred back into 150ml fresh ethanol. This step was repeated in total 3 times to wash the nylon as much as possible from the used solvent. After this, the separated nylon was dried under high vacuum at room temperature over 20 hours. Then a 0.1 g sample was taken for further analysis. After the drying and analysis, the same nylon sample was re-dissolved into a fresh batch of solvent. The dissolution and regeneration follow the same procedure as mentioned earlier observing the correct concentration (see table S3). Once the three regenerations cycle are over, the dried nylon can be stored in a sealed vial at room temperature for further use.

Note: *These experiments were done in small scale, which increases the possibility for experimental errors.*

NMR reports for [dm$_3$-mTBDH][OAc] recovered from the recycling experiments:

Cycle 1: **^{1}H NMR** (400 MHz, DMSO) δ 3.33 – 3.19 (m, 8H), 3.08 – 2.90 (m, 14H), 2.51 (s, 2H), 1.84 (p, *J* = 5.9 Hz, 2H), 1.62 (s, 6H), 0.97 (d, *J* = 6.8 Hz, 12H).

Cycle 2: **^{1}H NMR** (400 MHz, DMSO) δ 3.27 (ddt, *J* = 12.1, 10.0, 5.9 Hz, 8H), 3.06 – 2.92 (m, 14H), 1.93 (p, *J* = 6.0 Hz, 2H), 1.84 (p, *J* = 5.9 Hz, 2H), 1.63 (s, 6H), 0.96 (d, *J* = 6.4 Hz, 12H).

Cycle 3: **^{1}H NMR** (400 MHz, DMSO) δ 3.27 (ddt, *J* = 12.5, 9.5, 6.0 Hz, 8H), 2.99 (d, *J* = 2.0 Hz, 14H), 1.93 (p, *J* = 6.1 Hz, 2H), 1.88 – 1.82 (m, 2H), 1.64 (s, 6H), 0.97 (d, *J* = 5.6 Hz, 12H).

NMR reports for recovered Nylon 6 from [dm$_3$-mTBDH][OAc]:

Cycle 1: **^{1}H NMR** (600 MHz, DMSO/HFIP) δ 6.20 (s, 1H), 3.14 (q, *J* = 6.8 Hz, 2H), 2.13 (t, *J* = 7.9 Hz, 2H), 1.54 (p, *J* = 7.9 Hz, 2H), 1.45 (p, *J* = 7.6 Hz, 2H), 1.26 (p, *J* = 7.9 Hz, 2H).

Cycle 2: **^{1}H NMR** (600 MHz, DMSO/HFIP) δ 6.13 (s, 1H), 3.14 (q, *J* = 6.8 Hz, 2H), 2.13 (t, *J* = 8.2 Hz, 2H), 1.53 (p, *J* = 7.4 Hz, 2H), 1.45 (p, *J* = 7.6 Hz, 2H), 1.26 (p, *J* = 7.9 Hz, 2H).

Cycle 3: **^{1}H NMR** (600 MHz, DMSO/HFIP) δ 5.83 (s, 1H), 2.84 (q, *J* = 7.4 Hz, 2H), 1.83 (s, 2H), 1.23 (q, *J* = 8.3 Hz, 2H), 1.15 (q, *J* = 7.5 Hz, 2H), 1.01 – 0.93 (m, 2H).

NMR reports for recovered Nylon 6 from HFIP:

Cycle 1: **^{1}H NMR** (600 MHz, DMSO/HFIP) δ 6.20 (s, 1H), 3.14 (q, *J* = 6.8 Hz, 2H), 2.13 (t, *J* = 7.9 Hz, 2H), 1.54 (p, *J* = 7.9 Hz, 2H), 1.45 (p, *J* = 7.6 Hz, 2H), 1.26 (p, *J* = 7.9 Hz, 2H).

Cycle 2: **^{1}H NMR** (600 MHz, DMSO/HFIP) δ 6.13 (s, 1H), 3.14 (q, *J* = 6.9 Hz, 2H), 2.22 – 2.07 (m, 2H), 1.53 (p, *J* = 7.3 Hz, 2H), 1.45 (p, *J* = 7.6 Hz, 2H), 1.26 (p, *J* = 7.9 Hz, 2H).

Cycle 3: **^{1}H NMR** (600 MHz, DMSO/HFIP) δ 5.83 (s, 1H), 2.84 (s, 2H), 1.83 (q, *J* = 8.4 Hz, 2H), 1.23 (q, *J* = 8.3 Hz, 2H), 1.15 (q, *J* = 7.5 Hz, 2H), 0.97 (p, *J* = 8.4 Hz, 2H).

Table S3. Tabulated data from dissolution-regeneration cycles of nylon 6

Cycle	Initial SB-IL (g)	Initial nylon 6 (g)	Recovered SB-IL (g)	Recovered nylon 6 (g)
Recovery from [dm_3-mTBDH][OAc]				
1	10.00	1.00	9.50	0.95
2	8.50	0.85	7.94	0.70
3	6.00	0.60	5.85	0.57
Recovery from HFIP				
1	10.00	1.00	-	0.95
2	8.50	0.85	-	0.79
3	6.00	0.60	-	0.51

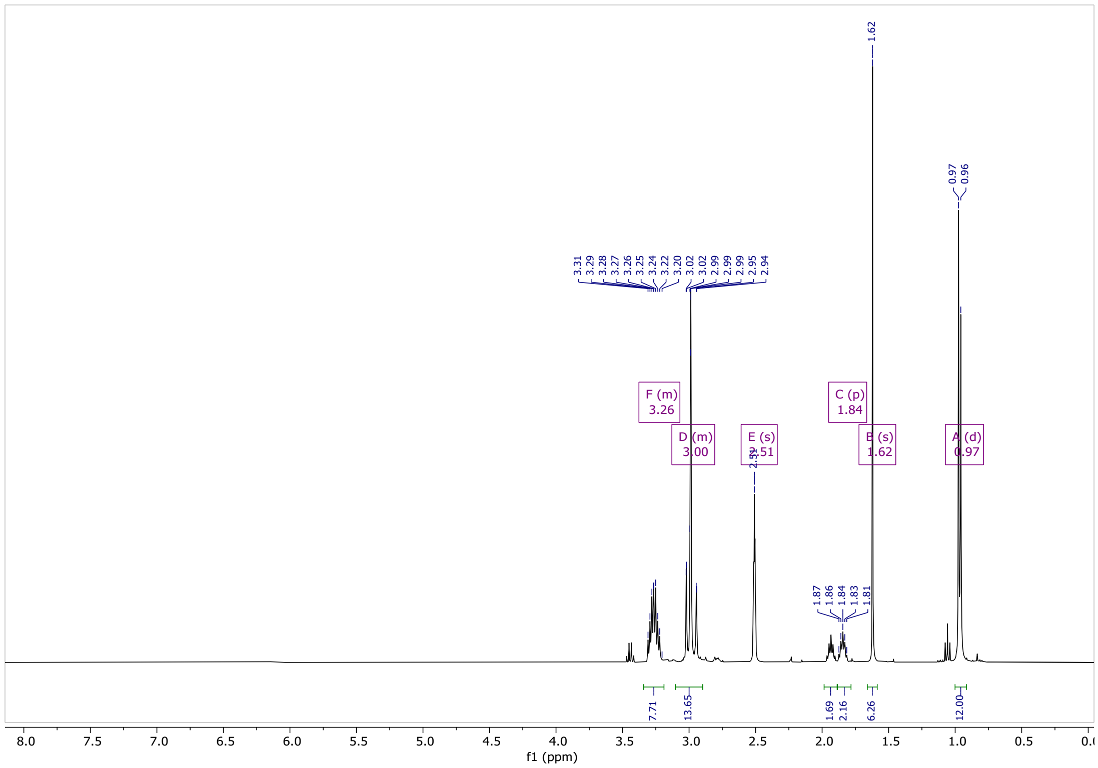

Figure S75 ^{1}H NMR spectra of [dm_3-mTBDH][OAc] after the 1st nylon 6 dissolution-regeneration cycle. Traces of residual ethanol are visible on the spectra.

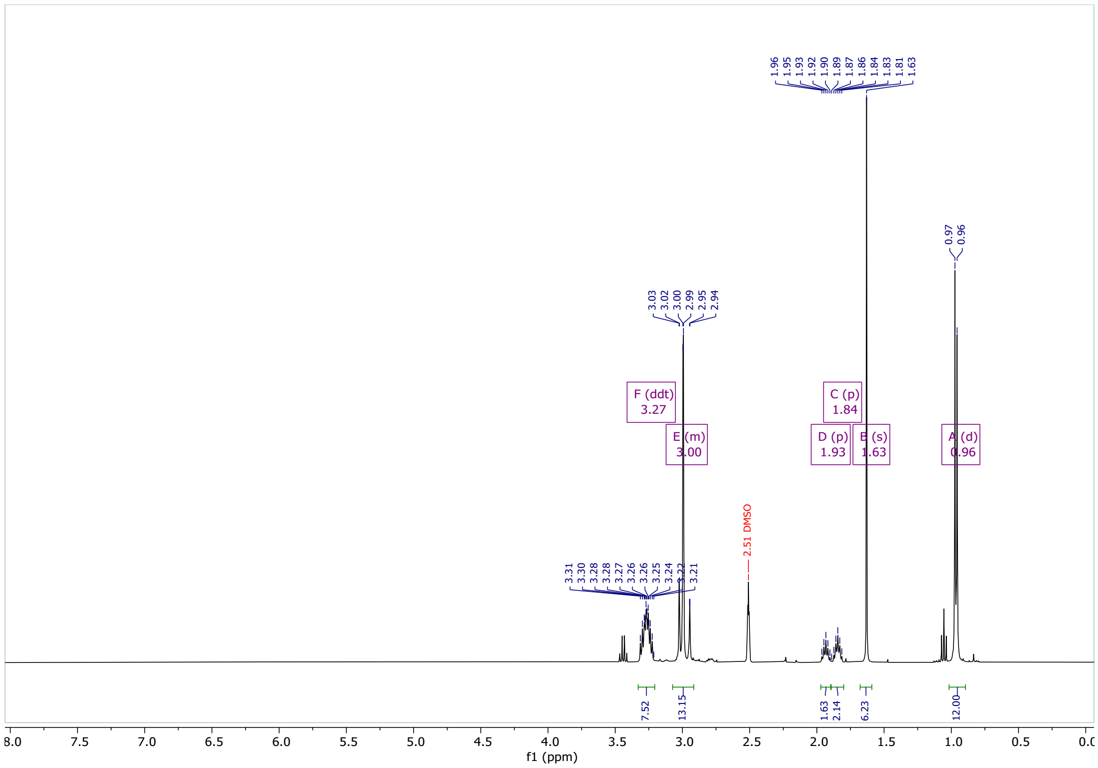

Figure S76 ^1H NMR spectra of [dm$_3$-mTBDH][OAc] after the 2nd nylon 6 dissolution-regeneration cycle.

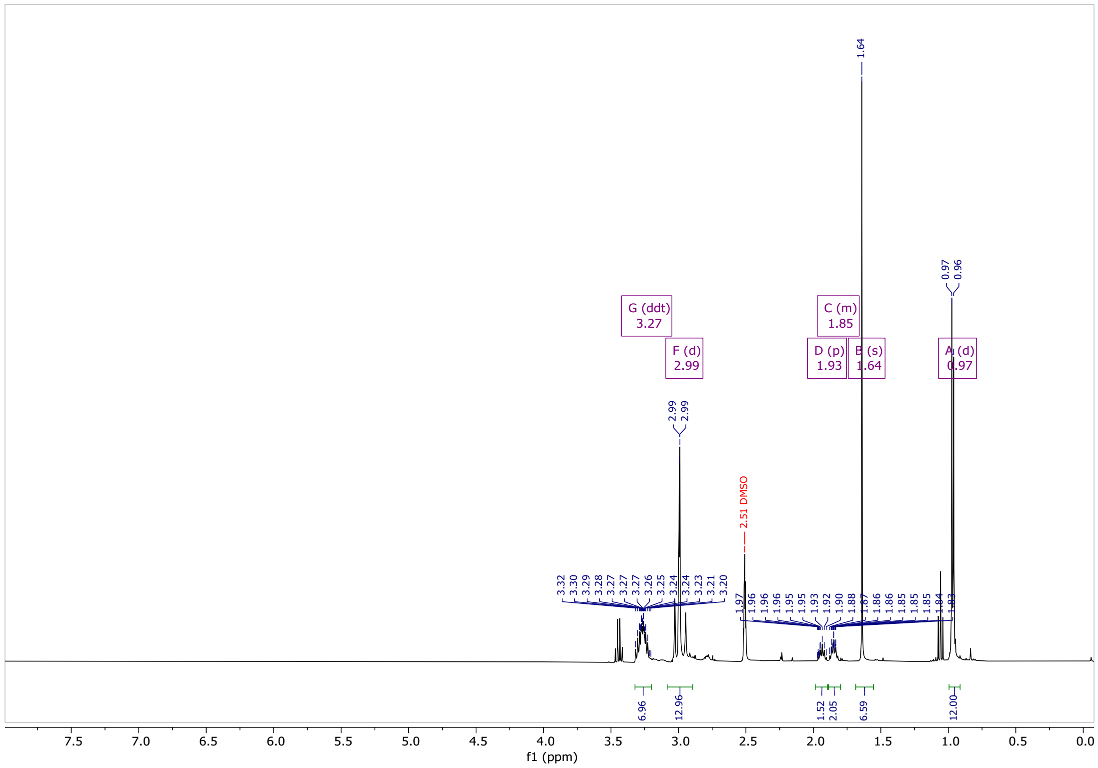

Figure S77 ^1H NMR spectra of [dm$_3$-mTBDH][OAc] after the 3rd nylon 6 dissolution-regeneration cycle. Traces of residual ethanol are visible on the spectra.

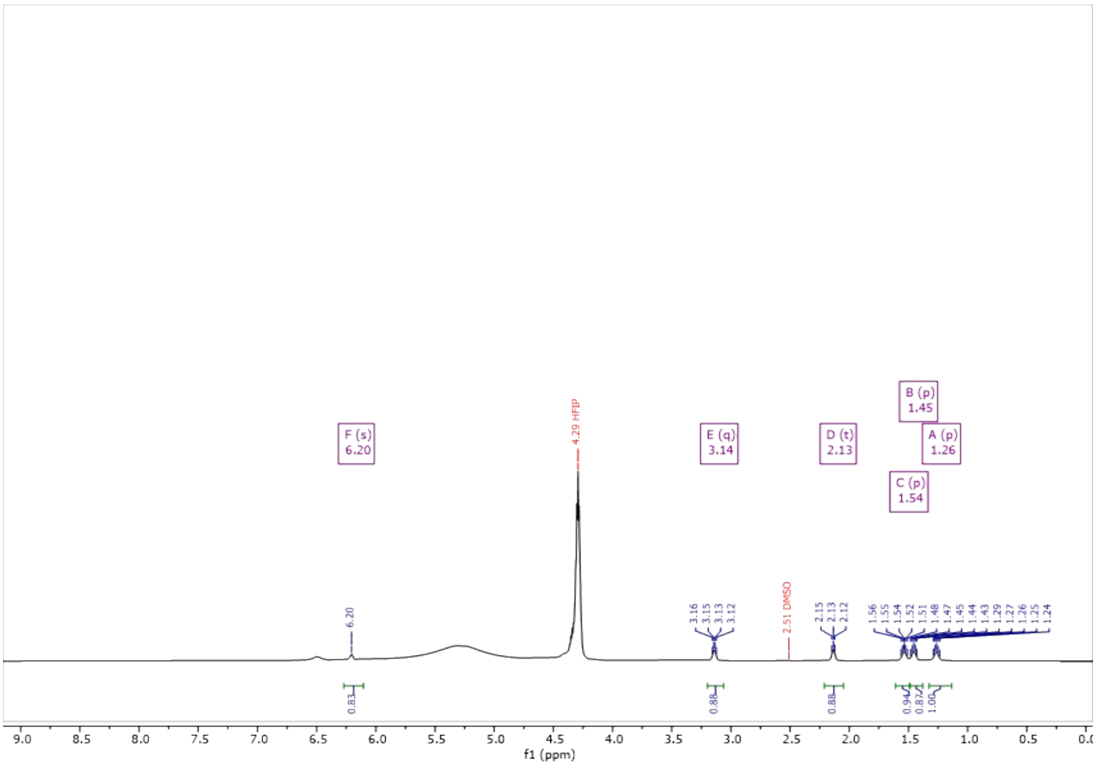

Figure S78 ¹H NMR spectra of Nylon 6 regenerated from [dm₃-mTBDH][OAc] cycle 1

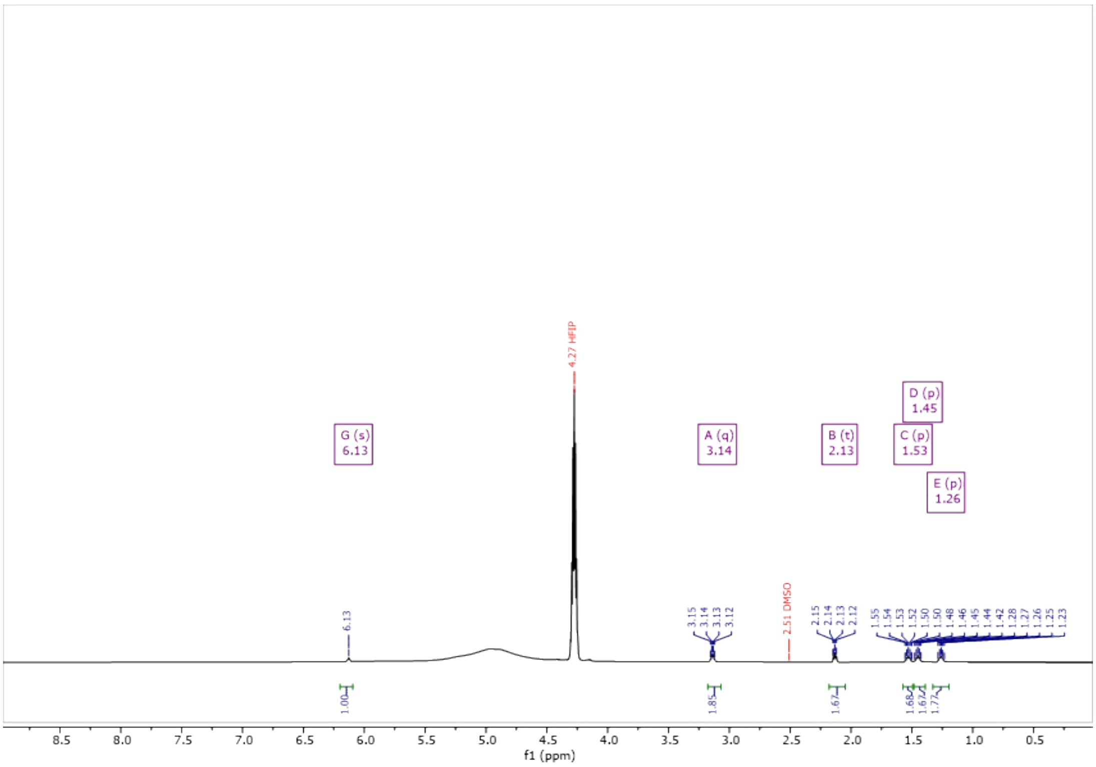

Figure S79 ¹H NMR spectra of Nylon 6 regenerated from [dm₃-mTBDH][OAc] cycle 2

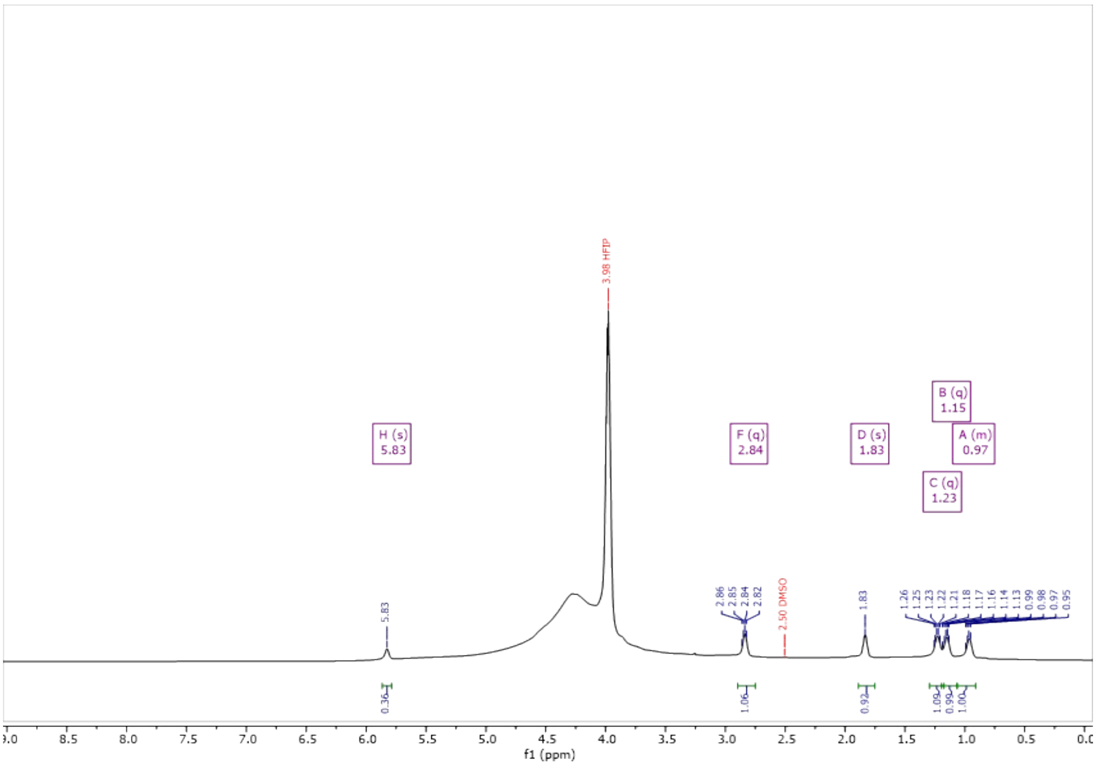

Figure S80 ^{1}H NMR spectra of Nylon 6 regenerated from [dm$_3$-mTBDH][OAc] cycle 3

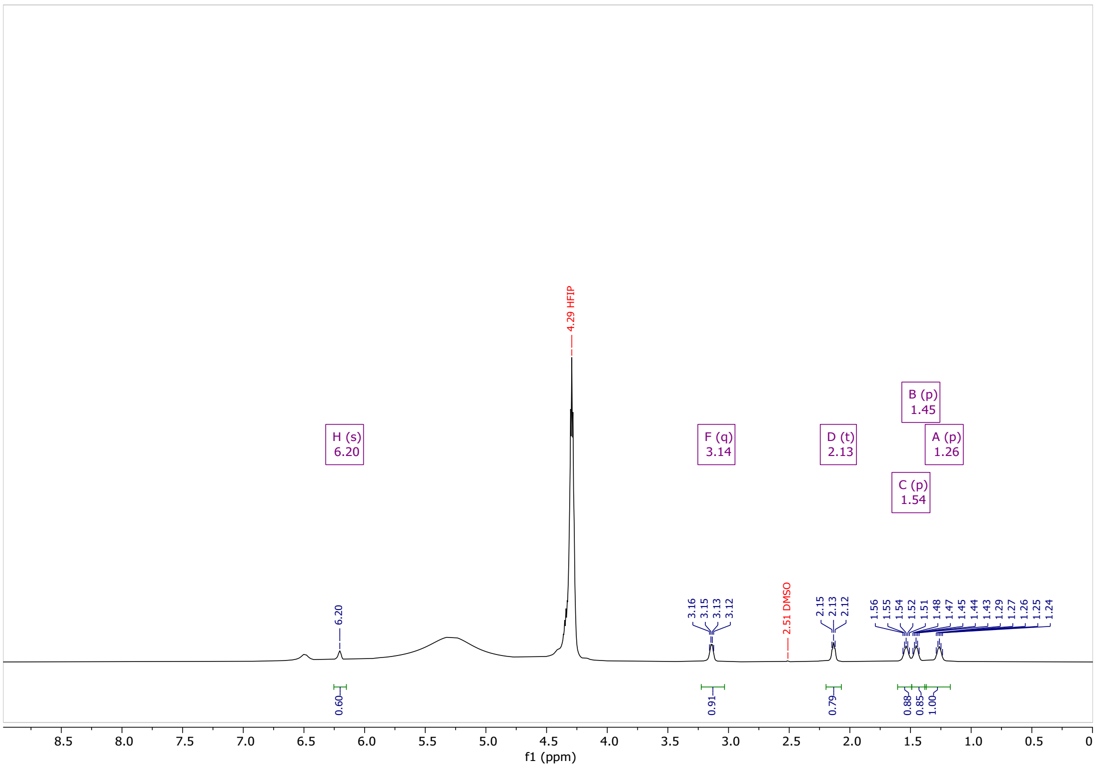

Figure S81 ^{1}H NMR spectra of Nylon 6 regenerated from HFIP cycle 1

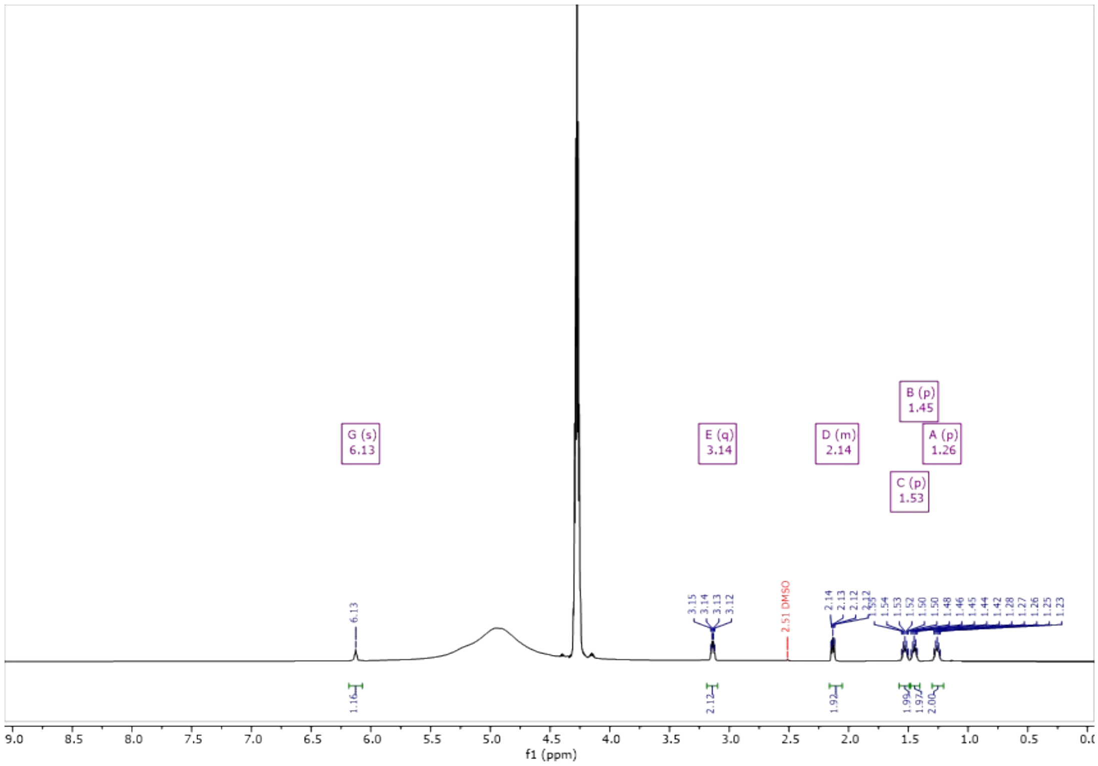

Figure S82 ^{1}H NMR spectra of Nylon 6 regenerated from HFIP cycle 2

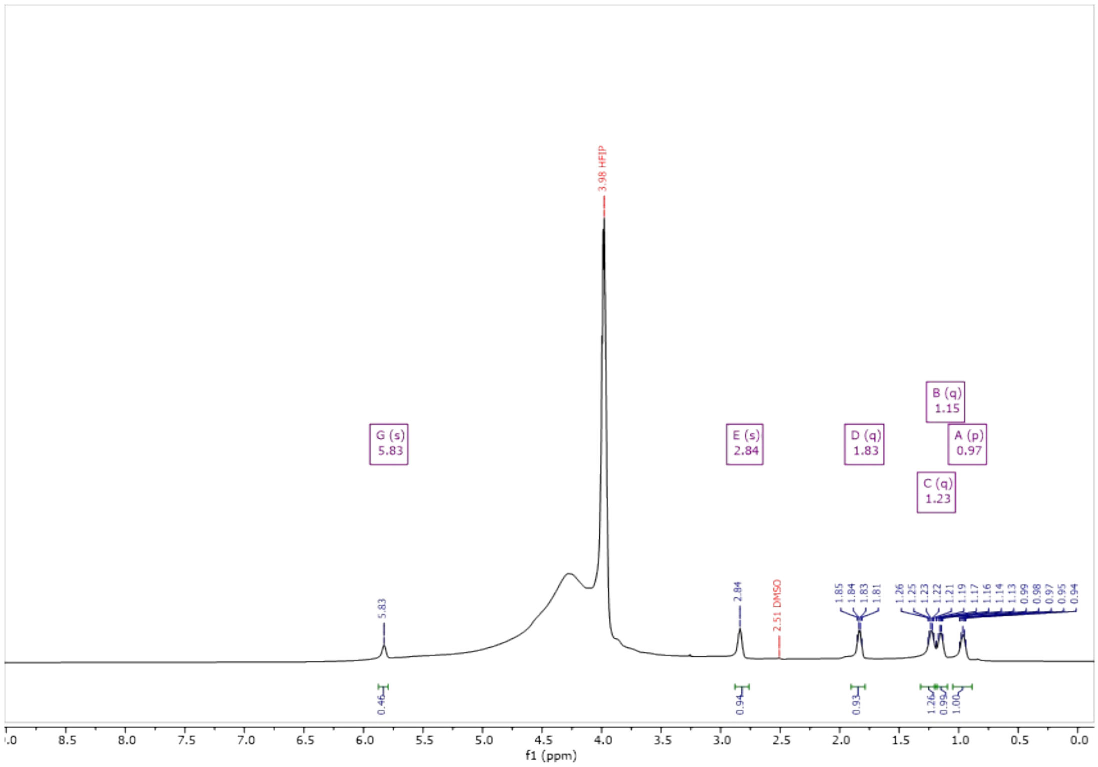

Figure S83 ^{1}H NMR spectra of Nylon 6 regenerated from HFIP cycle 3

NMR spectra of nylons

a. Characterization of virgin nylon 6

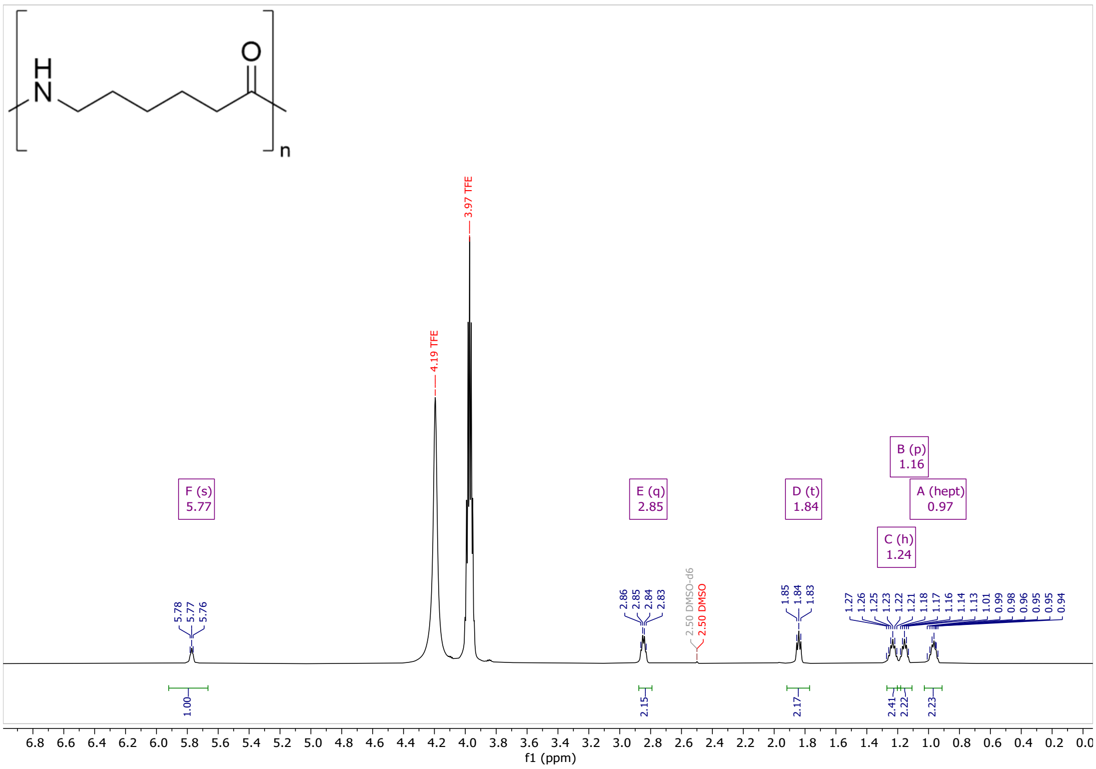

Figure S84 ¹H spectra of nylon 6 in HFIP

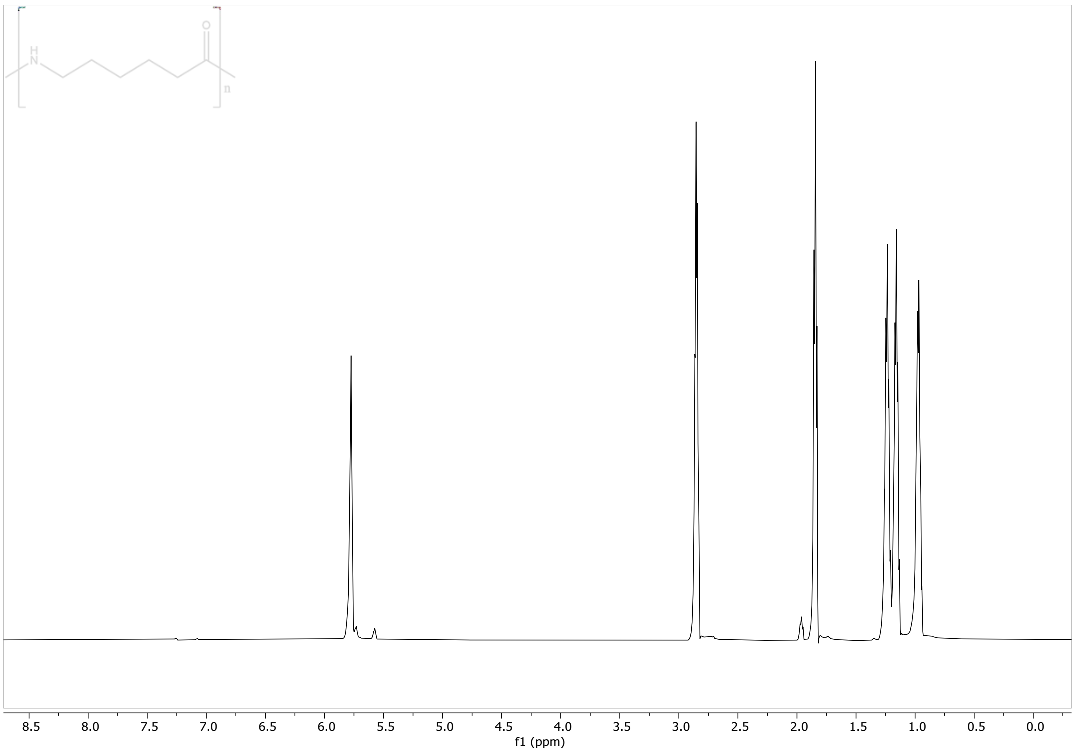

Figure S85 ¹H diffusion-edited of nylon 6 in HFIP

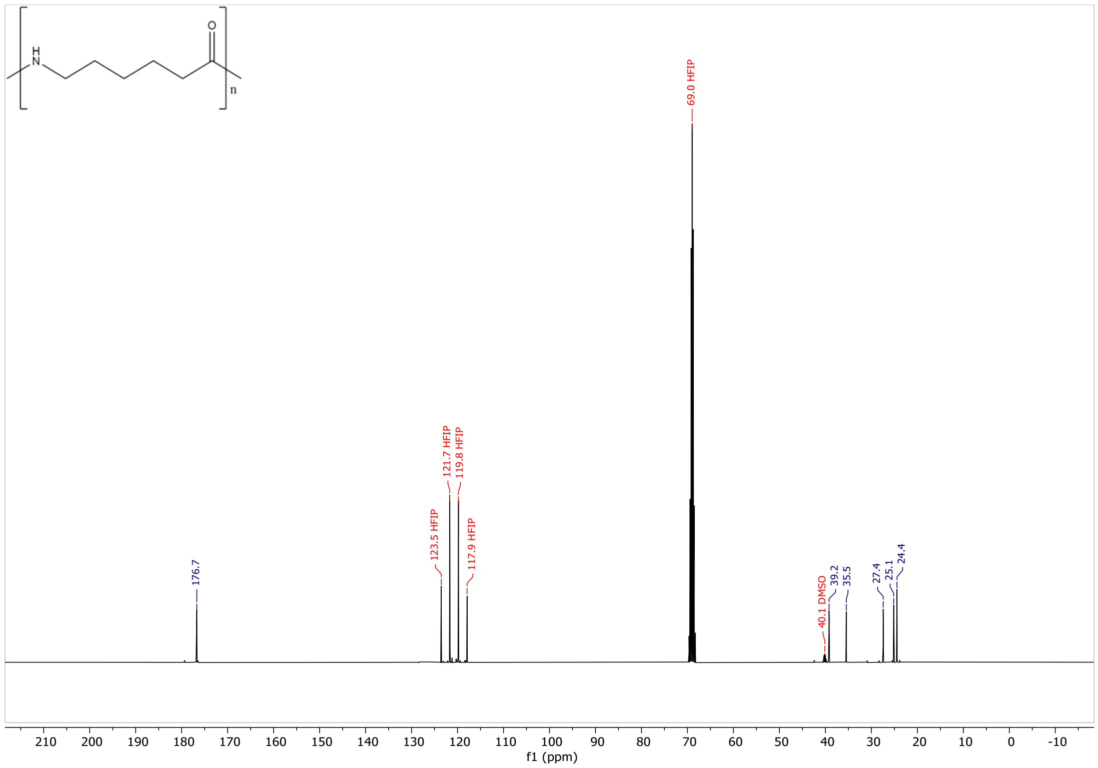

Figure S86 ^{13}C spectra of nylon 6 in HFIP

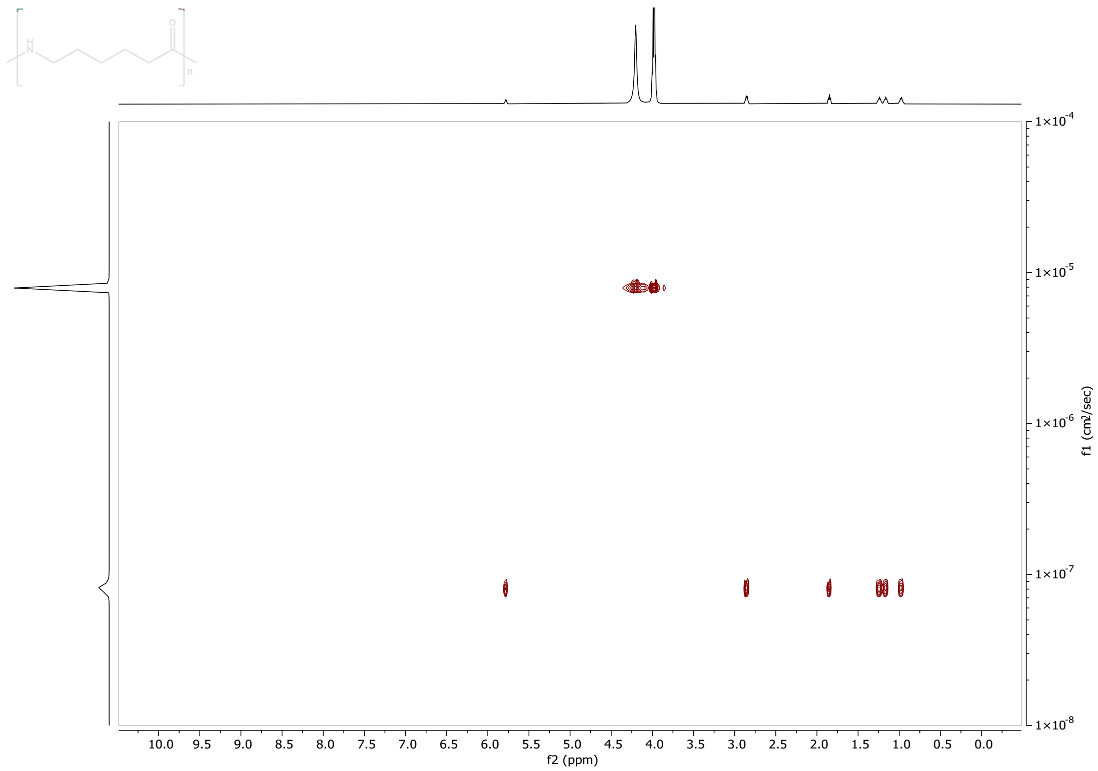

Figure S87 DOSY of nylon 6 in HFIP

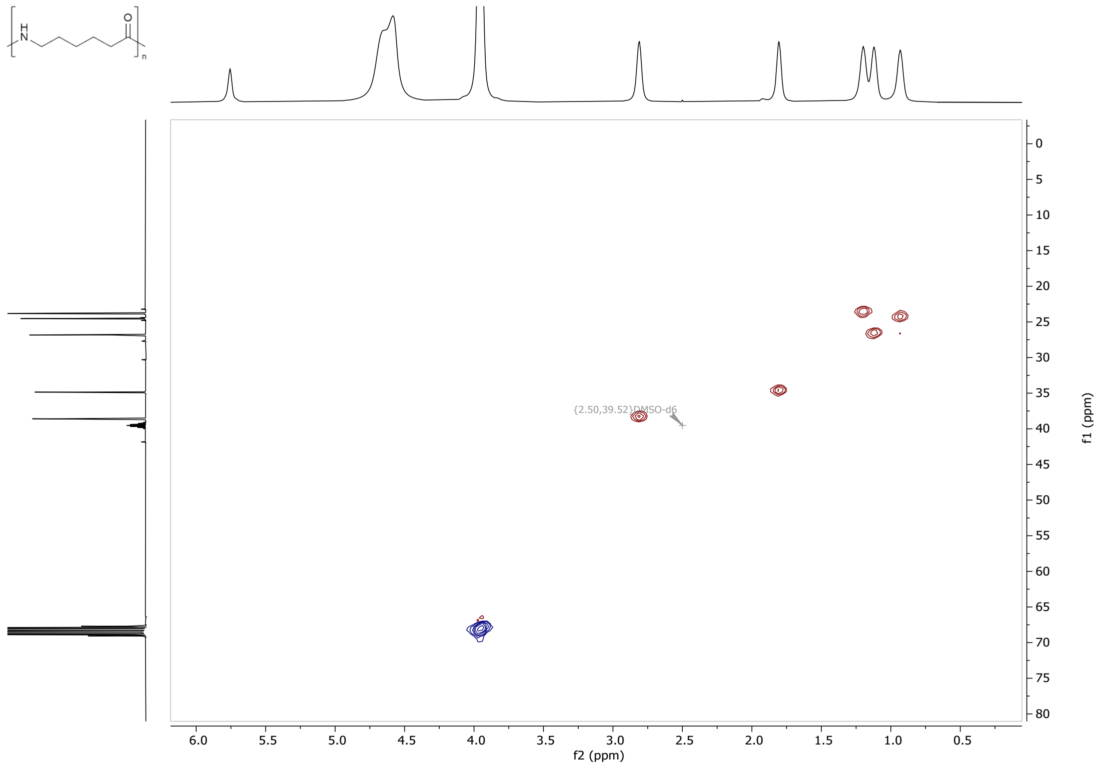

Figure S88 HSQC of nylon 6 in HFIP

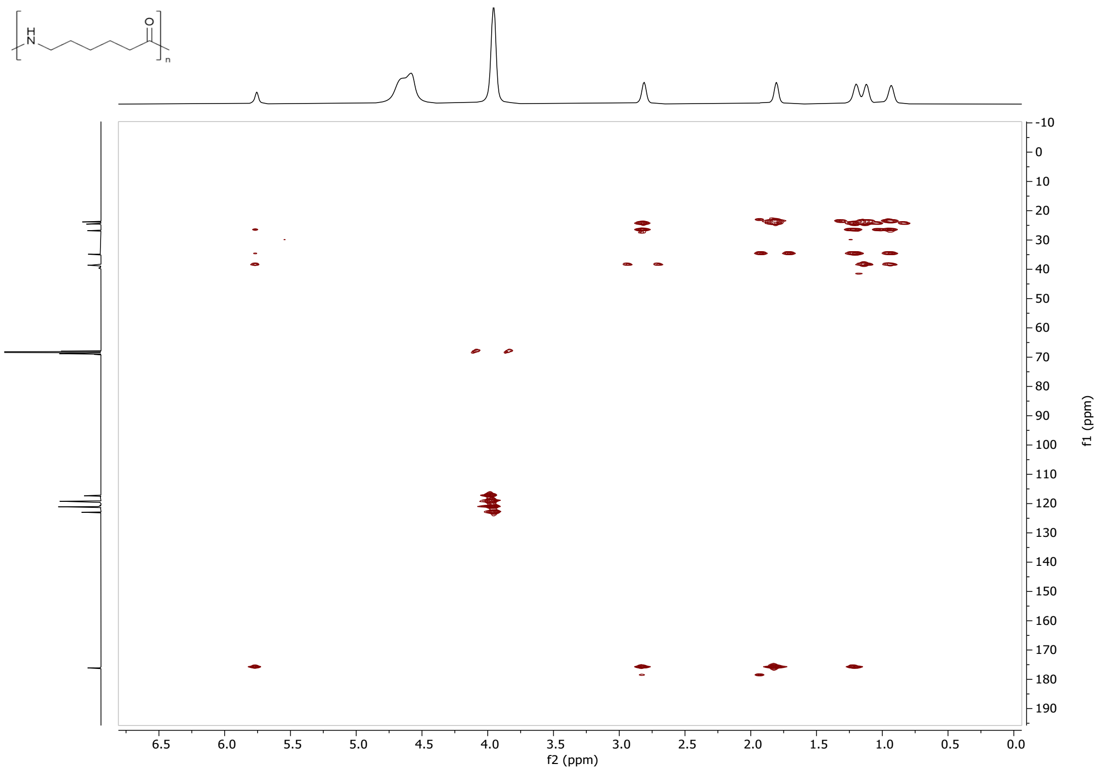

Figure S89 HMBC of nylon 6

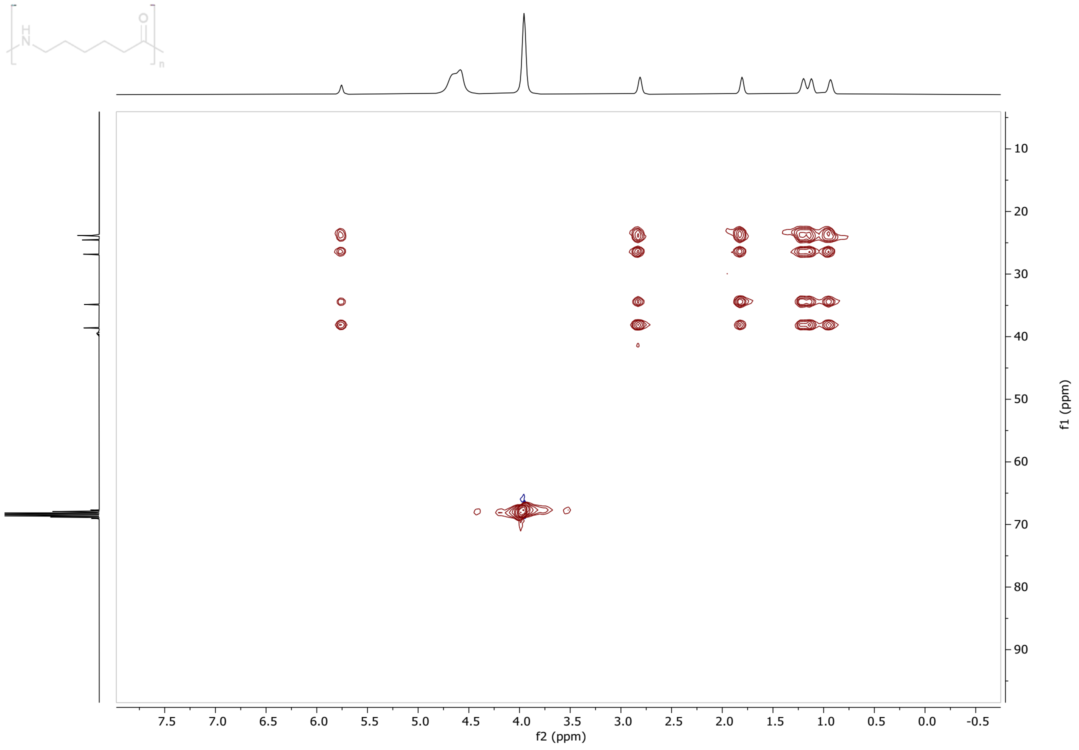

Figure S90 HSQC-TOCSY, (120 ms) of nylon 6 in HFIP

b. Characterization of virgin nylon 6,6 (P)

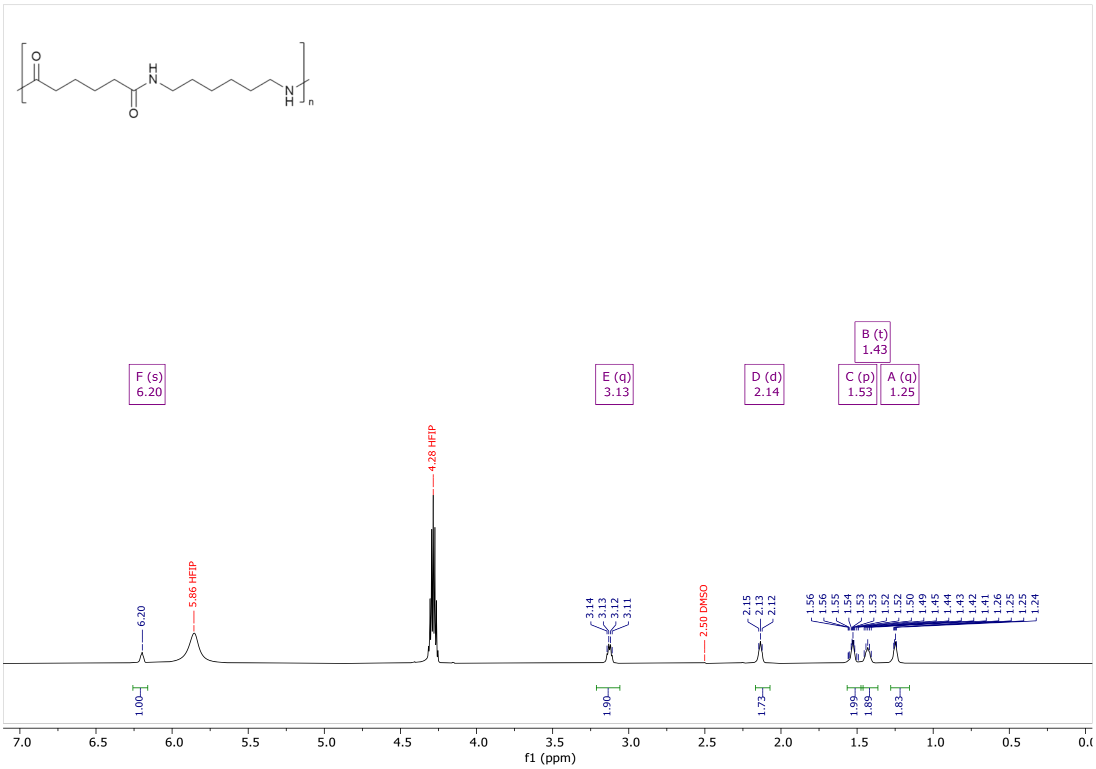

Figure S91 ^{1}H spectra of nylon 6,6 (P) in HFIP

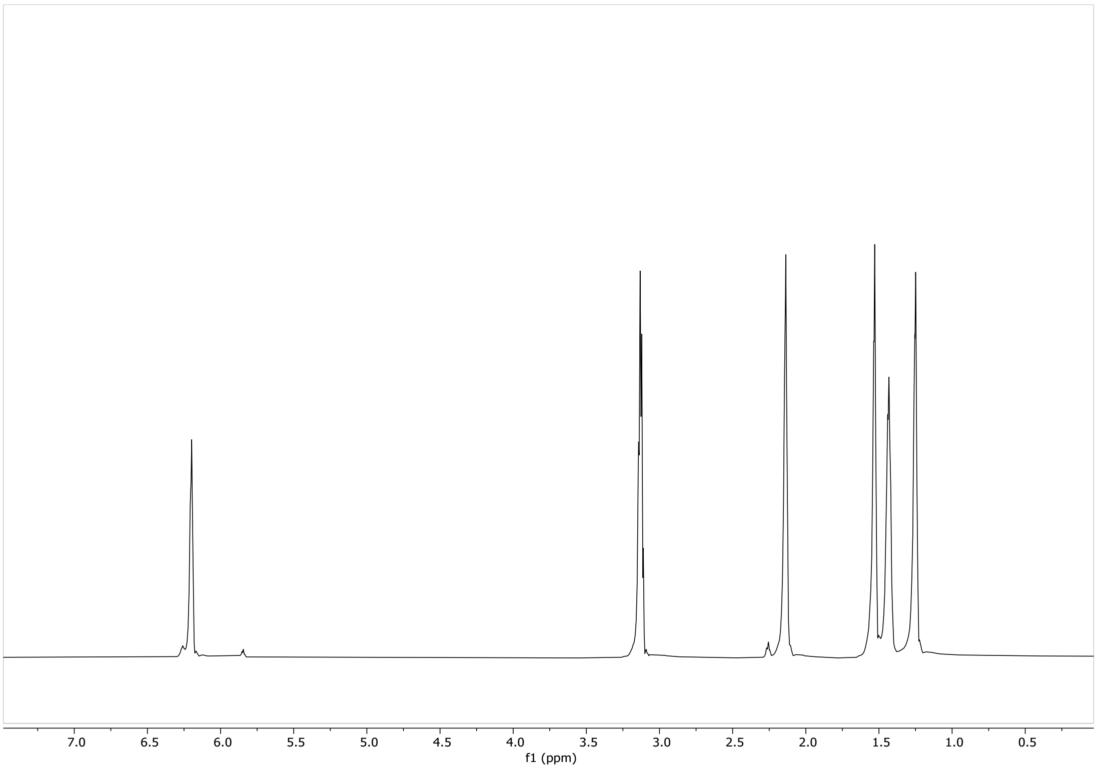

Figure S92 ¹H diffusion-edited spectra of nylon 6,6 (P) in HFIP

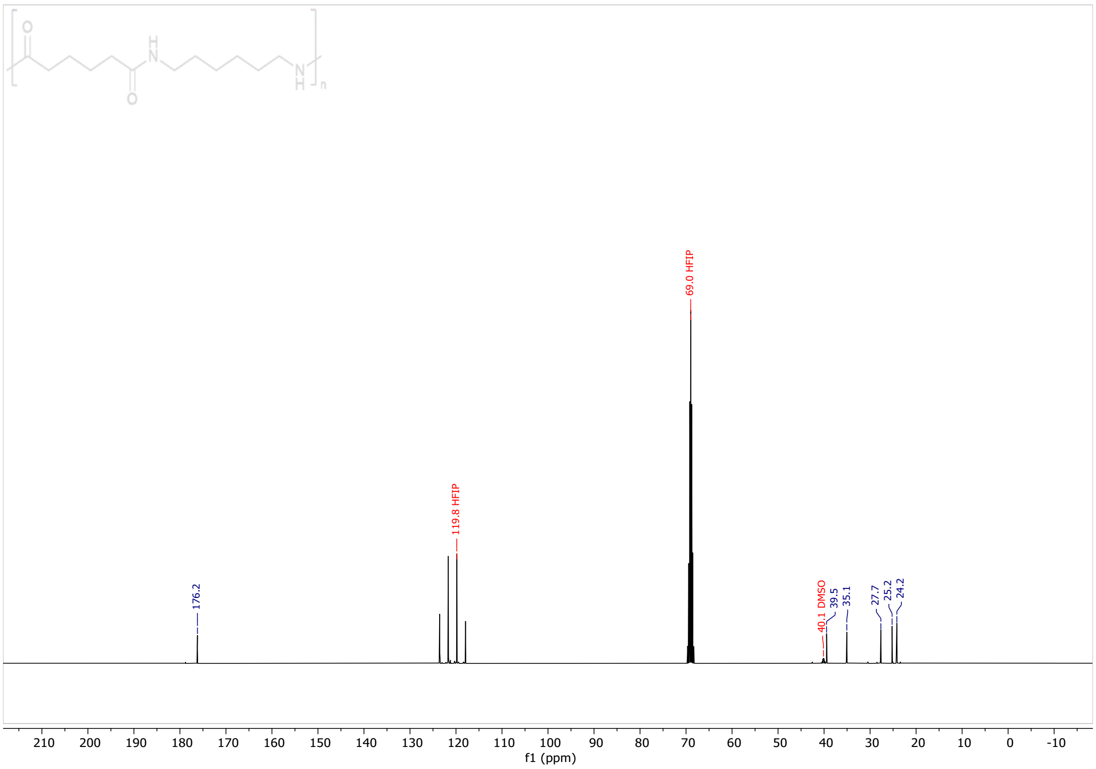

Figure S93 ¹³C spectra of nylon 6,6 (P) in HFIP

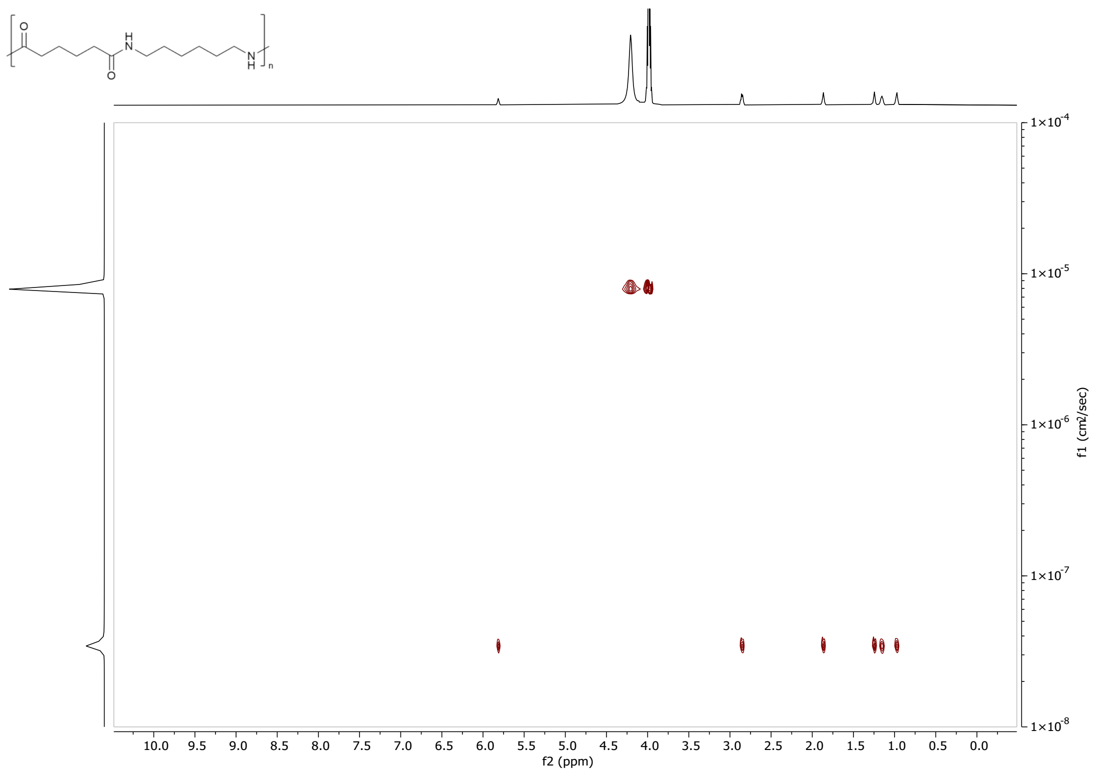

Figure S94 DOSY spectra of nylon 6,6 (P)

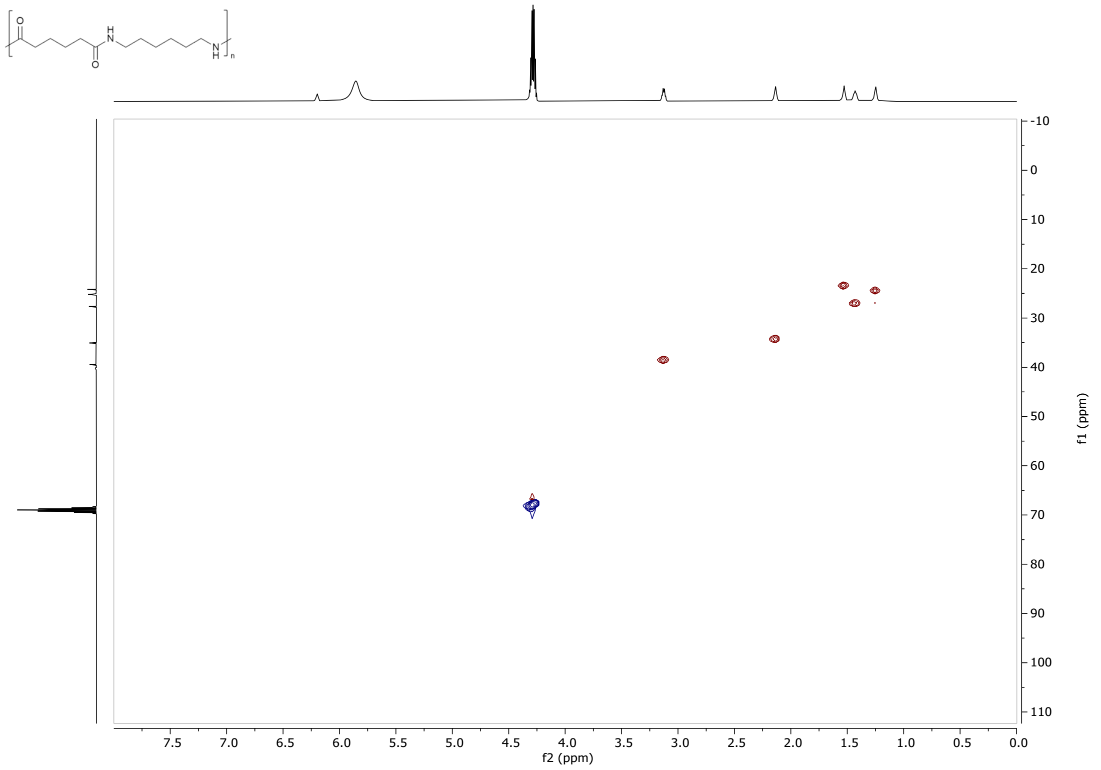

Figure S95 HSQC of nylon 6,6 (P) in HFIP

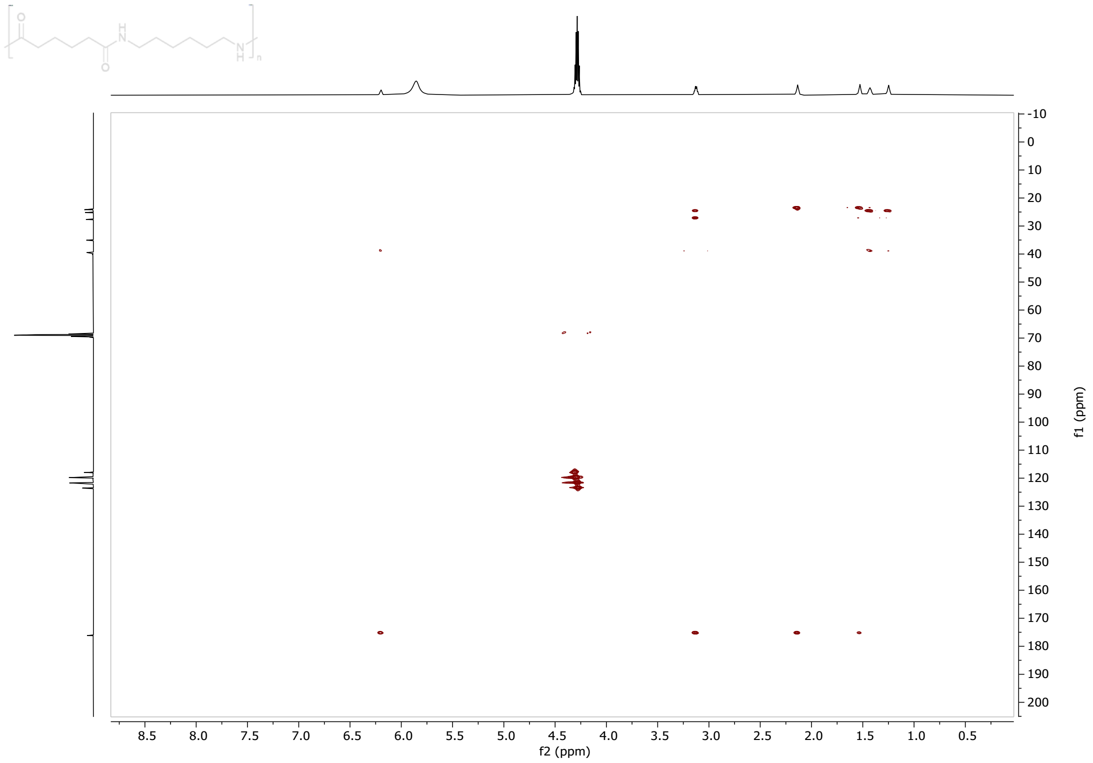

Figure S96 HMBC of nylon 6,6 (P) in HFIP

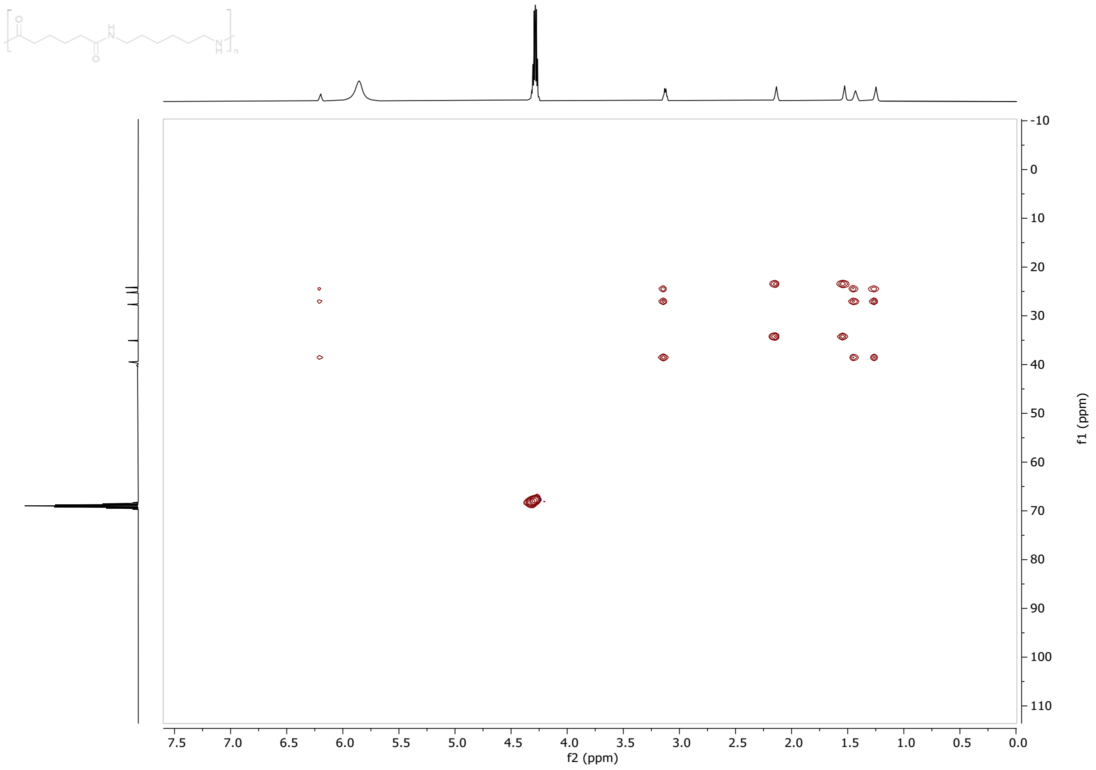

Figure S97 HSQC-TOCSY, (120 ms) of nylon 6,6 (P) in HFIP

c. Characterization of virgin 6,6 (M)

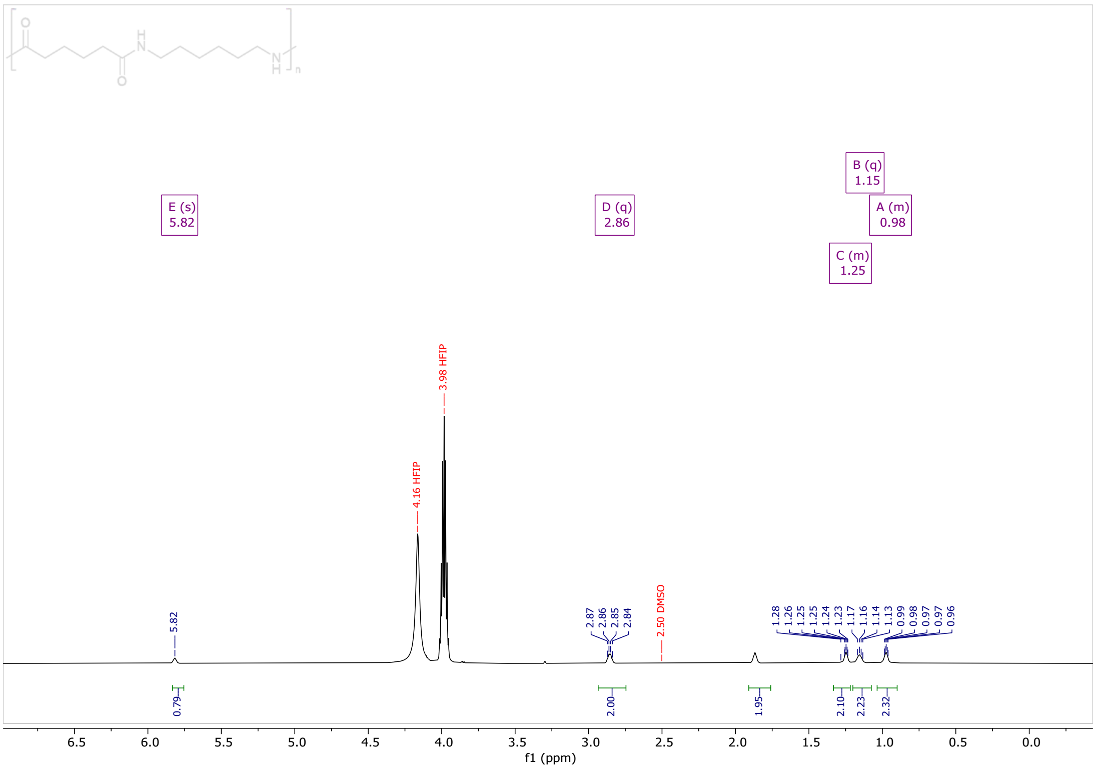

Figure S98 ^{1}H spectra of nylon 6,6 (M) in HFIP

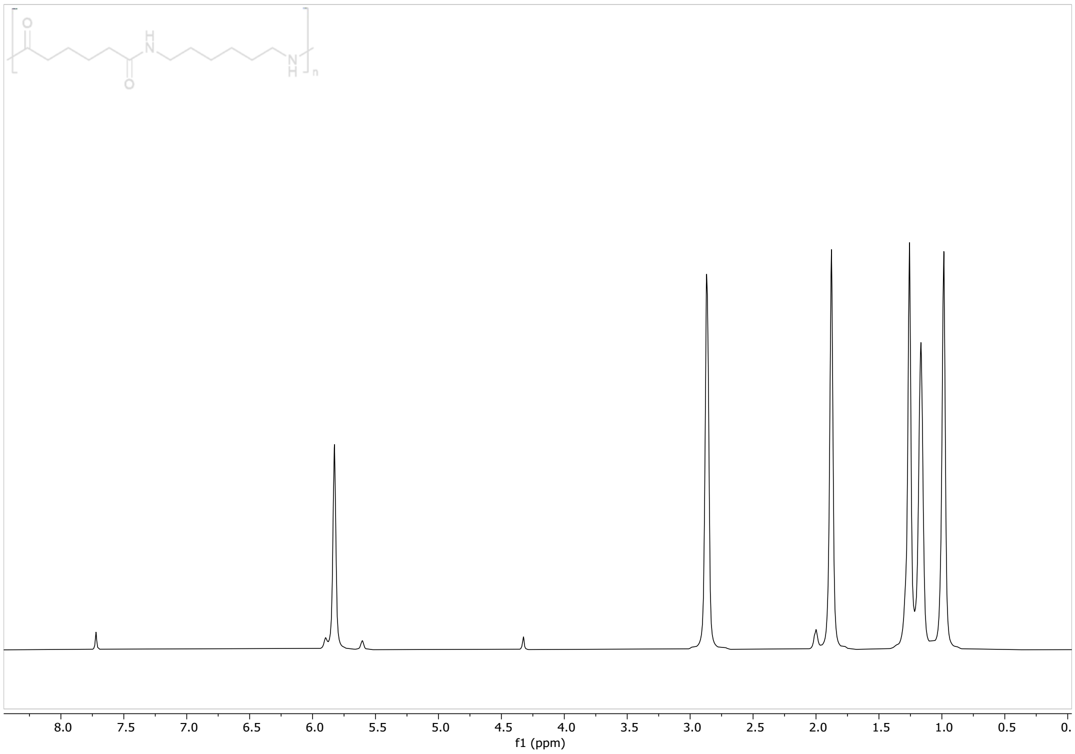

Figure S99 ^{1}H diffusion-edited spectra of nylon 6,6 (M) in HFIP

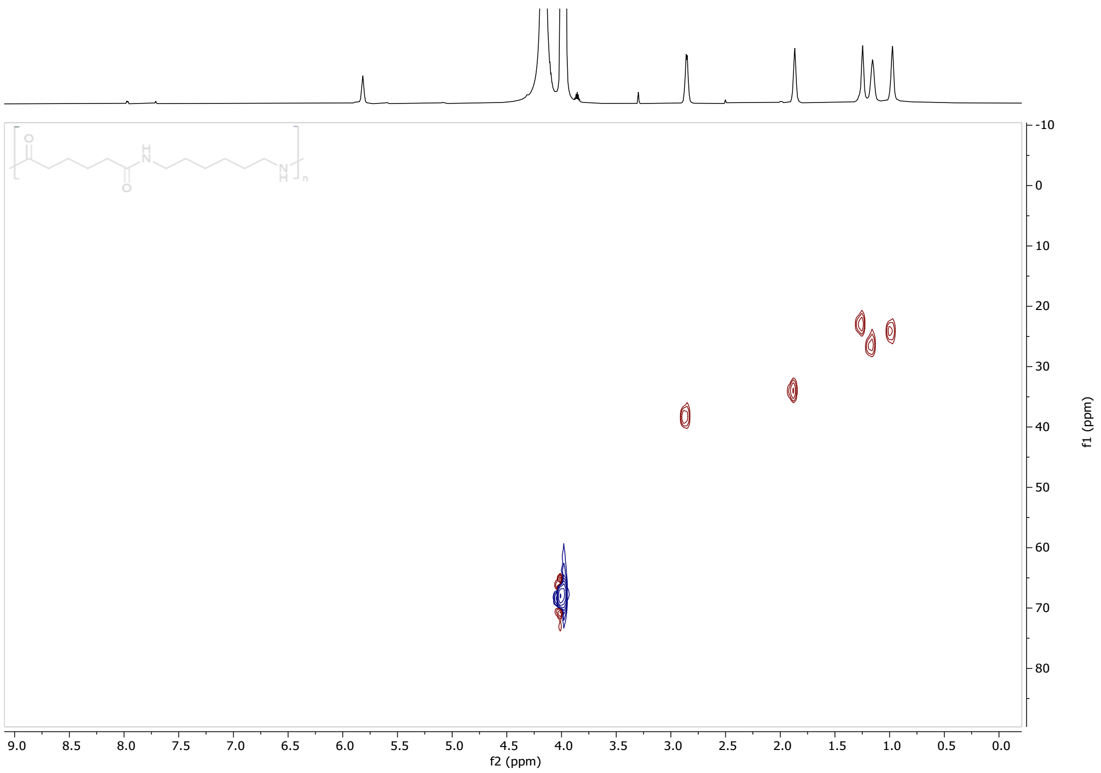

Figure S100 HSQC of nylon 6,6 (M) in HFIP

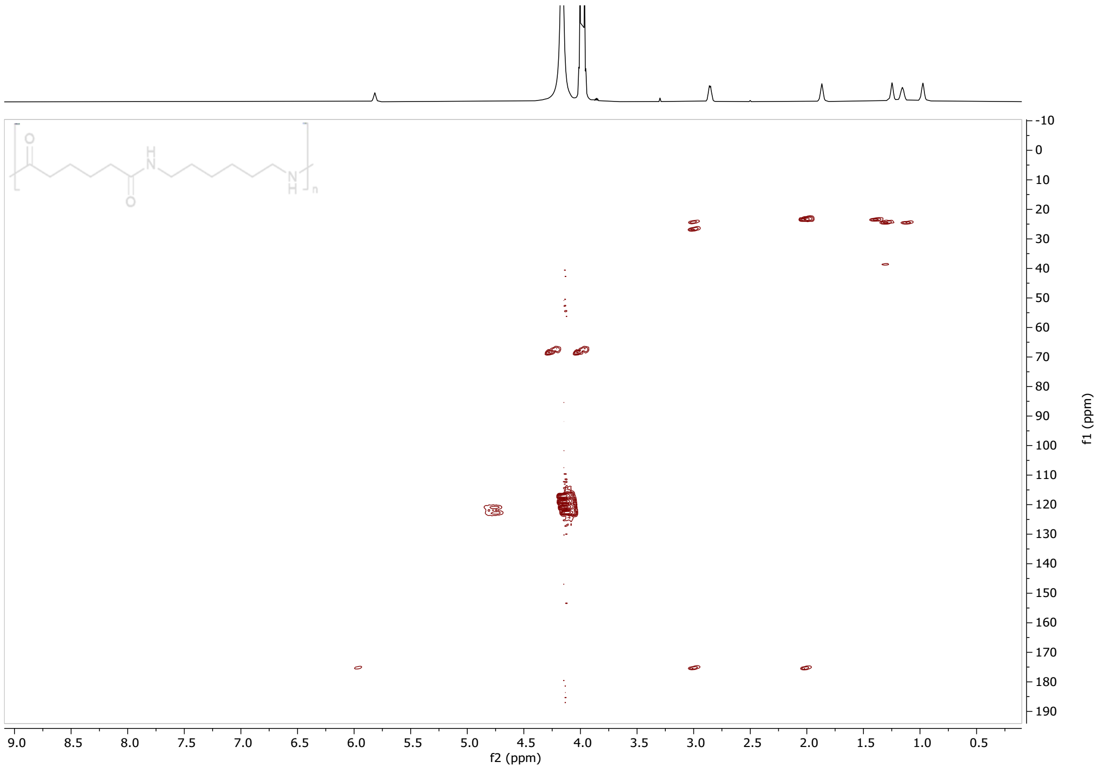

Figure S101 HMBC of nylon 6,6 (M) in HFIP

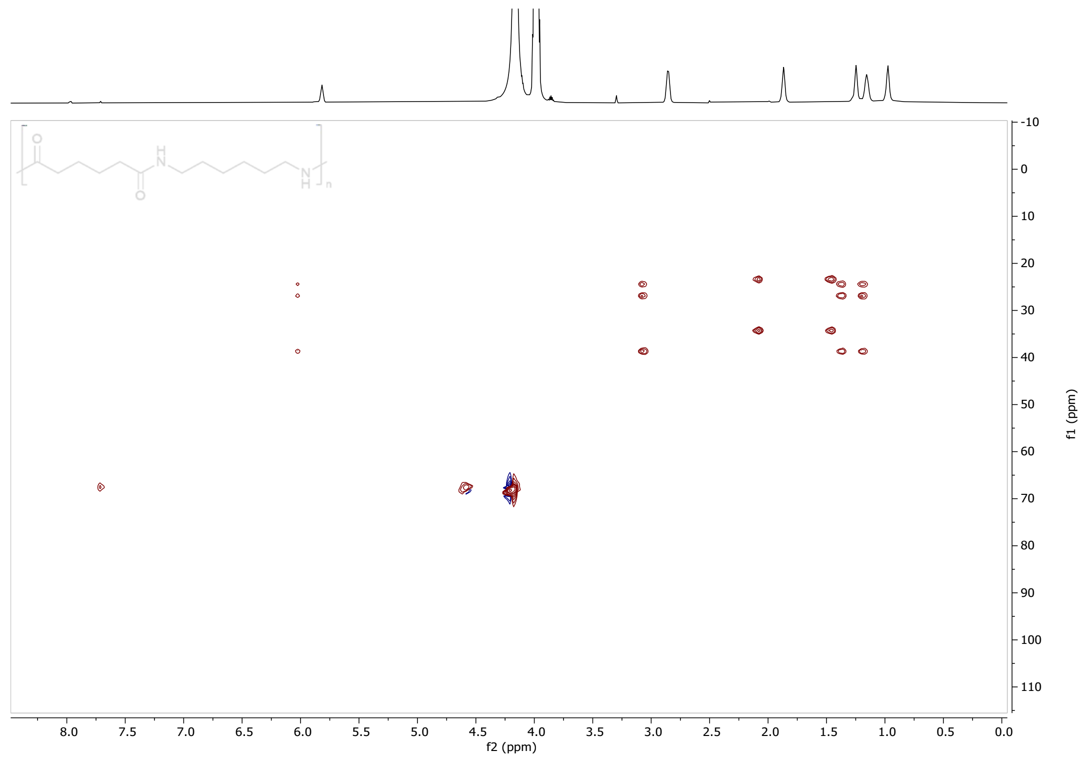

Figure S102 HSQC-TOCSY, (120 ms) of nylon 6,6 (P) in HFIP

d. Characterization of virgin nylon 6,12

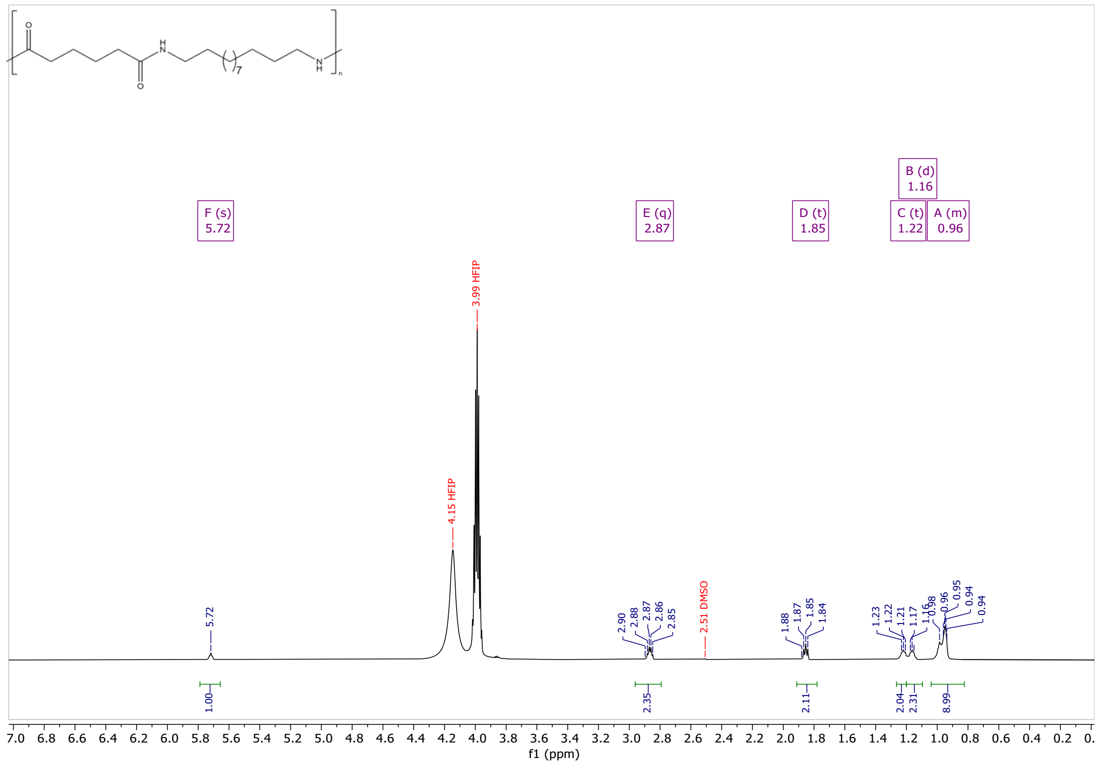

Figure S103 ^{1}H of nylon 6,12 in HFIP

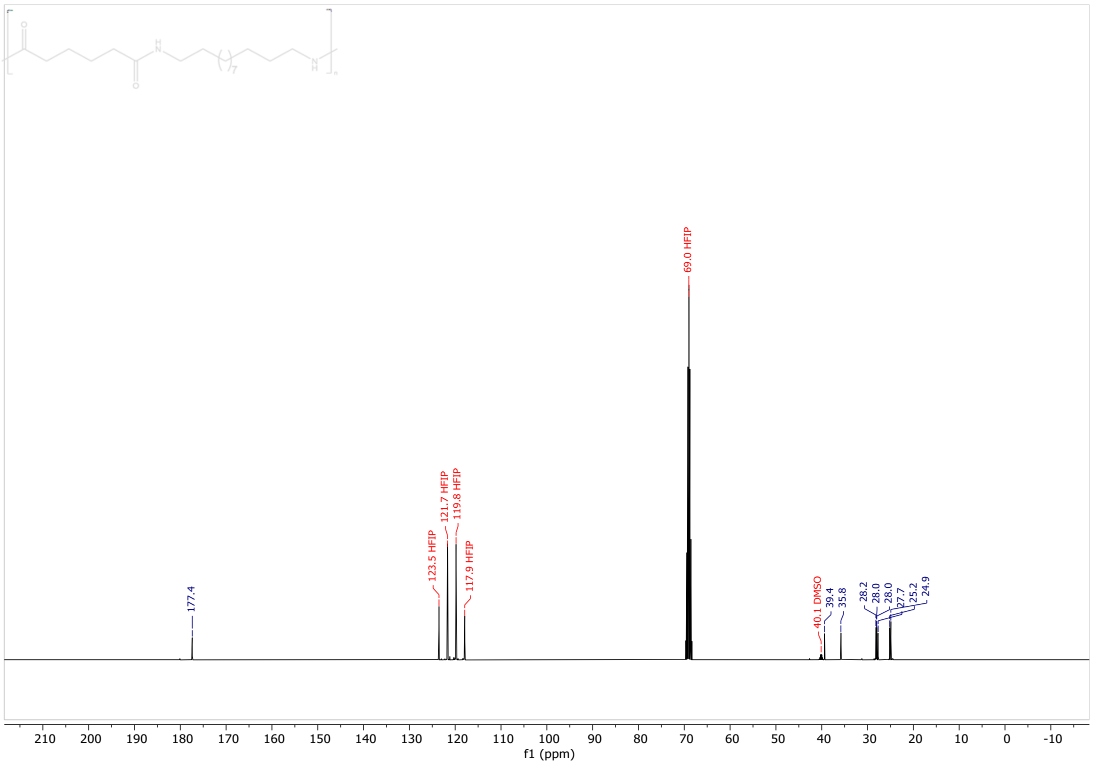

Figure S104 ^{13}C of nylon 6,12 in HFIP

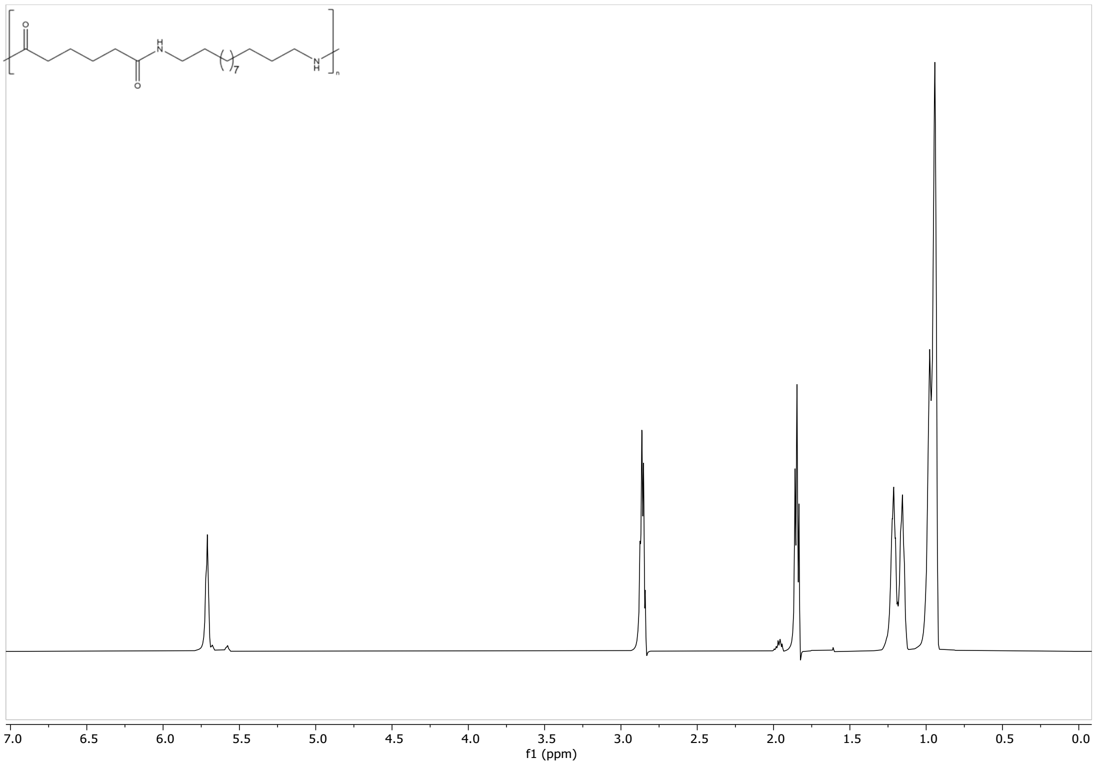

Figure S105 ^{1}H diffusion-edited spectra of nylon 6,12 in HFIP

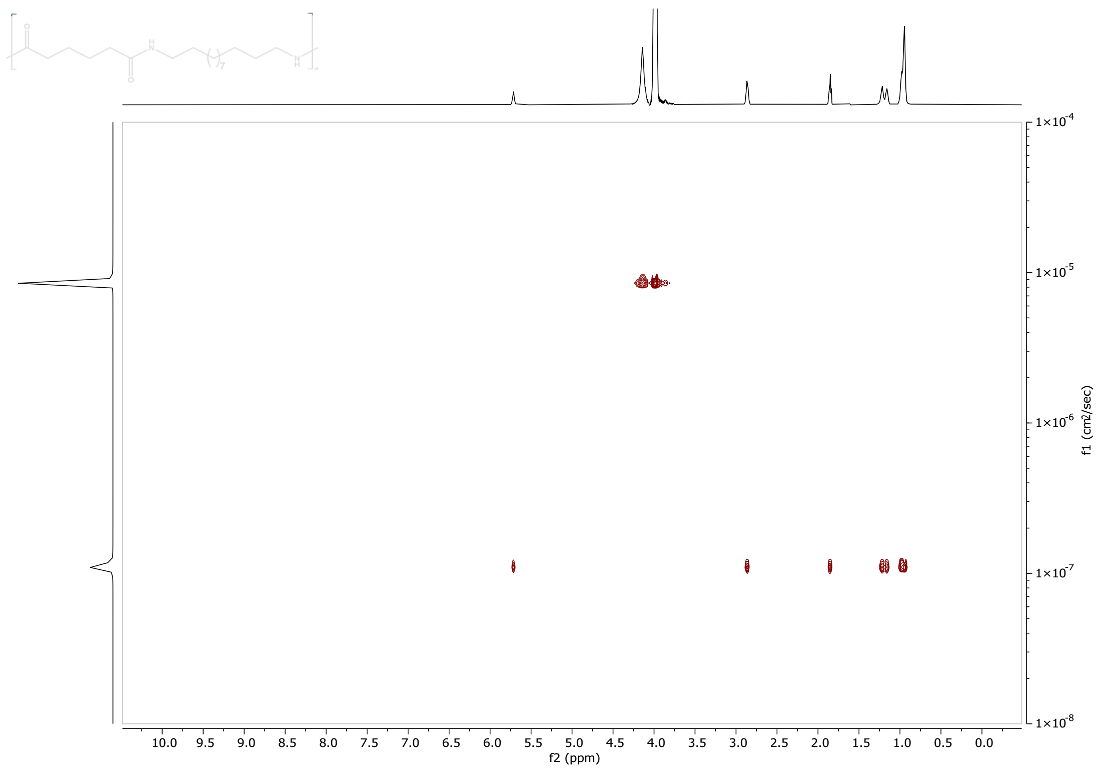

Figure S106 DOSY spectra of nylon 6,12

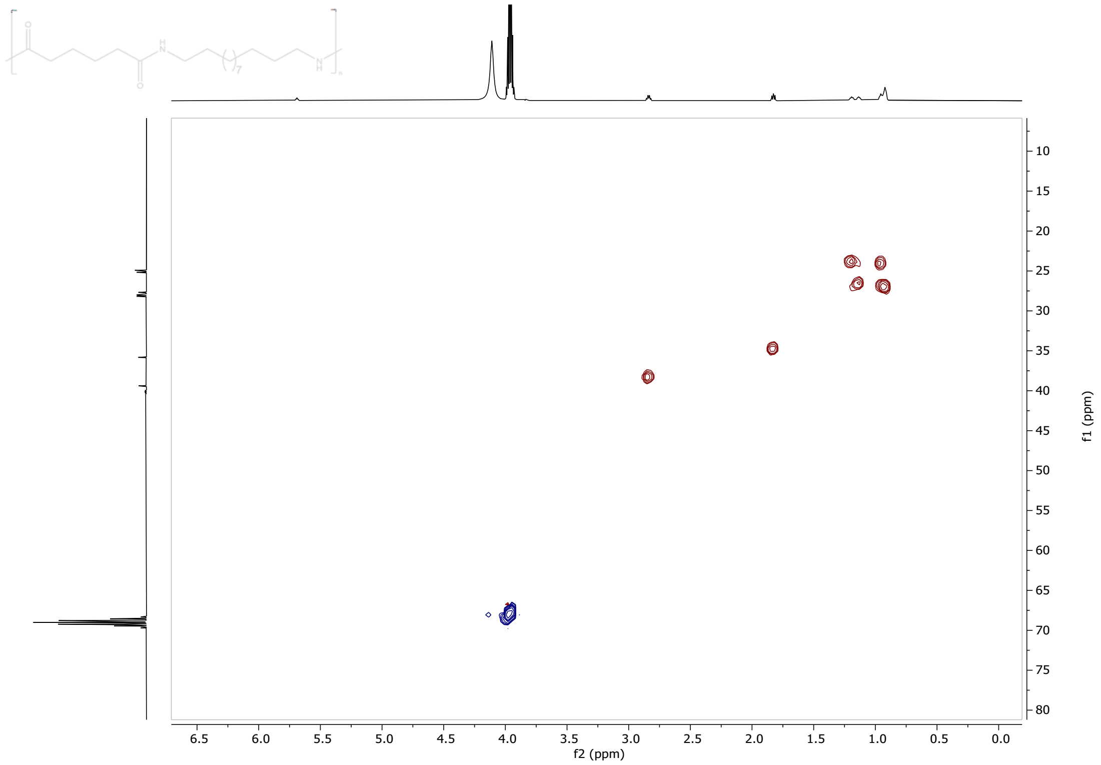

Figure S107 HSQC spectra of nylon 6,12

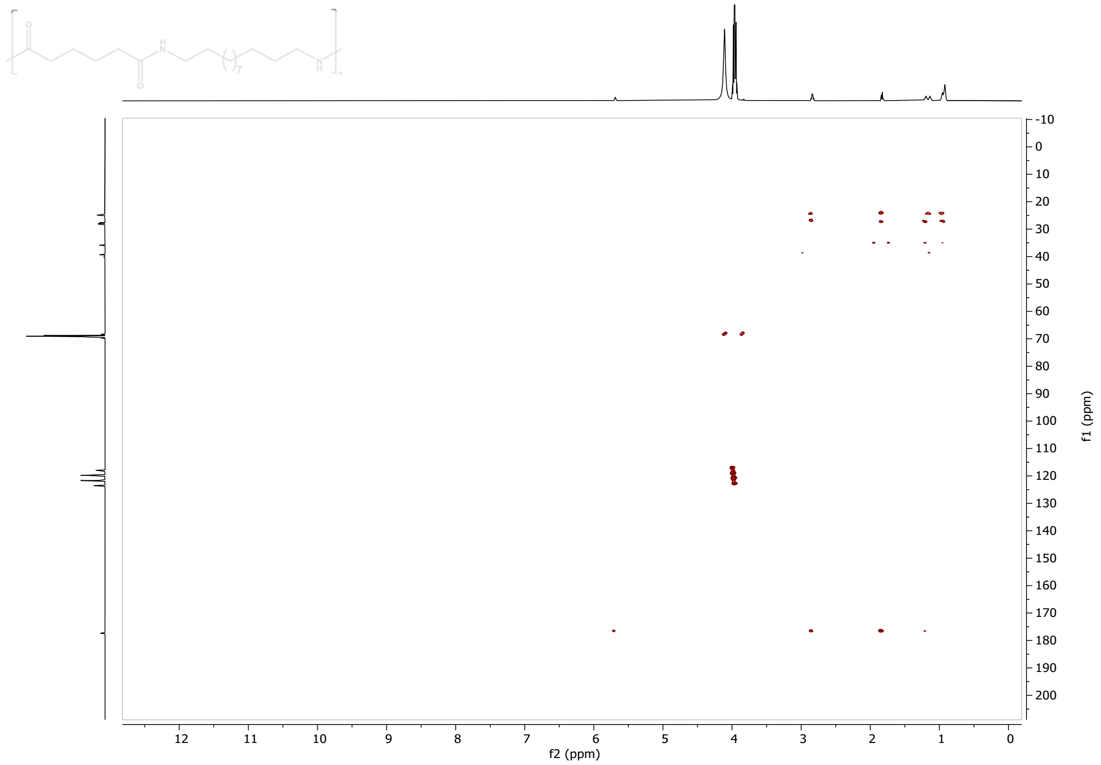

Figure S108 HMBC spectra of nylon 6,12

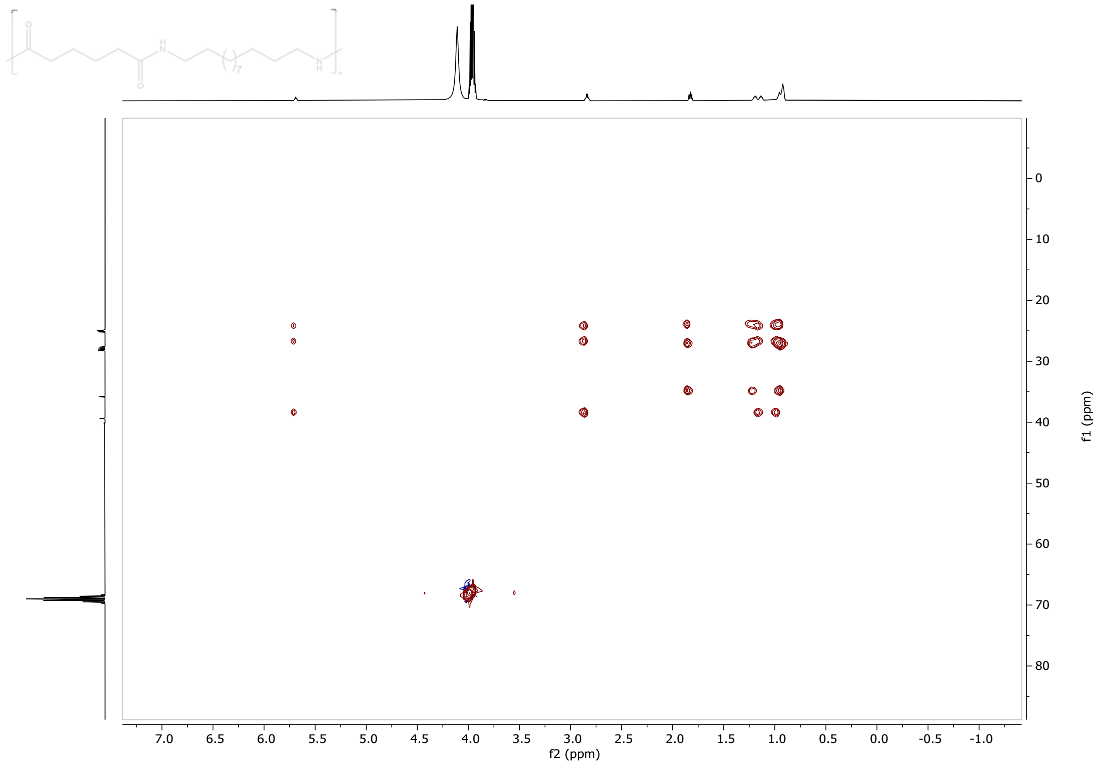

Figure S109 HSQC-TOCSY, (120 ms) of nylon 6,12 in HFIP

e. Characterisation of virgin nylon 11

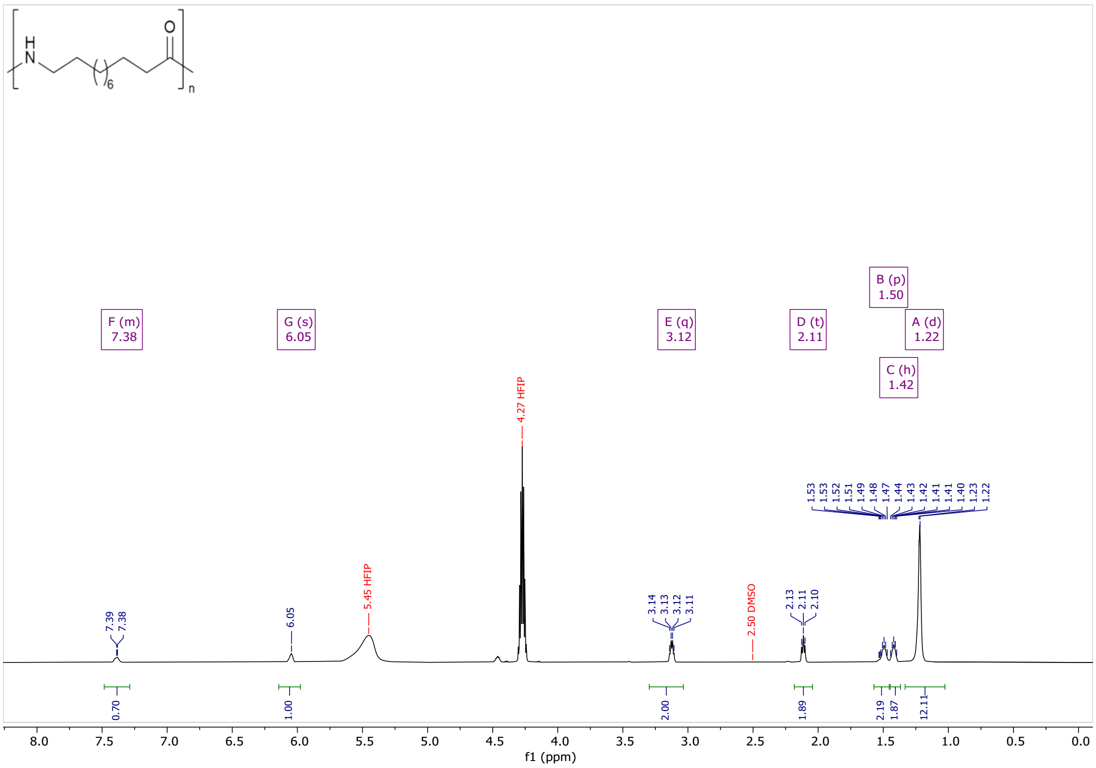

Figure S110 ^{1}H of nylon 11 in HFIP

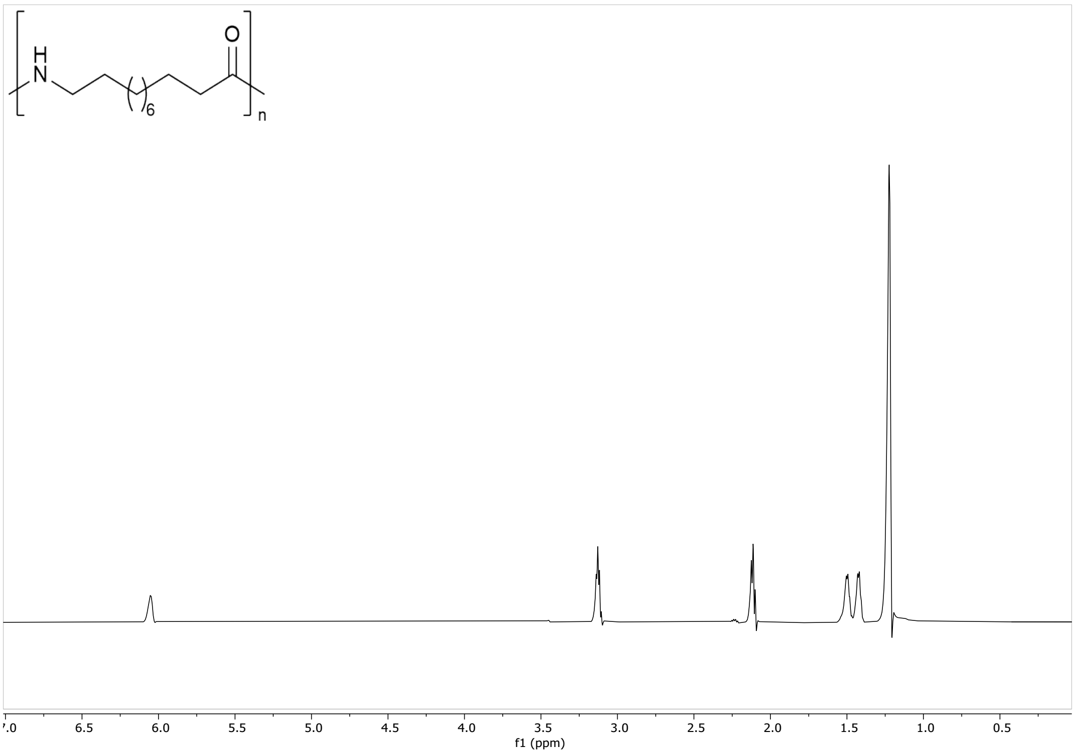

Figure S111 ^{1}H diffusion-edited of nylon 11 in HFIP

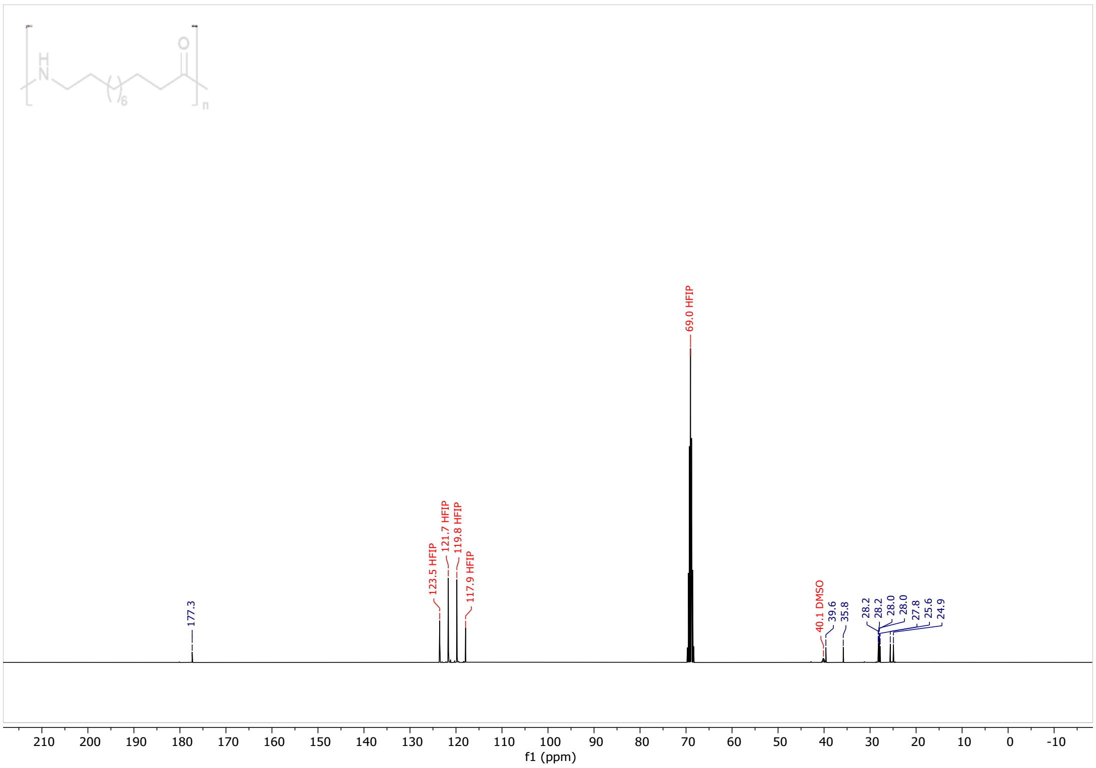

Figure S112 ^{13}C diffusion-edited of nylon 11 in HFIP

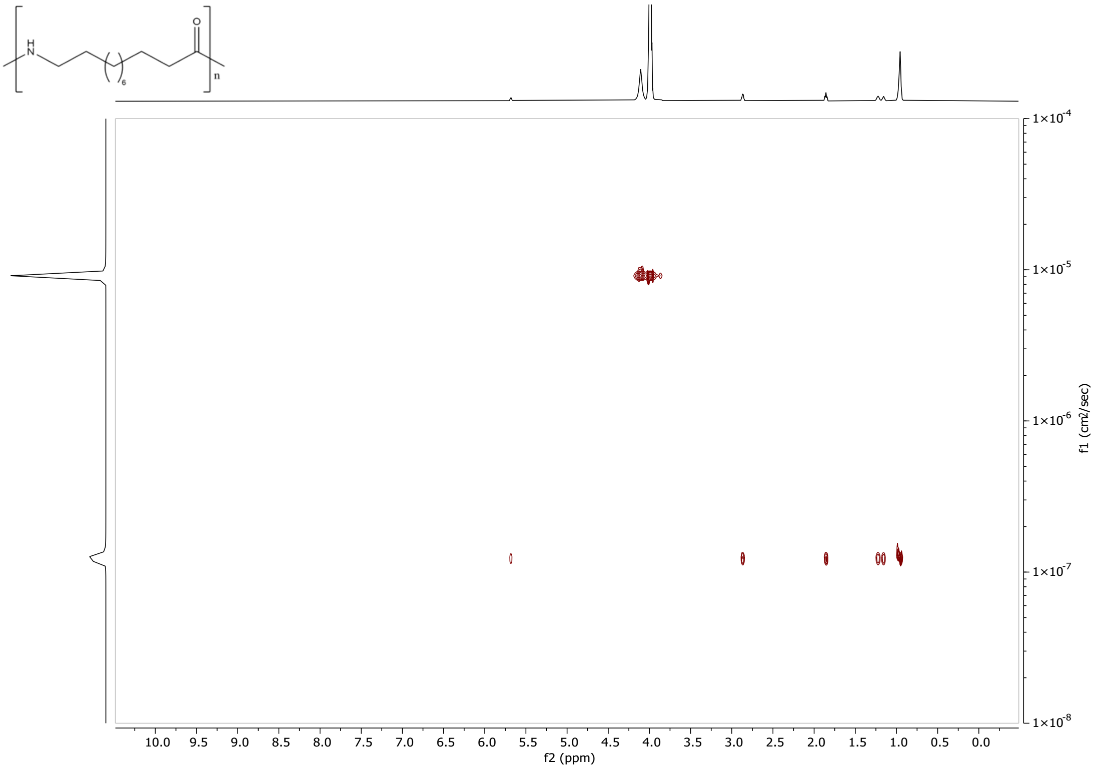

Figure S113 DOSY spectra of nylon 11

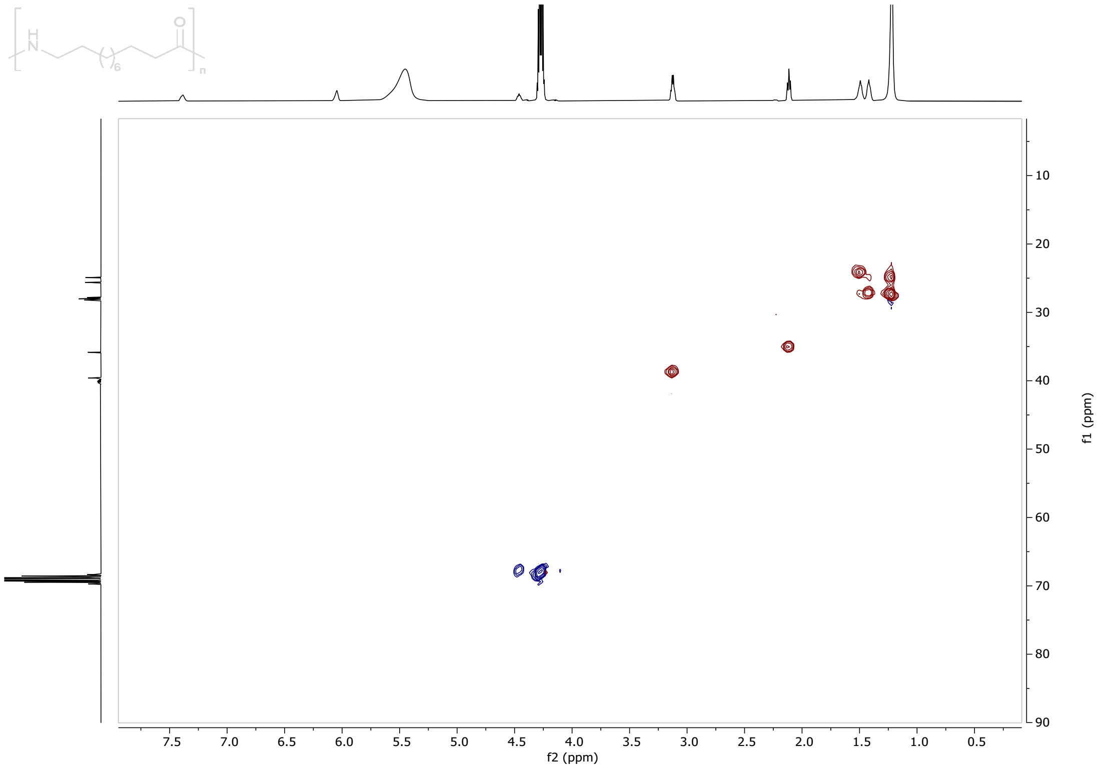

Figure S114 HSQC of nylon 11 in HFIP

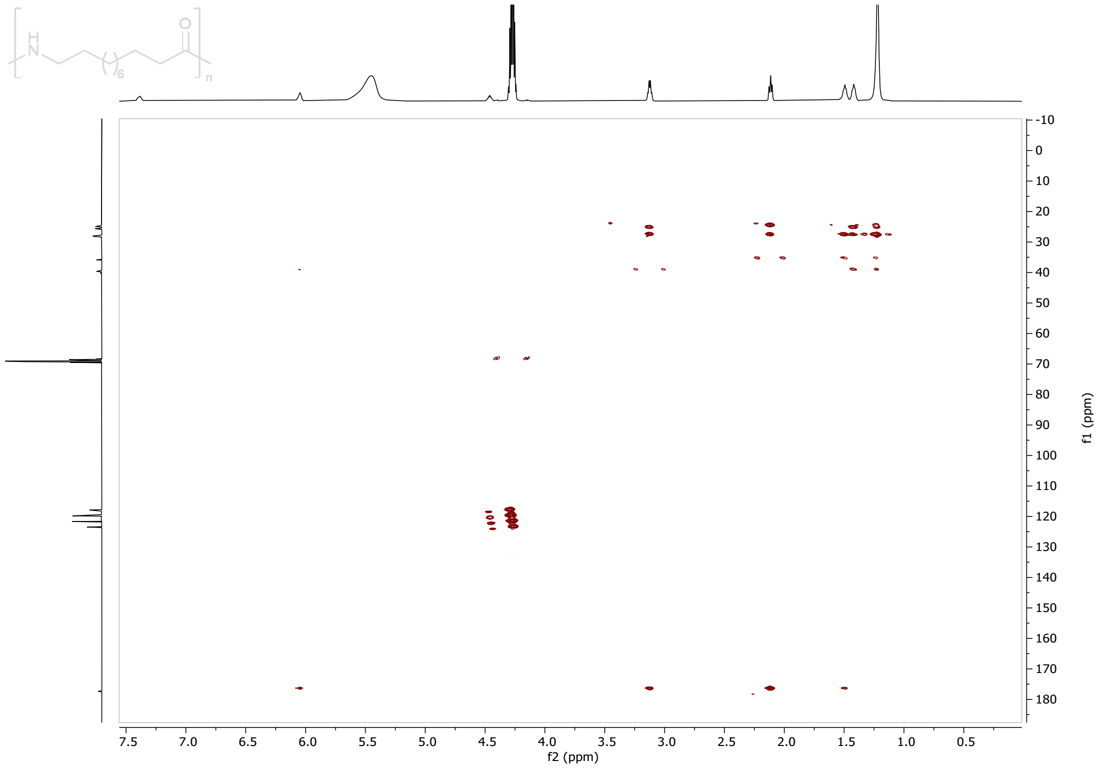

Figure S115 HMBC of nylon 11 in HFIP

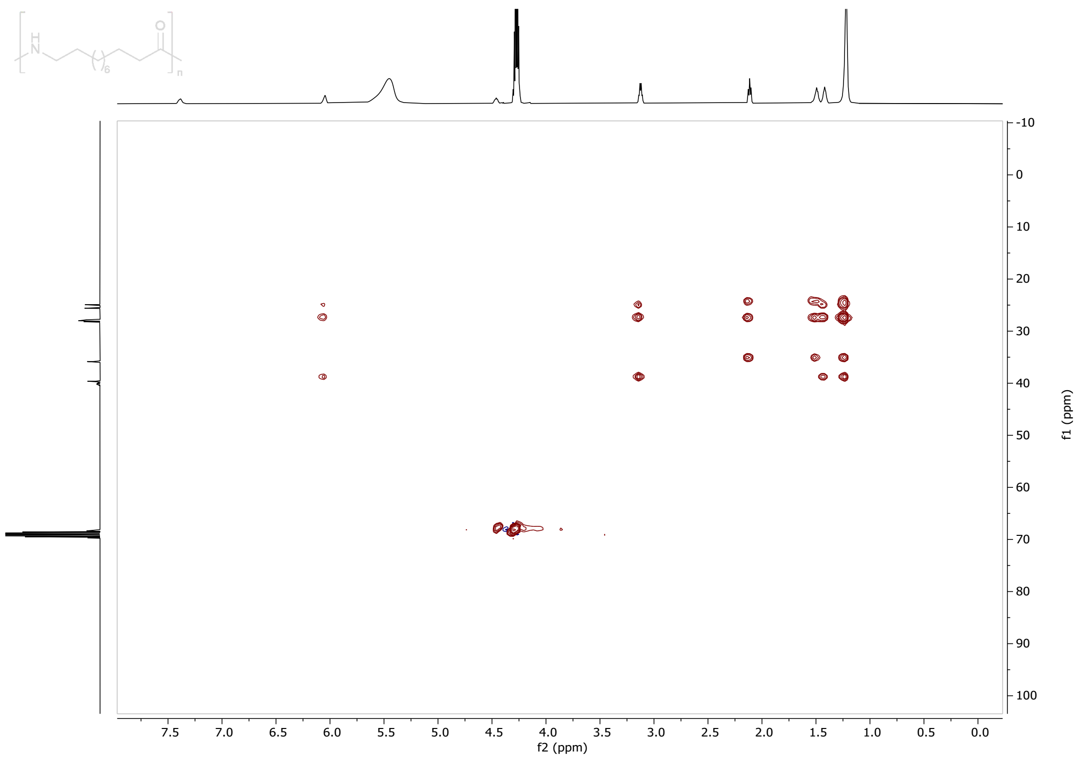

Figure S116 HSQC-TOCSY, (120 ms) of nylon 11 in HFIP

f. Characterisation of virgin nylon 12

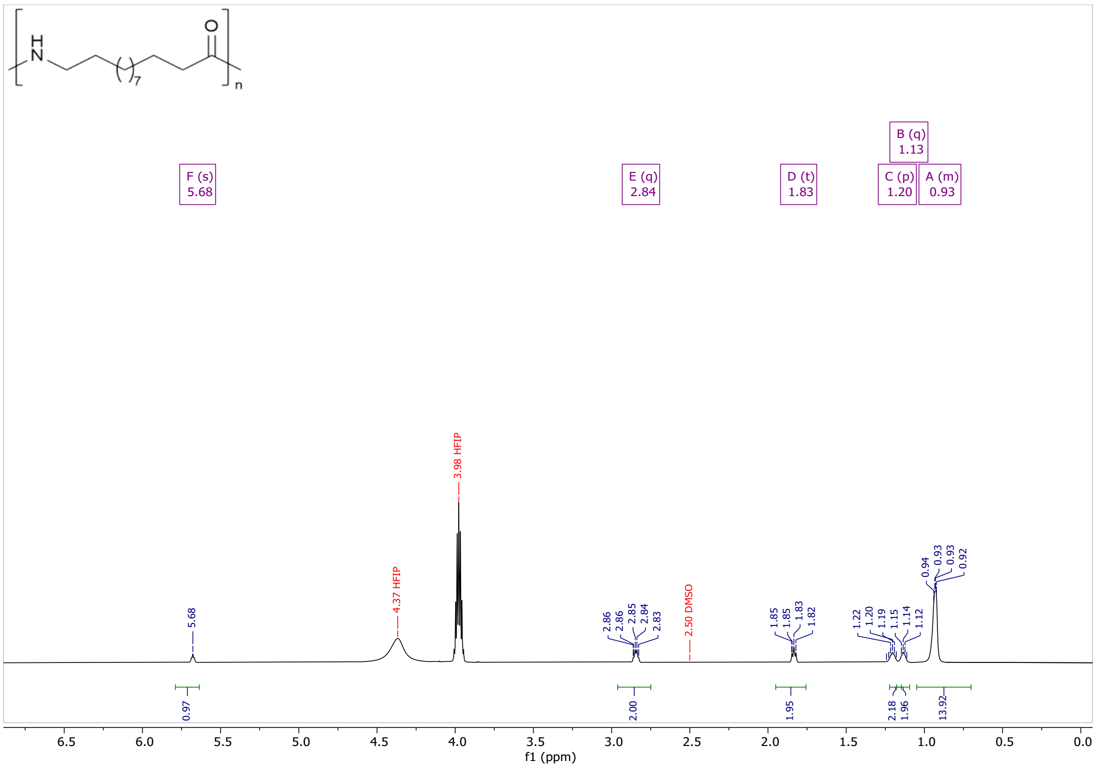

Figure S117 ^{1}H of nylon 12 in HFIP

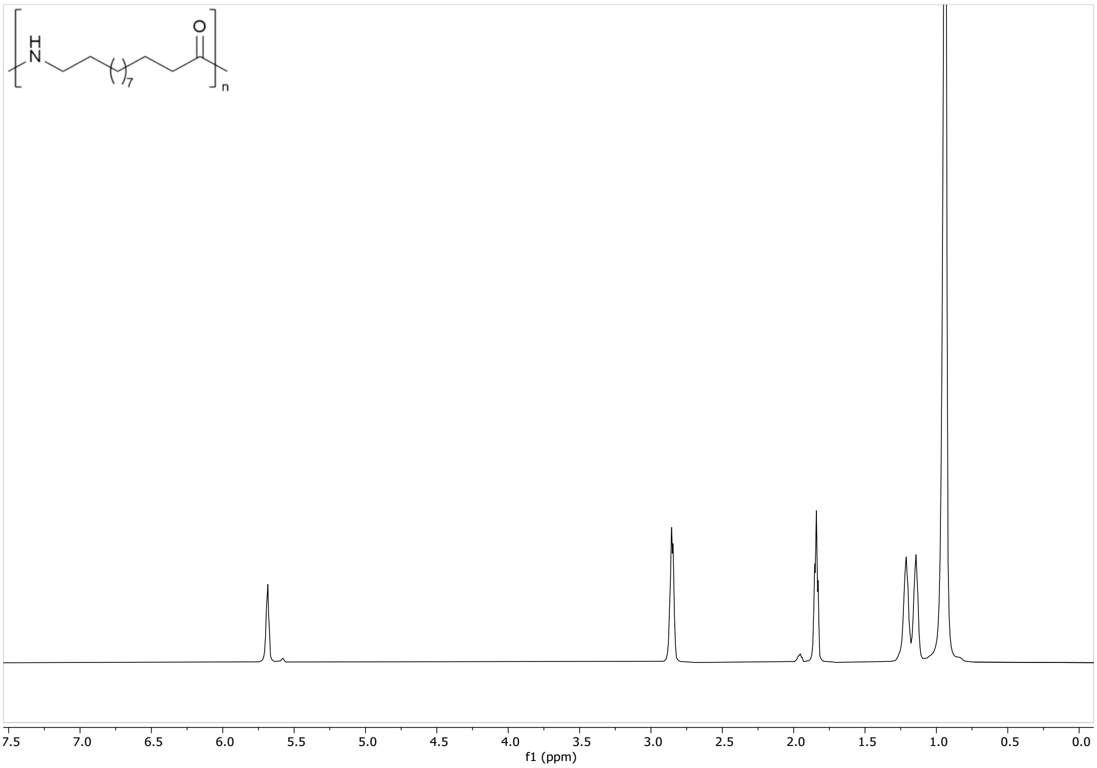

Figure S118 ^{1}H diffusion-edited of nylon 12 in HFIP

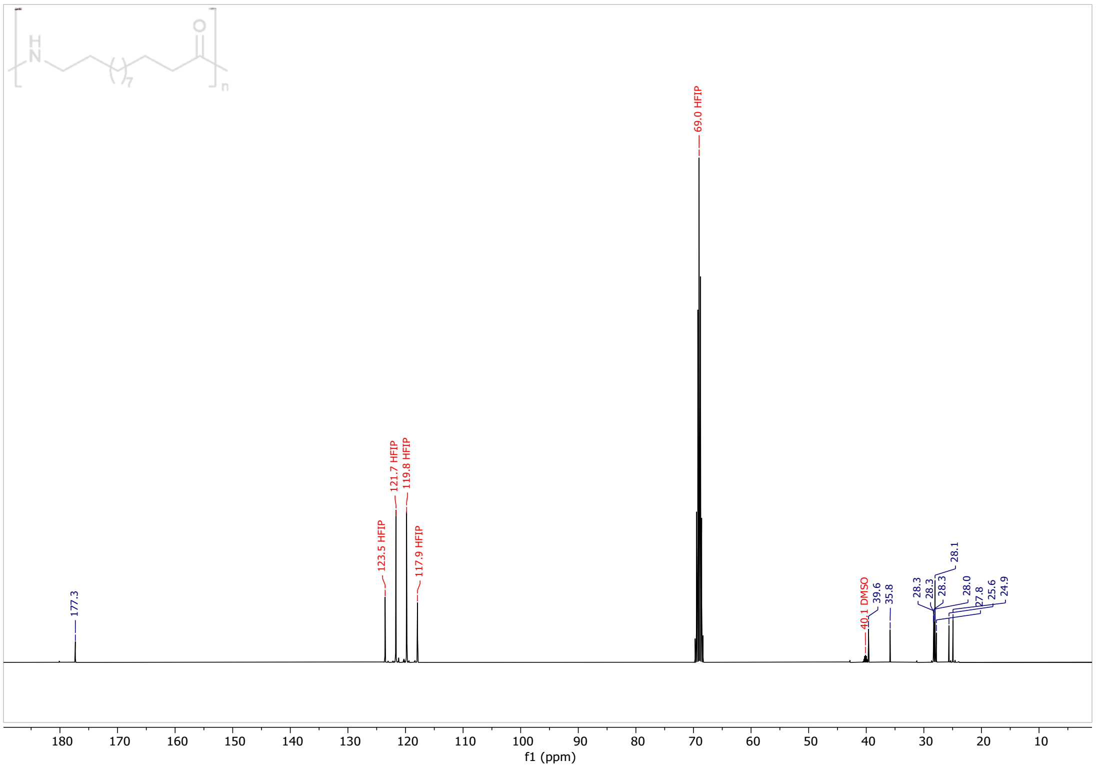

Figure S119 ^{13}C diffusion-edited of nylon 12 in HFIP

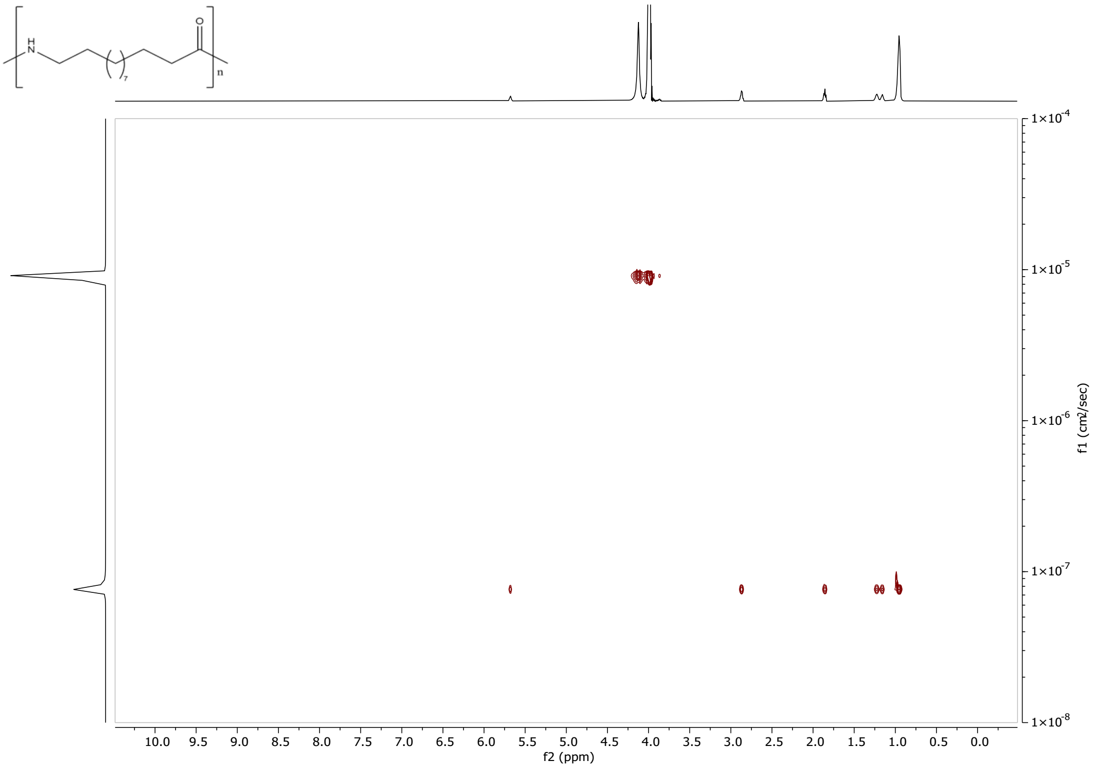

Figure S120 DOSY spectra of nylon 12

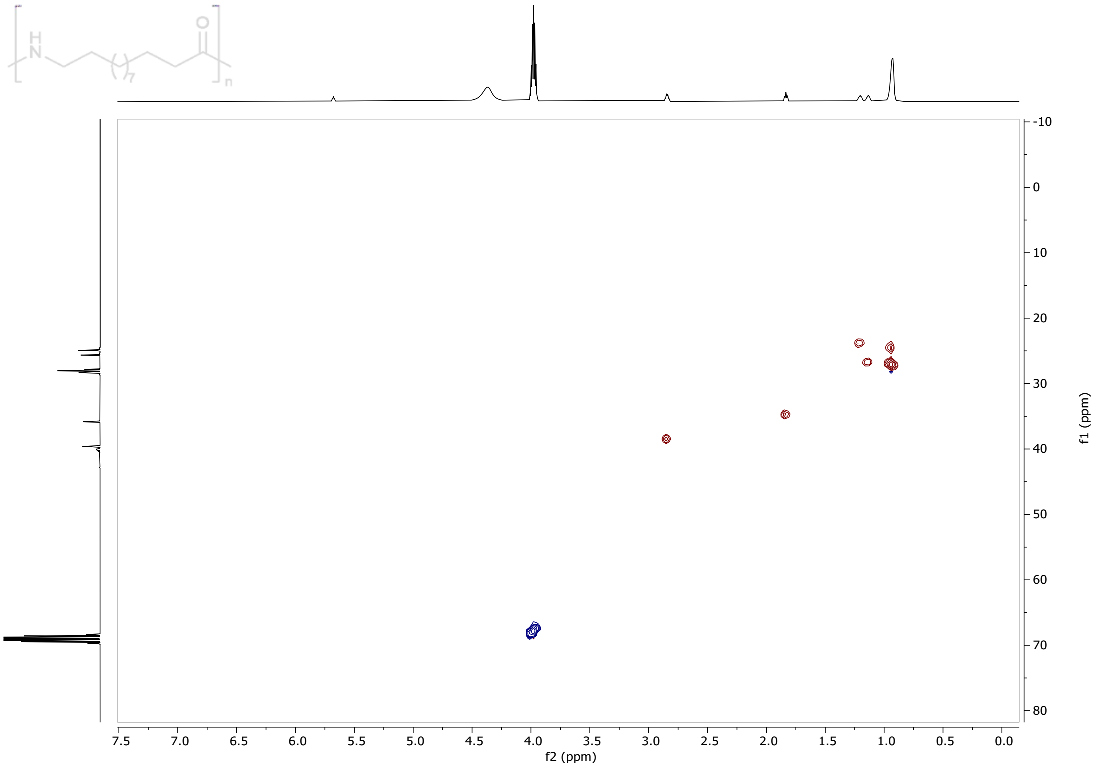

Figure S121 HSQC of nylon 12 in HFIP

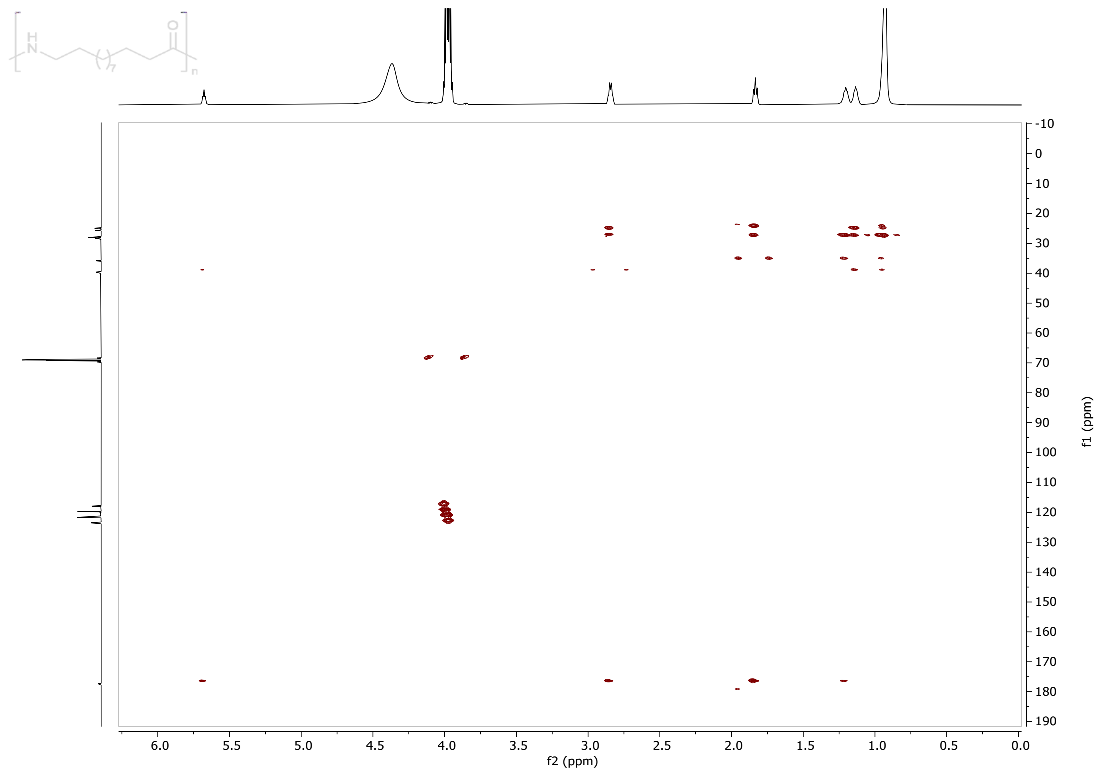

Figure S122 HMBC of nylon 12 in HFIP

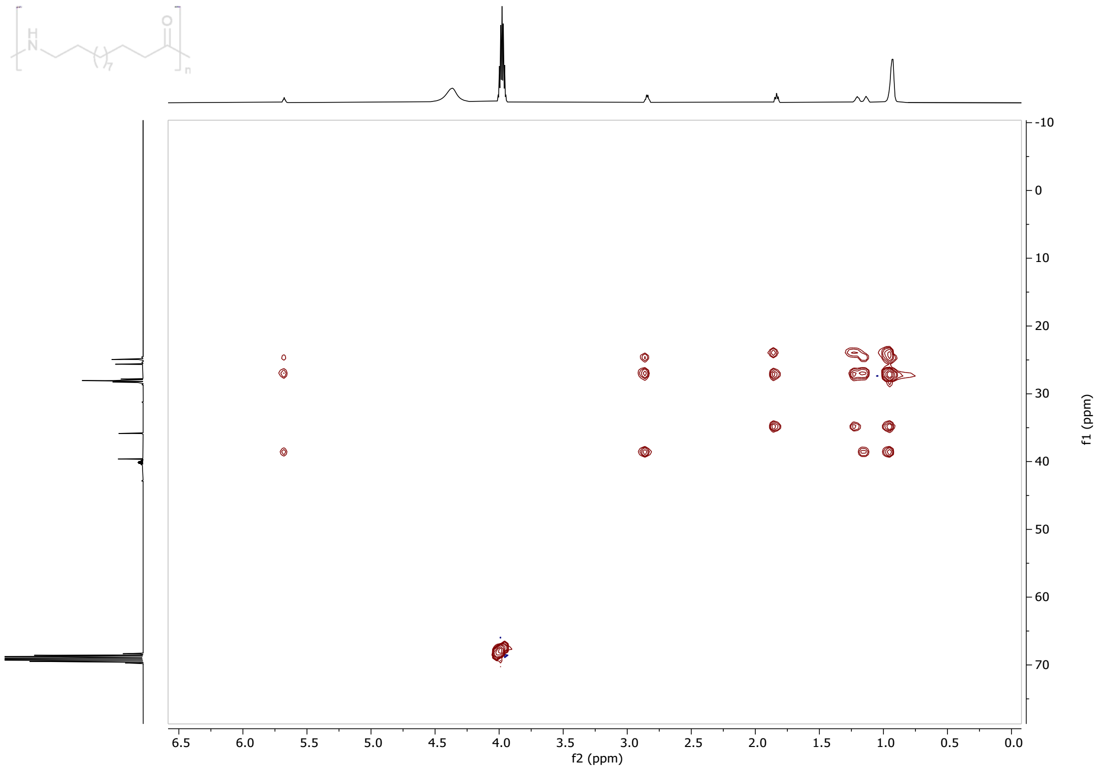

Figure S123 HSQC-TOCSY, (120 ms) of nylon 11 in HFIP

g. Characterisation of nylon 6 dissolved in [mTBDH][OAc]

NMR reports:

Nylon 6 in [mTBDH][OAc]: **^{1}H NMR** (600 MHz, DMSO) δ 2.26 (s, 2H), 2.03 (d, J = 7.6 Hz, 2H), 1.44 (t, J = 7.7 Hz, 2H), 1.36 (s, 2H), 1.22 – 1.17 (m, 2H).

[mTBDH][OAc] with nylon 6 gave a dope liquid enough to provide a good quality characterization.

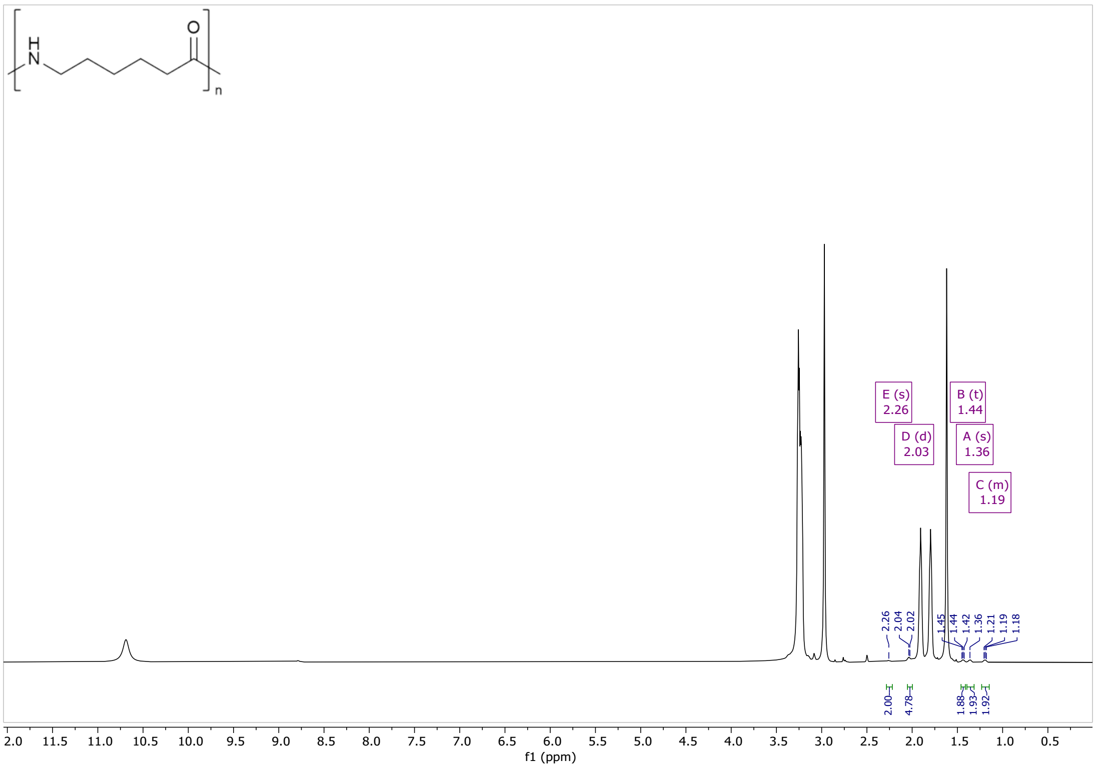

Figure S124 ^{1}H of nylon 6 dissolved in [mTBDH][OAc] at 65°C with a sealed capillary containing DMSO-d_6

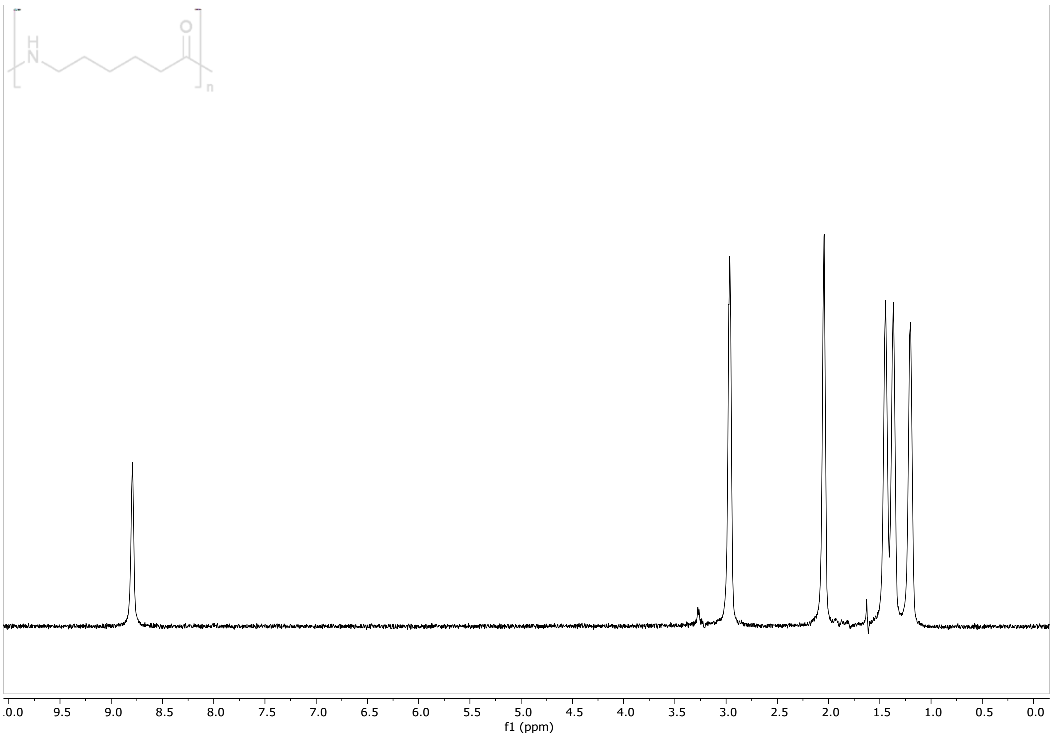

Figure S125 ^{1}H diffusion-edited of nylon 6 dissolved in [mTBDH][OAc] at 65°C with a sealed capillary containing DMSO-d_6

h. Characterisation of nylon 6 dissolved in [dm$_3$-mTBDH][OAc]

[dm$_3$-mTBDH][OAc] with nylon 6 gave a dope liquid enough to provide a good quality characterization.

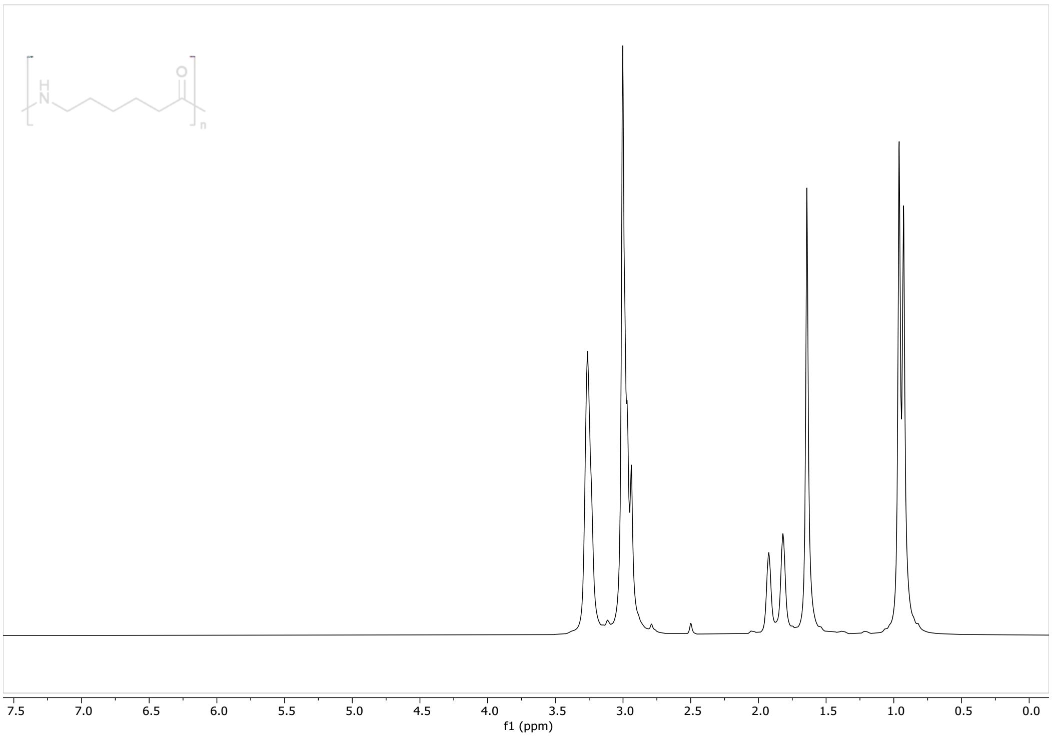

Figure S126 ^{1}H of nylon 6 dissolved in [dm$_3$-mTBDH][OAc] at 65°C with a sealed capillary containing DMSO-d$_6$

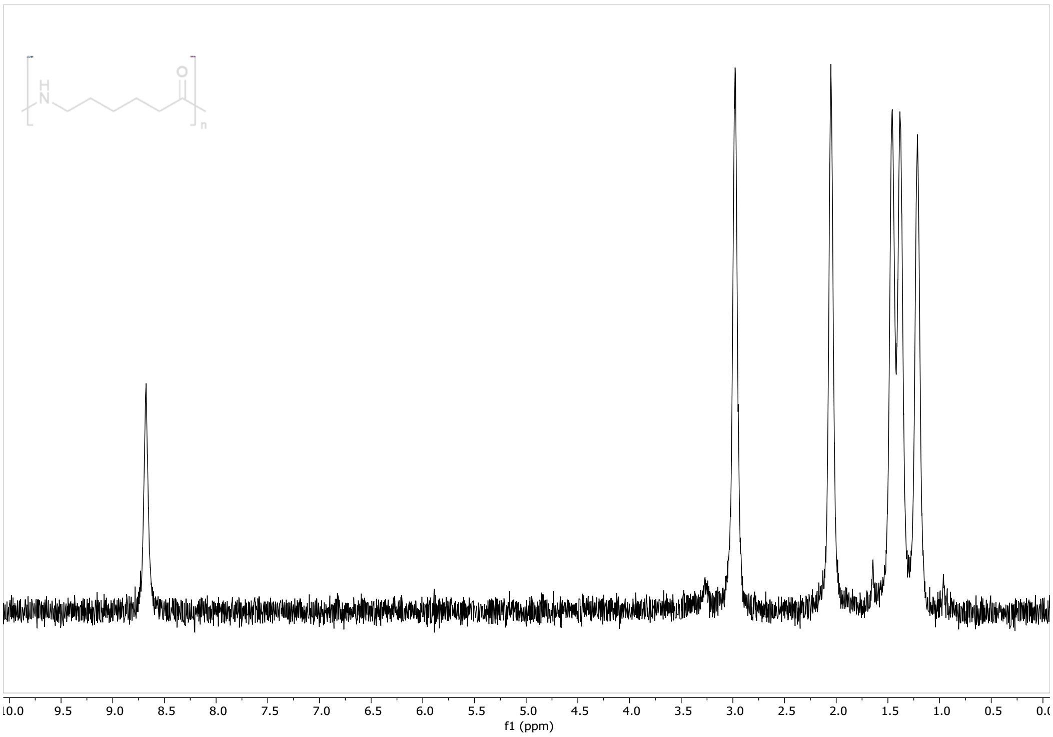

Figure S127 ^{1}H diffusion edited of nylon 6 dissolved in [mTBDH][OAc] at 65°C with a sealed capillary containing DMSO-d$_6$

i. <u>Characterisation of nylon 6 in TFE</u>

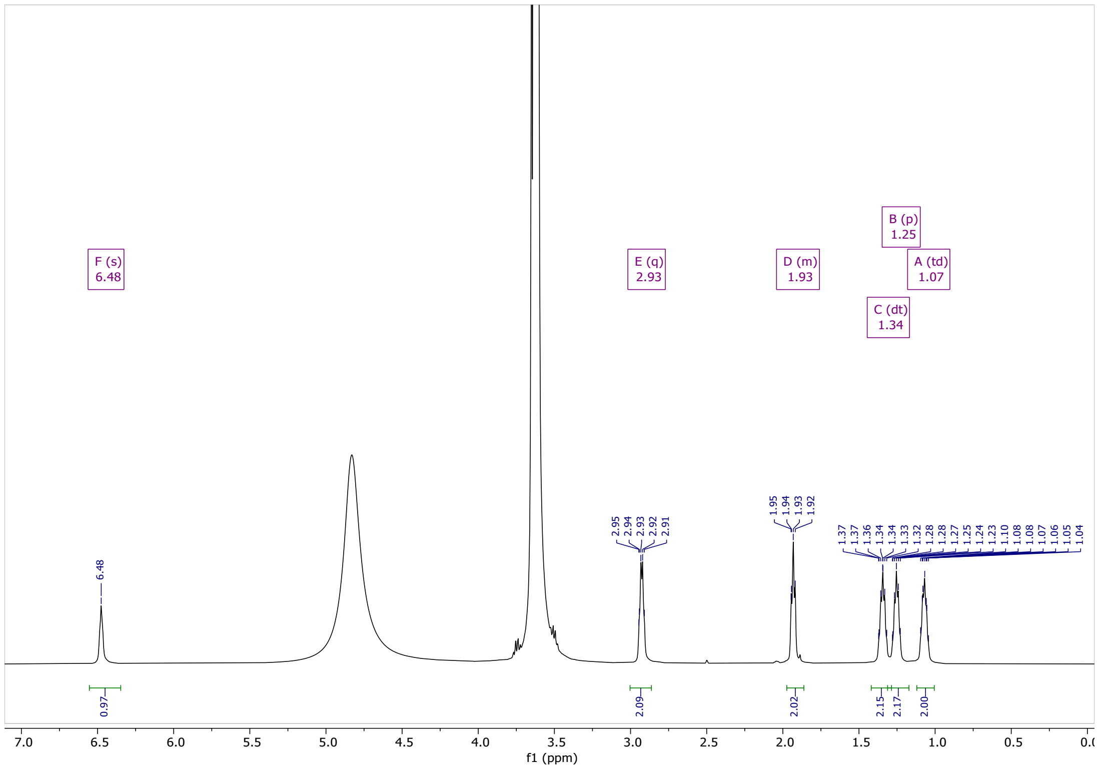

Figure S128 ^{1}H of nylon 6 dissolved in TFE

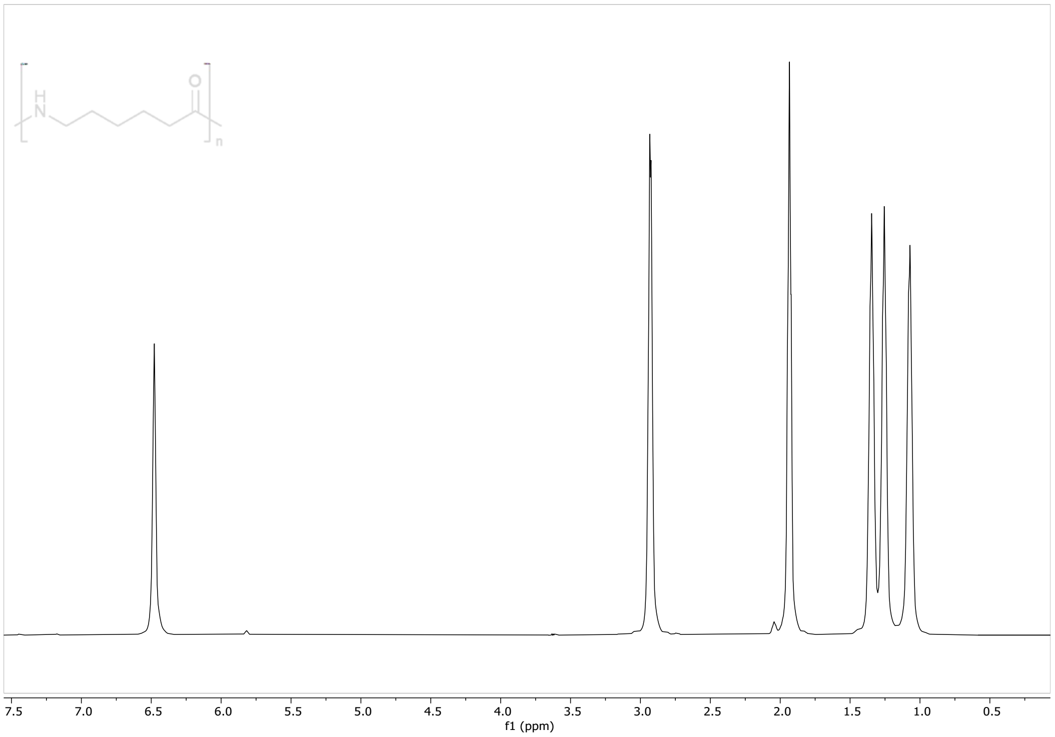

Figure S129 ^{1}H diffusion edited of nylon 6 dissolved in TFE

j. Characterisation of nylon 6,6 (P) in TFE

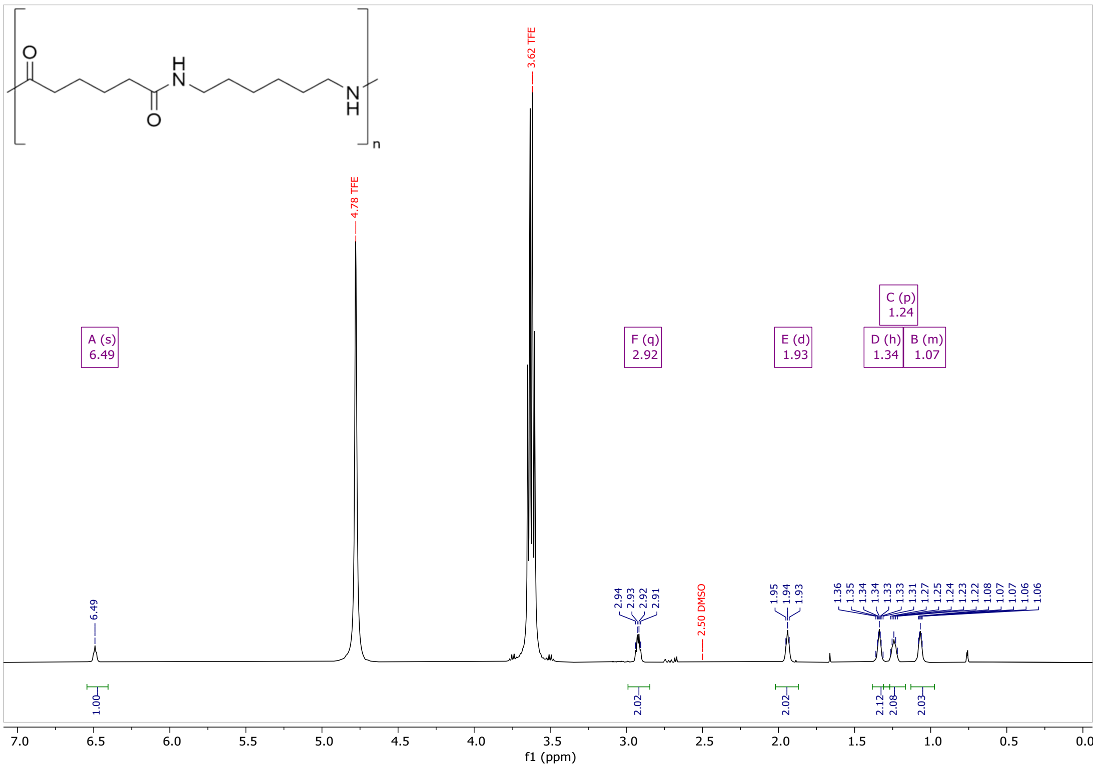

Figure S130 ¹H of nylon 6,6 (P) dissolved in TFE

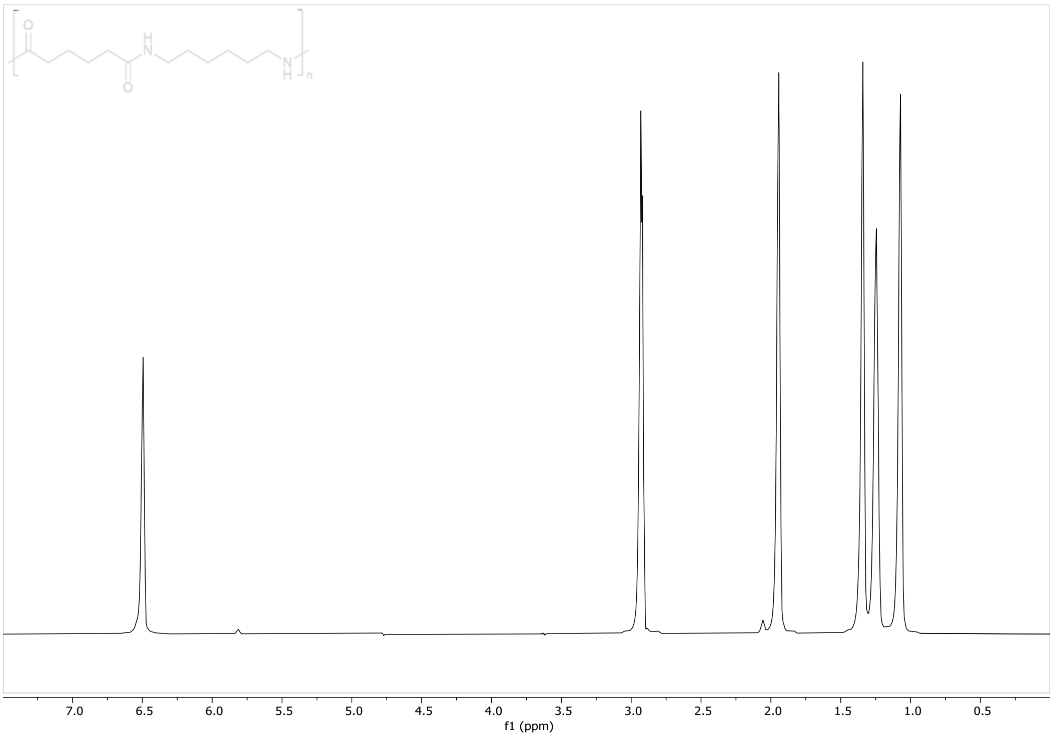

Figure S131 ¹H diffusion edited of nylon 6,6 (P) dissolved in TFE

k. Characterisation of nylon 6,6 (M) in TFE

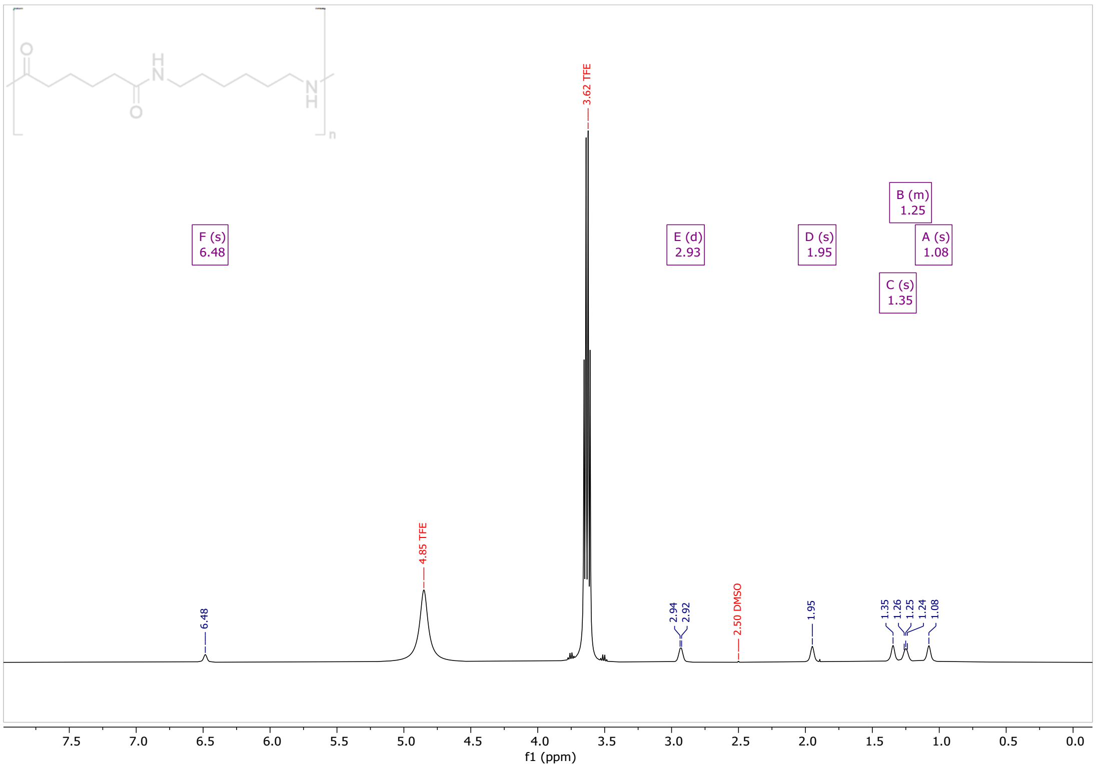

Figure S132 ¹H of nylon 6,6 (M) dissolved in TFE

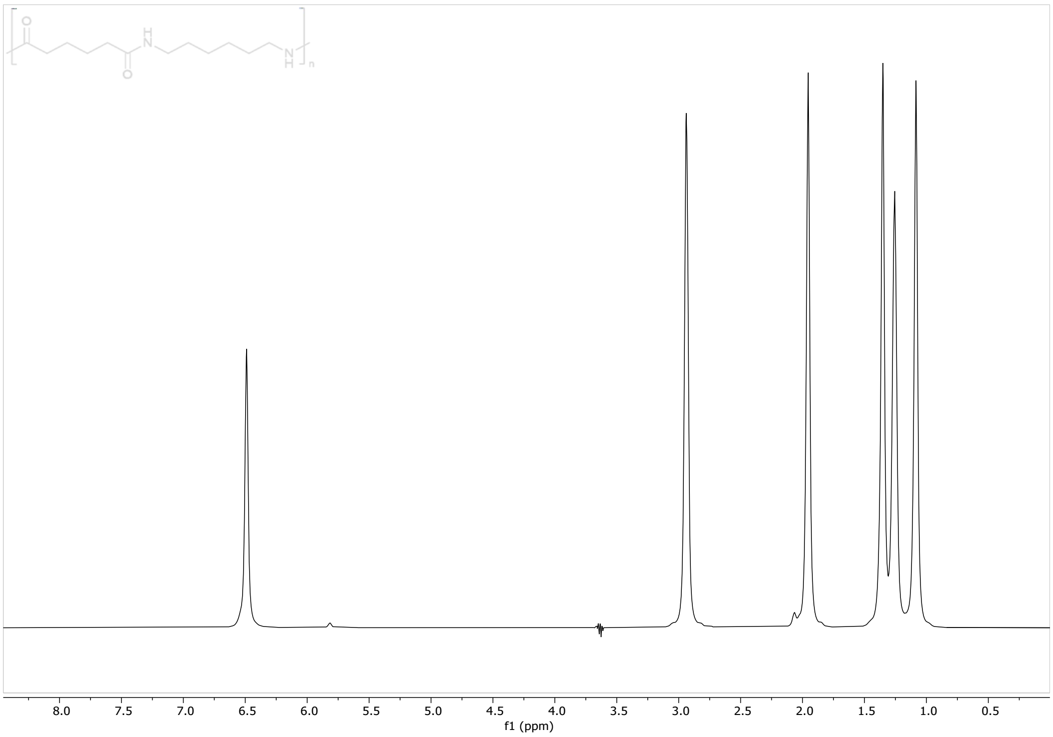

Figure S133 ¹H diffusion edited of nylon 6,6 (M) dissolved in TFE

I. Characterisation (^{1}H) of recovered nylons from [dm$_3$-mTBDH][OAc]

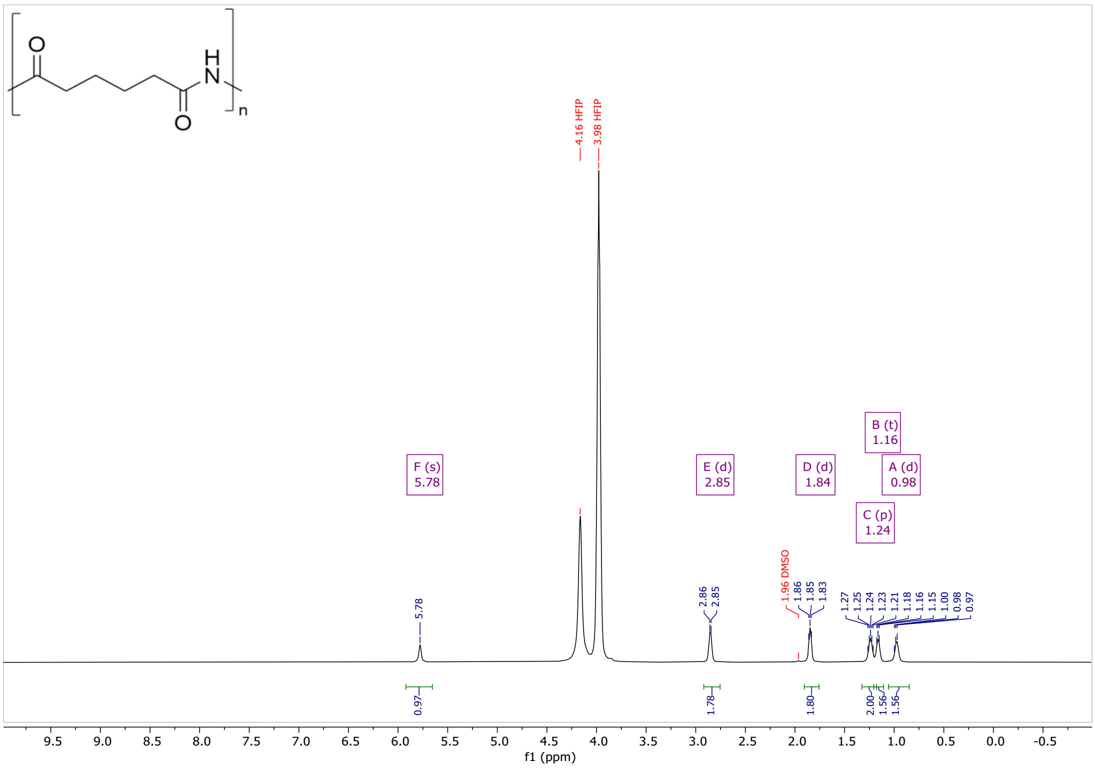

Figure S134 ^{1}H NMR of nylon 6 after dissolution and regeneration from [dm$_3$-mTBDH][OAc]

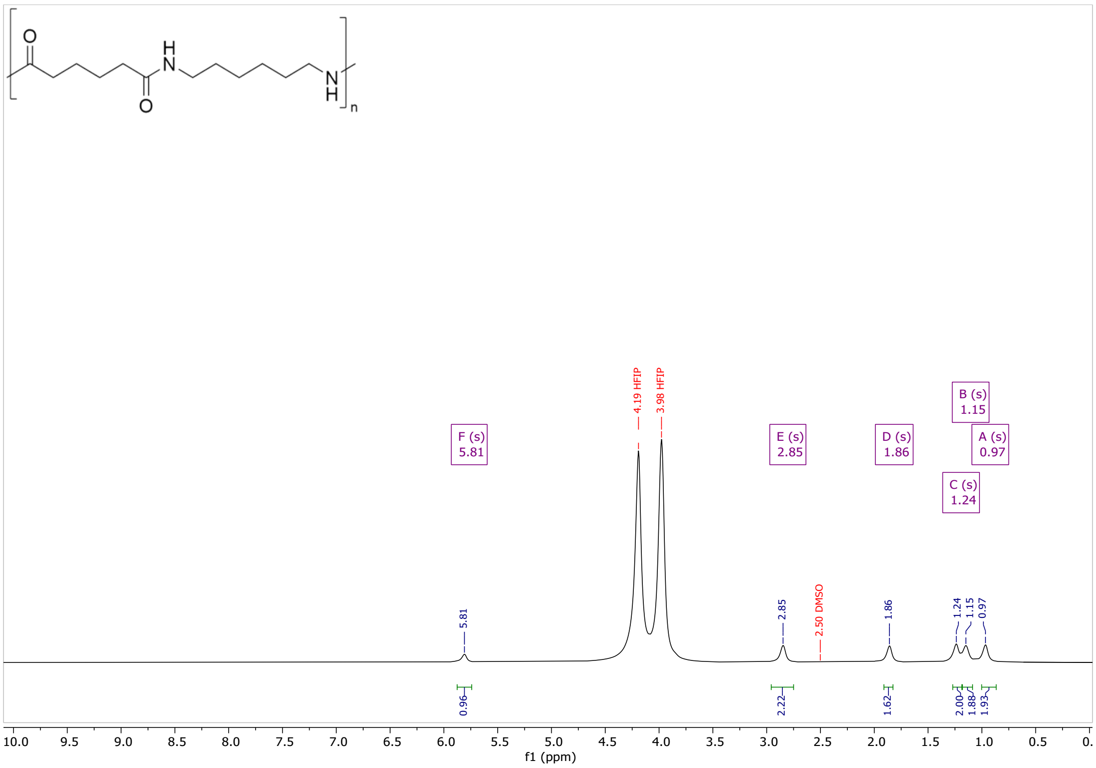

Figure S135 ^{1}H NMR of nylon 6,6 (P) after dissolution and regeneration from [dm$_3$-mTBDH][OAc]

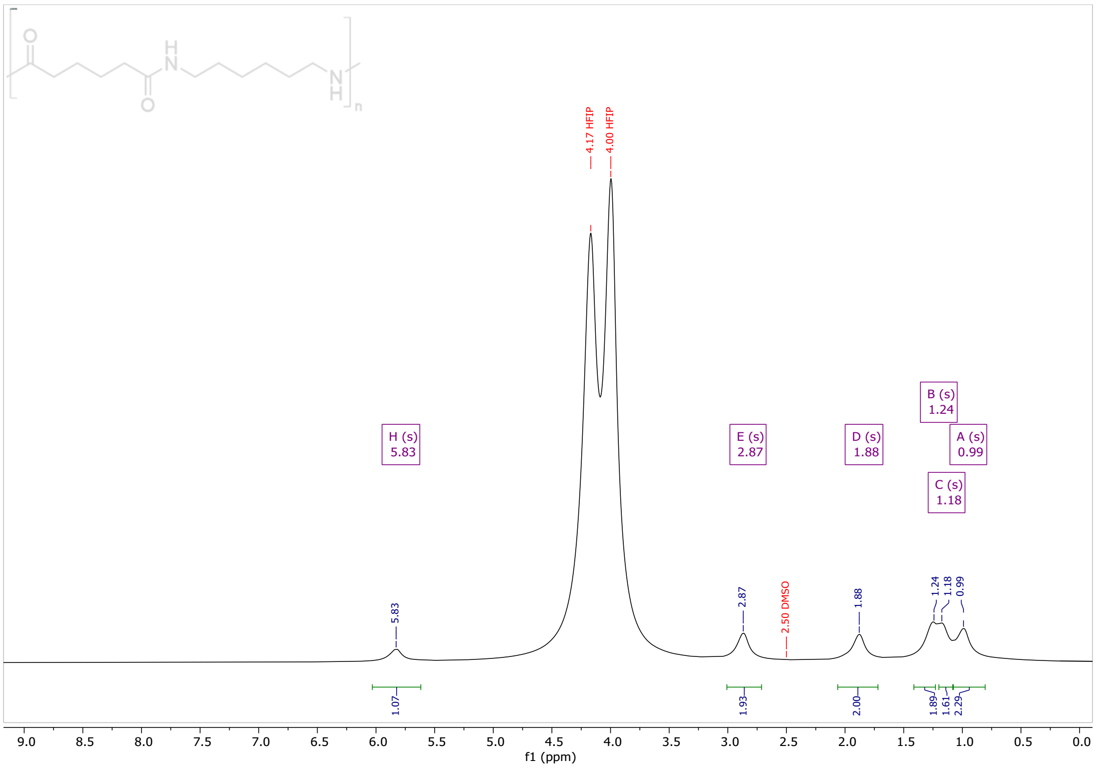

Figure S136 ^{1}H NMR of nylon 6,6 (M) after dissolution and regeneration from [dm$_3$-mTBDH][OAc]

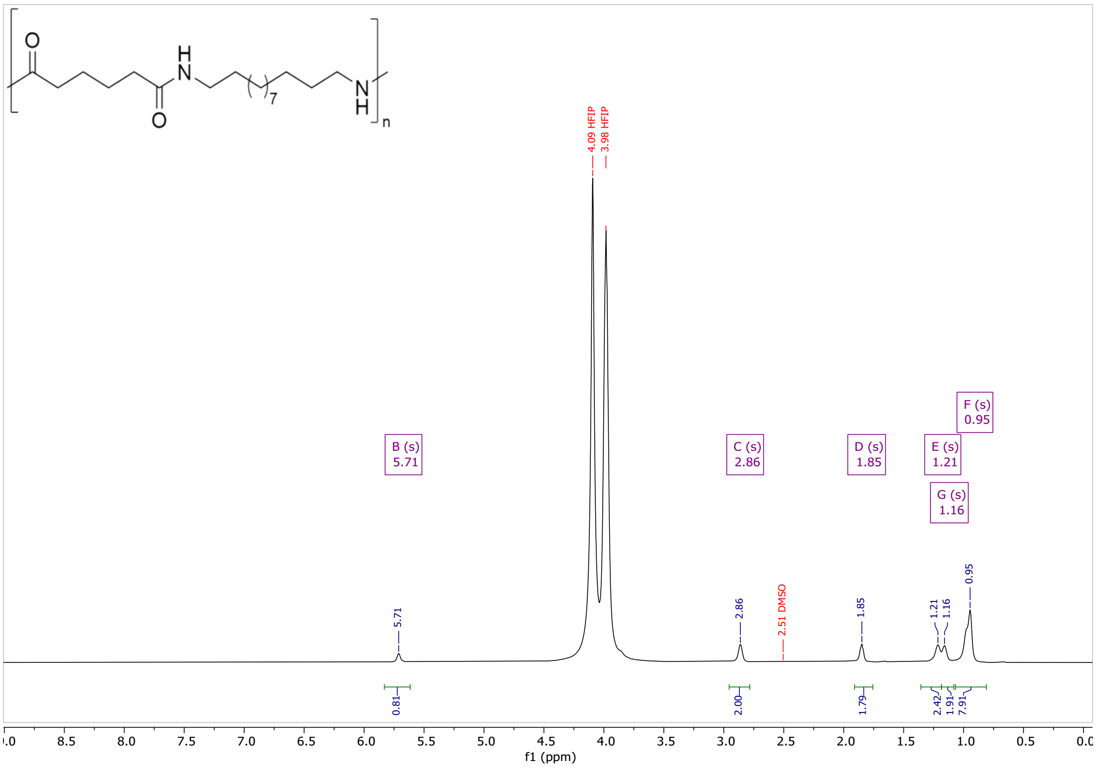

Figure S137 ^{1}H NMR of nylon 6,12 after dissolution and regeneration from [dm$_3$-mTBDH][OAc]

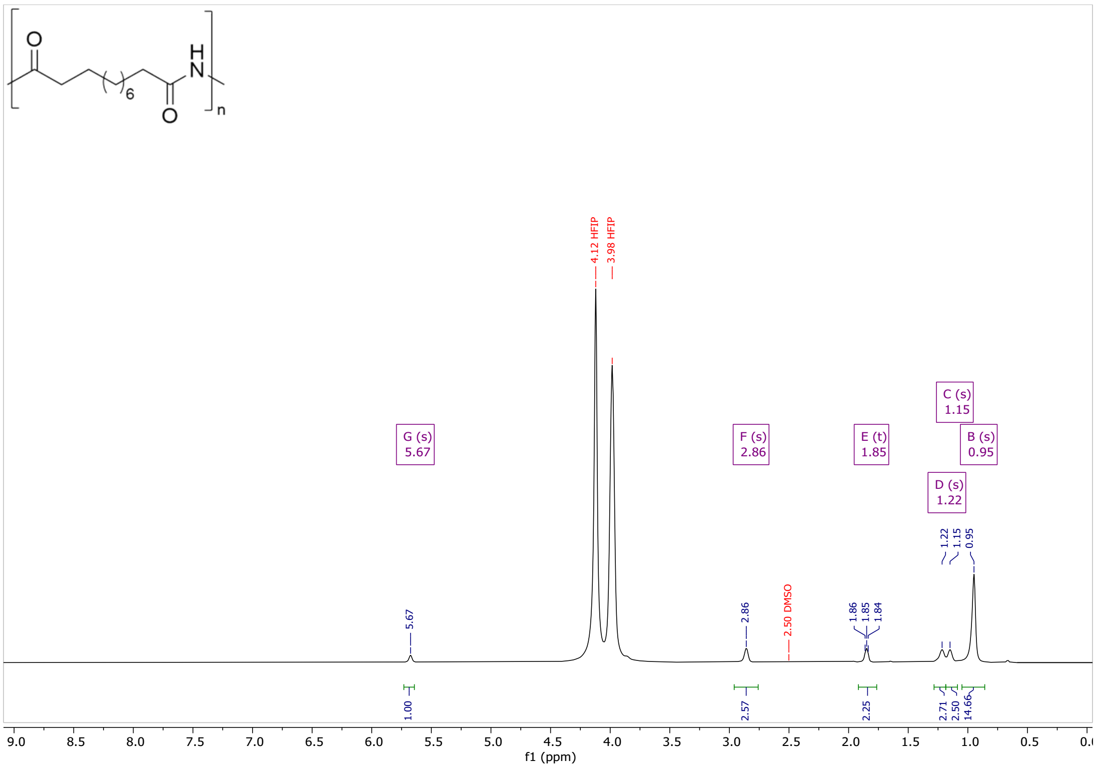

Figure S138 ^{1}H NMR of nylon 11 after dissolution and regeneration from [dm$_3$-mTBDH][OAc]

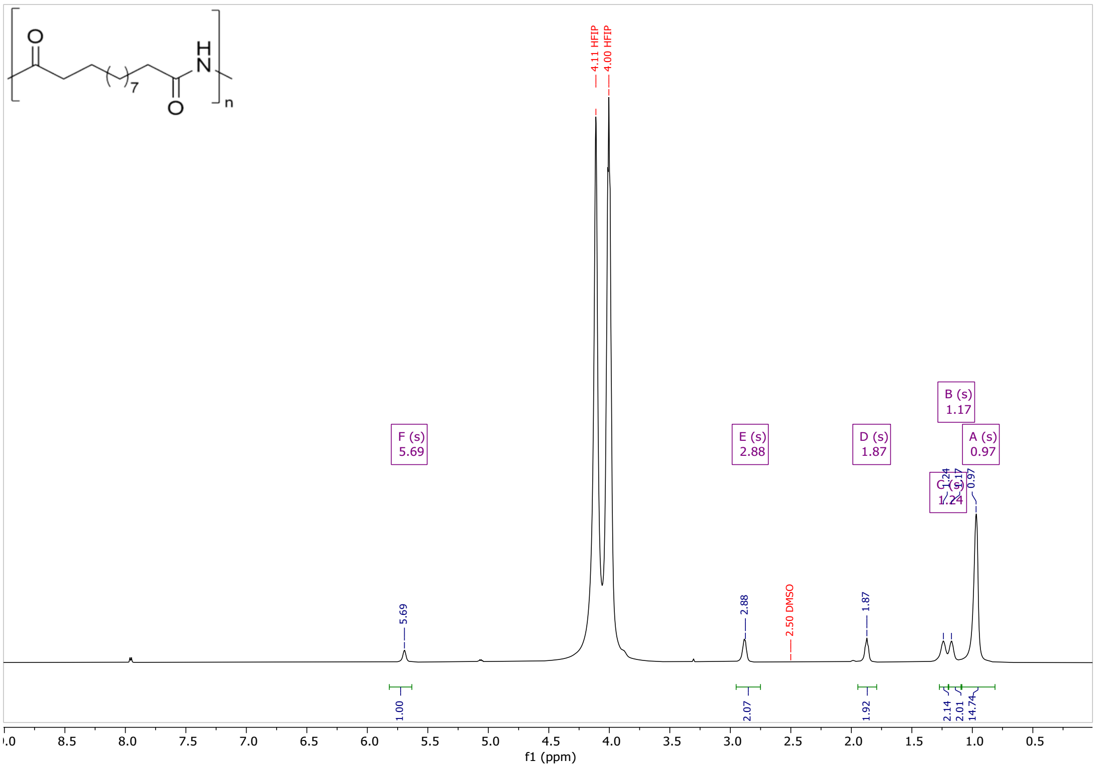

Figure S139 ^{1}H NMR of nylon 12 after dissolution and regeneration from [dm3-mTBDH][OAc]

m. Characterisation (^{1}H) of recovered nylons from HFIP

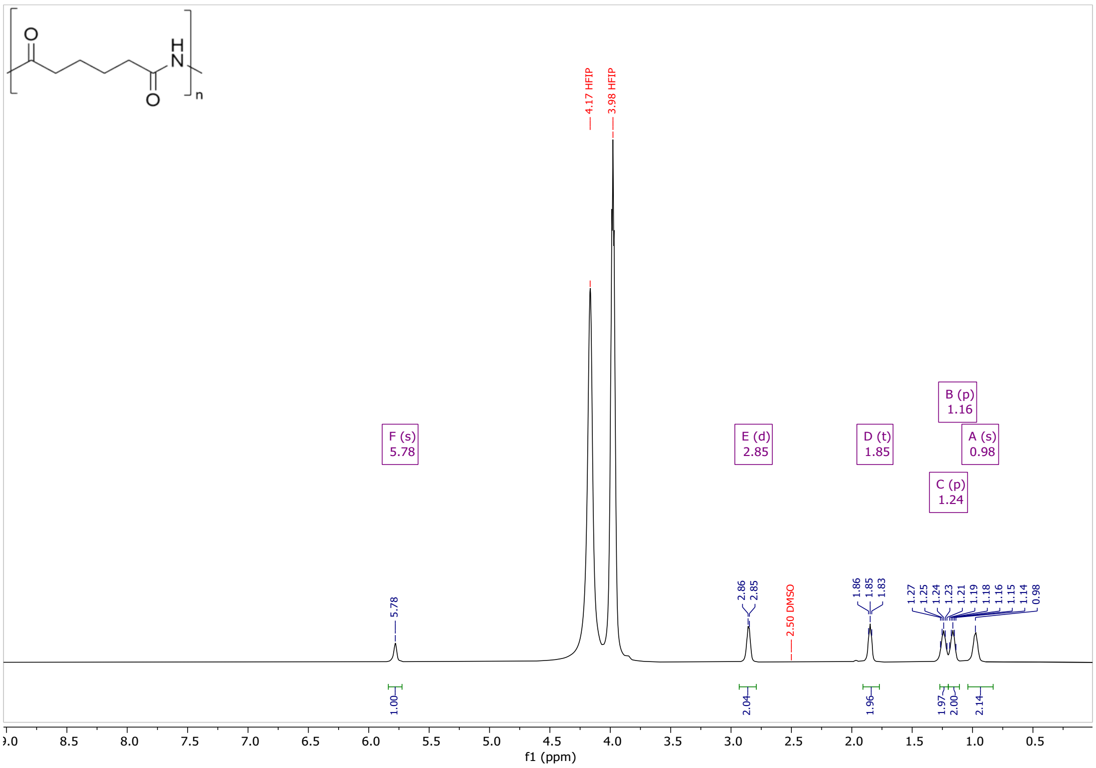

Figure S140 ^{1}H NMR of nylon 6 after dissolution and regeneration from HFIP

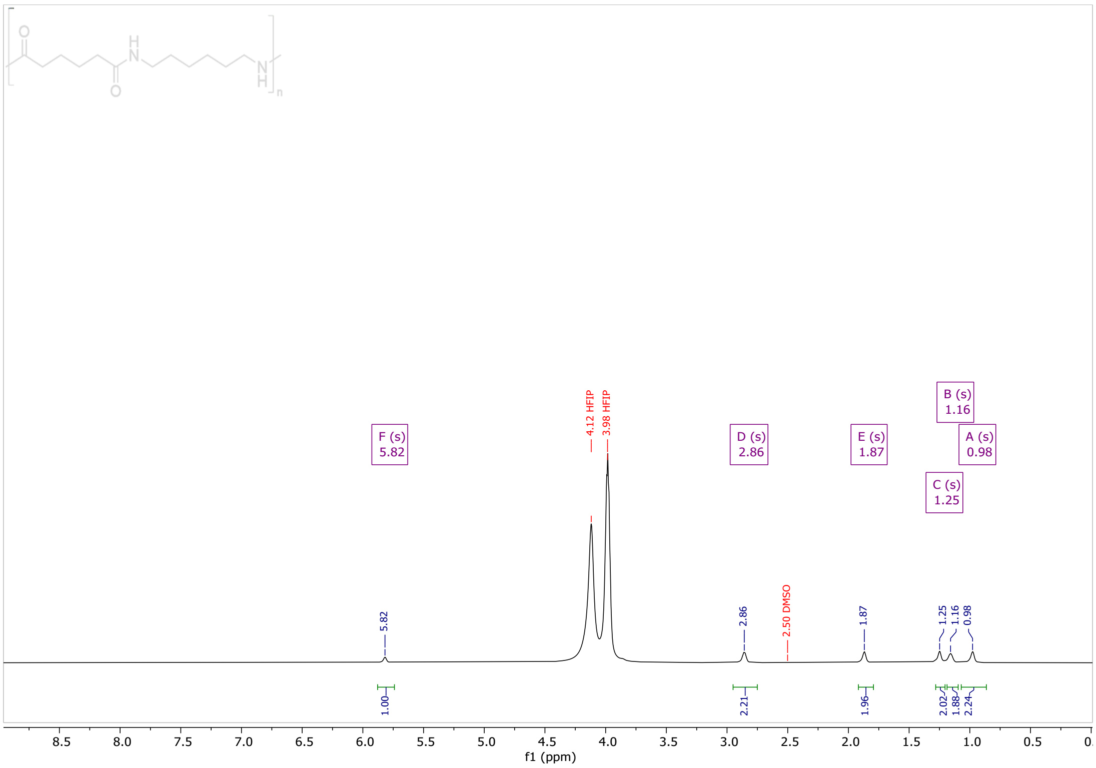

Figure S141 ^{1}H NMR of nylon 6,6 (P) after dissolution and regeneration from HFIP

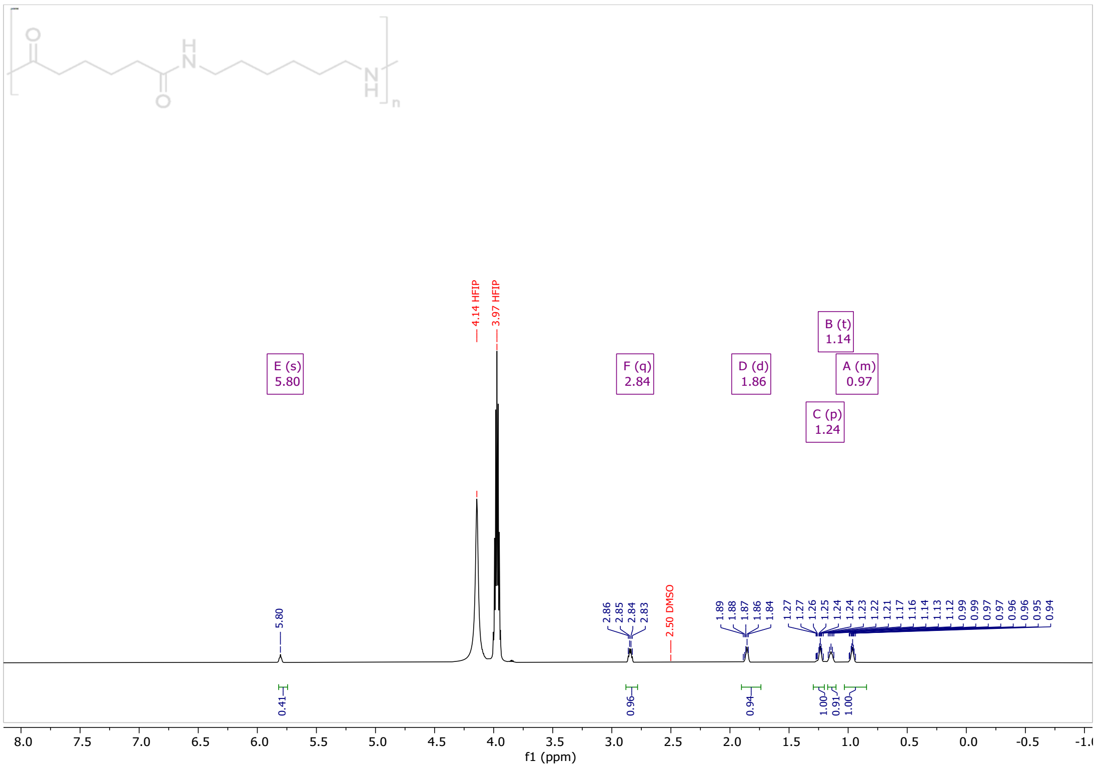

Figure S142 ¹H NMR of nylon 6,6 (M) after dissolution and regeneration from HFIP

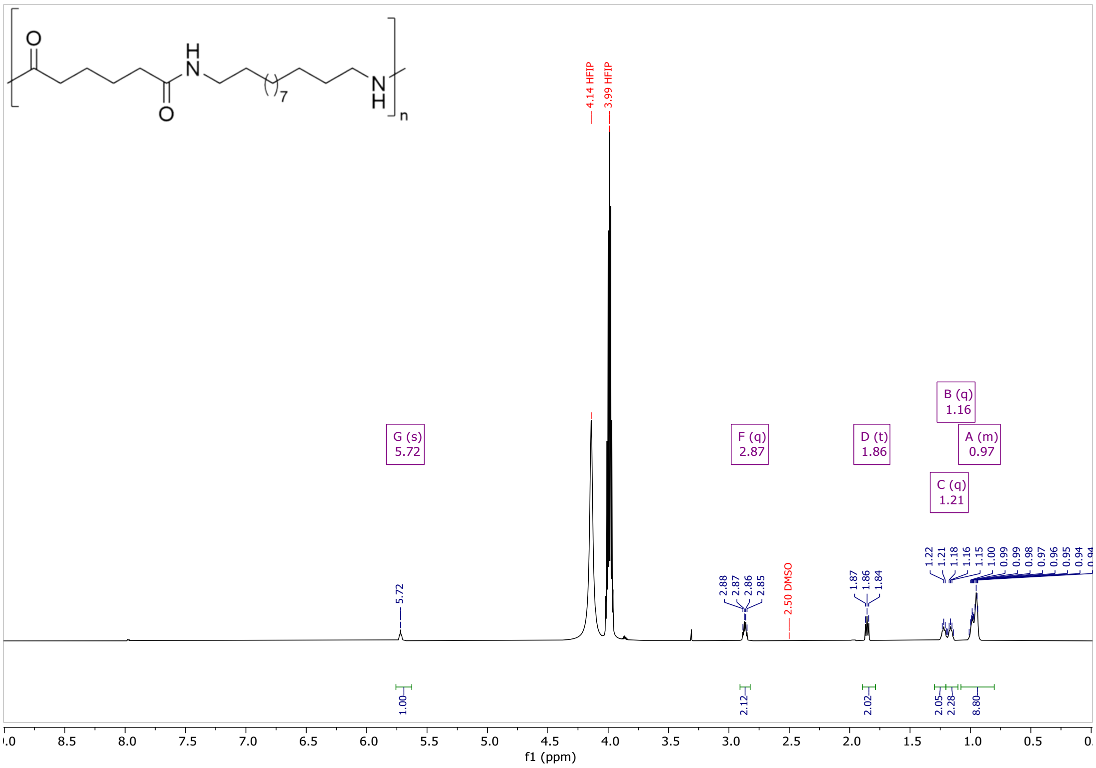

Figure S143 ¹H NMR of nylon 6,12 after dissolution and regeneration from HFIP

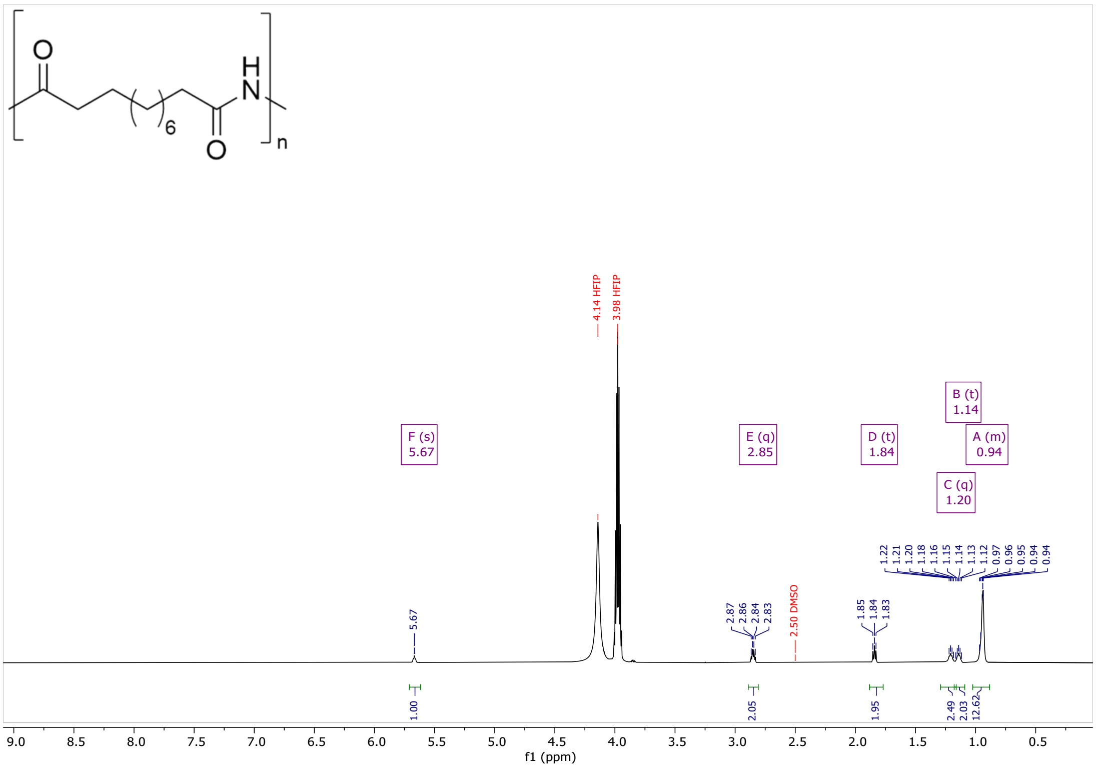

Figure S144 ¹H NMR of nylon 11 after dissolution and regeneration from HFIP

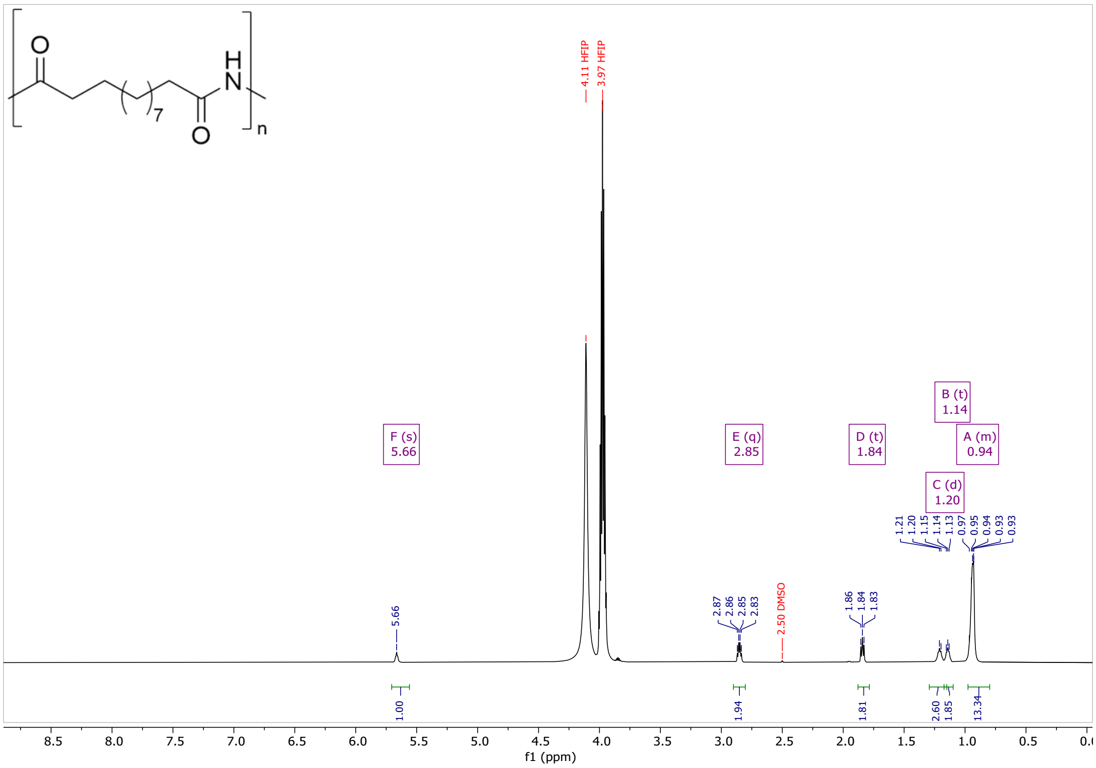

Figure S145 ¹H NMR of nylon 12 after dissolution and regeneration from HFIP

References

1 E. Gazagnaire, J. Helminen, A. W. T. King, T. Golin Almeida, T. Kurten and I. Kilpeläinen, *RSC Adv*, 2024, **14**, 12119–12124.
